# Thermochemical CO_2_ Reduction Catalyzed by Homometallic and Heterometallic Nanoparticles Generated from the Thermolysis of Supramolecularly Assembled Porous Metal-Adenine Precursors

**DOI:** 10.1021/acs.inorgchem.3c02830

**Published:** 2023-10-09

**Authors:** Jon Pascual-Colino, Quaid Johar Samun Virpurwala, Sandra Mena-Gutiérrez, Sonia Pérez-Yáñez, Antonio Luque, Garikoitz Beobide, Vijay K. Velisoju, Pedro Castaño, Oscar Castillo

**Affiliations:** † Department of Organic and Inorganic Chemistry, University of the Basque Country, UPV/EHU, P.O. 644, Bilbao E-48080, Spain; ‡ Multiscale Reaction Engineering, KAUST Catalysis Center (KCC), King Abdullah University of Science and Technology (KAUST), Thuwal 23955-6900, Saudi Arabia; § BCMaterials, Basque Center for Materials, Applications and Nanostructures, UPV/EHU Science Park, Leioa E-48940, Spain

## Abstract

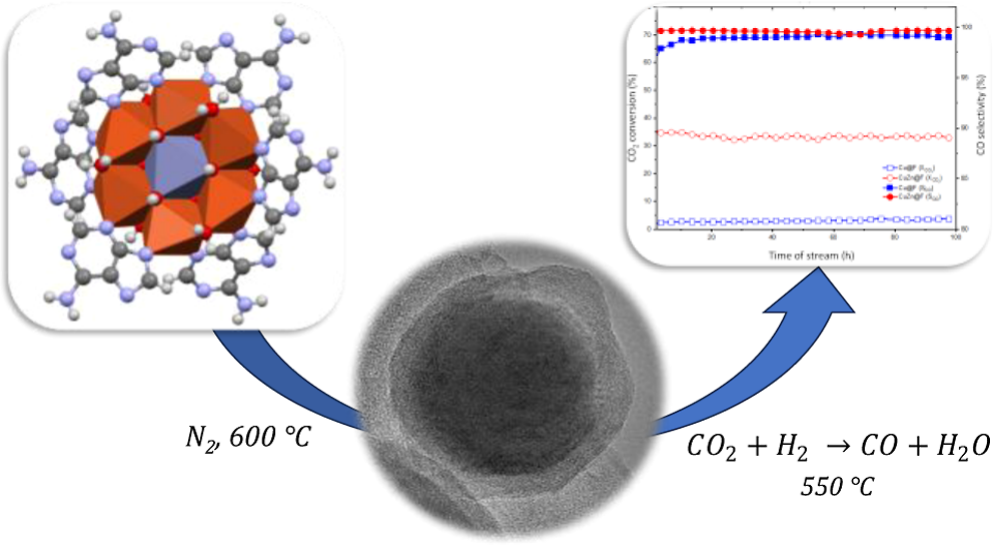

A family of unprecedented supramolecularly assembled porous metal–organic compounds (SMOFs), based on [Cu_6_M(μ-adeninato)_6_(μ_3_-OH)_6_(μ-H_2_O)_6_]^2+^ cations (M^II^: Cu, Co, Ni, and Zn) and different dicarboxylate anions (fumarate, benzoate, and naphthalene-2,6-dicarboxylate), have been employed as precursors of catalysts for the thermocatalytic reduction of CO_2_. The selected metal–organic cation allows us to tune the composition of the SMOFs and, therefore, the features and performance of the final homometallic and bimetallic catalysts. These catalysts were obtained by thermolysis at 600 °C under a N_2_ atmosphere and consist of big metal particles (10–20 μm) placed on the surface of the carbonaceous matrix and very tiny metal aggregates (<10 nm) within this carbonaceous matrix. The latter are the most active catalytic sites for the CO_2_ thermocatalytic reduction. The amount of this carbonaceous matrix correlates with the organic content present in the metal–organic precursor. In this sense, CO_2_ thermocatalytic reduction experiments performed over the homometallic, copper only, catalysts with different carbon contents indicate that above a certain value, the increase of the carbonaceous matrix reduces the overall performance by encapsulating the nanoparticles within this matrix and isolating them from interacting with CO_2_. In fact, the best performing homometallic catalyst is that obtained from the precursor containing a small fumarate counterion. On the other hand, the structural features of these precursors also provide a facile route to work with a solid solution of nanoparticles as many of these metal–organic compounds can replace up to 1/7 of the copper atoms by zinc, cobalt, or nickel. Among these heterometallic catalysts, the best performing one is that of copper and zinc, which provides the higher conversion and selectivity toward CO. XPS spectroscopy and EDX mappings of the latter catalyst clearly indicate the presence of Cu_1–*x*_Zn_*x*_ nanoparticles covered by small ZnO aggregates that provide a better CO_2_ adsorption and easier CO release sites.

## Abstract 2

Supramolecularly assembled porous metal–organic compounds (SMOFs), based on [Cu_6_M(μ-adeninato)_6_(μ_3_-OH)_6_(μ-H_2_O)_6_]^2+^ cations (M^II^: Cu, Co, Ni, and Zn) and different dicarboxylate anions (fumarate, benzoate, and naphthalene-2,6-dicarboxylate), have been employed as precursors of catalysts for the thermocatalytic reduction of CO_2_. The selected metal–organic cation and organic counterion allow us to tune the characteristics of the features and performance of the final homometallic and bimetallic catalysts.

## 1 Introduction

The most studied options for the remediation of the increasing CO_2_ concentration in the atmosphere involve storage, separation, and valorization into valuable chemicals and fuels. The latter option still requires more research to improve its viability and to move toward large-scale processing. In principle, CO_2_ can be valorized into products such as CO, CH_4_, HCOOH, H_2_C_2_O_4_, and CH_3_OH, among others.^1−4^ CO produced by reverse water-gas shift offers high flexibility as it can be used in both methanol and Fischer-Tropsch syntheses.^5−7^ However, this reaction requires high temperatures and the conversion is equilibrium-limited.^8^

All of the technologies described above require the use of catalysts, usually metal oxides or metal nanoparticles, in order to reduce the energy barrier penalty associated with these transformations.^9−13^ The use of metal–organic precursors for the preparation of these catalytically active nanoparticles has long been well documented in literature.^14−16^ In this sense, sol–gel resins with stochastic distribution of metals and metal–organic precursors with an ordered crystal structure and well-defined formula have been the most developed approaches.^17−22^ The porous nature of the metal–organic precursor helps to avoid the agglomeration of the metal nanoparticles that results from the thermolysis, as it has been well reported for MOFs.^22−24^ On the other hand, the amount of organic matter incorporated in the precursor also plays a crucial role in determining the amount of carbonaceous matrix. This helps to avoid sintering of the nanoparticles but in many cases at the expense of their encapsulation/isolation. Although there have been studies on the implementation of the latter method for the preparation of nanoparticles from heterometallic systems, it is more complicated and less common.^24^

**Figure 1:**
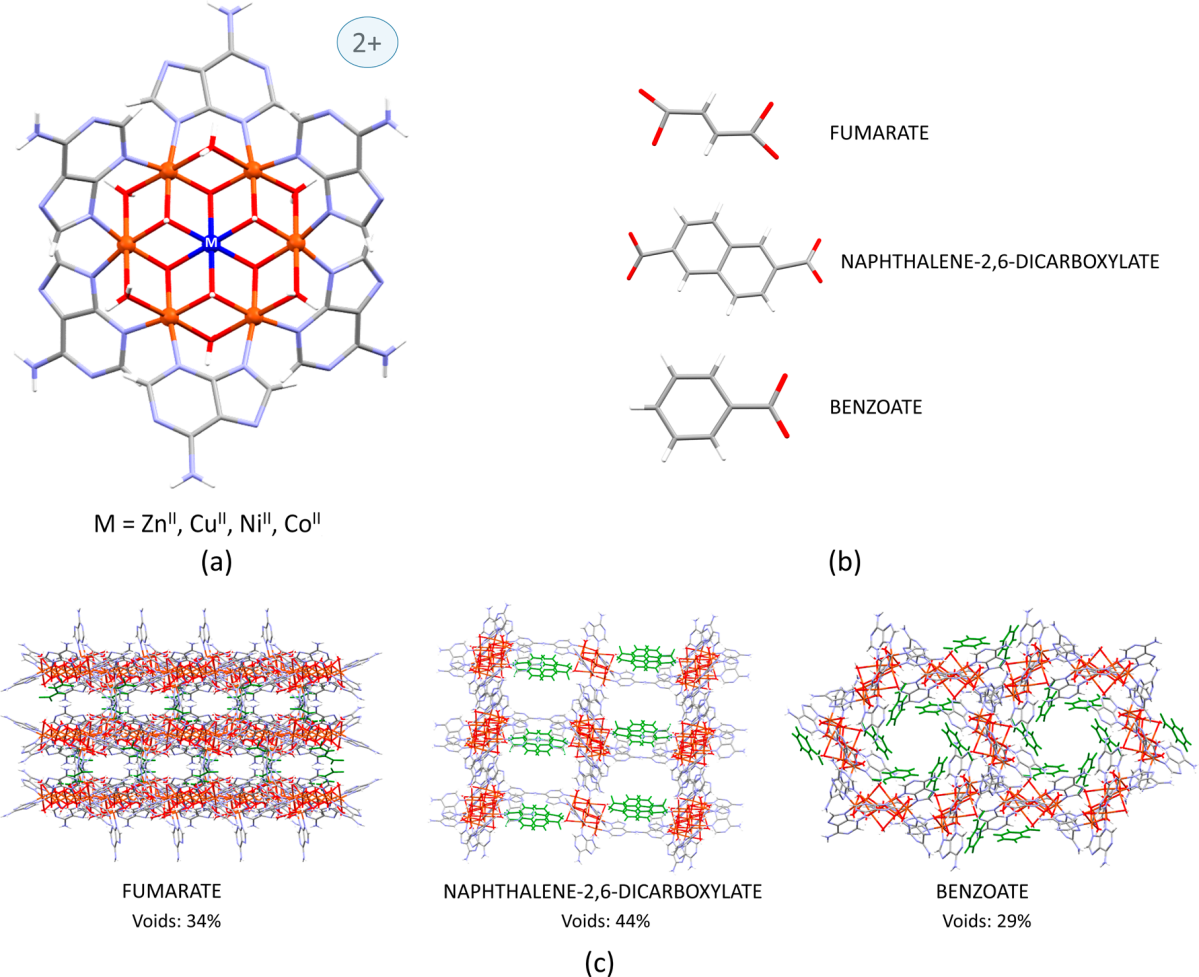
(a) Heptameric cluster depicting the targeted replacement of the central metal position. (b) Selected organic counterions to stabilize the heptameric cationic entity. (c) Porous packing of the heptameric entities with the organic counterions colored in green.

Taking into account all the above-mentioned, this work explores for the first time an emerging class of porous metal–organic materials, known as supramolecularly assembled metal–organic frameworks (SMOFs), to obtain homo- and heterometallic nanosized catalysts that are useful for the thermocatalytic hydrogenation of CO_2_. These structures have wheel-shaped heptanuclear [Cu_6_M(μ-adeninato)_6_(μ_3_-OH)_6_(μ-H_2_O)_6_]^2+^ cationic discrete units and organic carboxylate counterions assembled through π-stacking interactions and hydrogen bonding. The heptameric unit has the peculiarity of containing two well-differentiated metal environments: the central one with a regular MO_6_ environment and the six peripheral ones with a CuO_4_N_2_ coordination sphere showing a remarkable Jahn-Teller tetragonal distortion. This difference between the two environments makes it possible to obtain heterometallic structures in which we can place several transition metals such as M^II^ = Cu, Co, Ni, and Zn in the central position, but only Cu^2+^ in the six peripheral positions (Figure 1).^25,26^ These structures have two characteristics particularly suitable for their use as precursors of metallic nanoparticles: (i) the adenine nucleobase, which is a good reducing agent, facilitating the obtaining of the metallic nanoparticles by simple thermolysis under an inert atmosphere such as He, N_2_, or Ar; (ii) the high specific area, porosity, and excellent distribution of the active sites within the host matrix of the supports derived from these frameworks. The incorporation of counterions to compensate for the charge of the heptameric units is necessary due to the cationic nature of these structures.

More precisely, herein, we compare three Cu homometallic structures derived from the thermolysis of [Cu_6_Cu(μ-adeninato)_6_(μ_3_-OH)_6_(μ-H_2_O)_6_]^2+^ stabilized with different sized counterions: fumarate (C_4_H_2_O_4_^2–^), naphthalene-2,6-dicarboxylate (C_12_H_6_O_4_^2–^), and benzoate (C_7_H_5_O_2_^–^): [Cu_7_(μ-adeninato)_6_(μ_3_-OH)_6_(μ-H_2_O)_6_](fumarate)·∼25H_2_O (**Cu_F**), [Cu_7_(μ-adeninato)_6_(μ_3_-OH)_6_(μ-H_2_O)_6_](naphthalene-2,6-dicarboxylate)·∼32H_2_O (**Cu_N**), and [Cu_7_(μ-adeninato)_6_(μ_3_-OH)_6_(μ-H_2_O)_6_](benzoate)_2_·∼17H_2_O (**Cu_B**). It will make it possible to analyze the effect of increasing the organic content of the precursor on the final product and its influence on the catalytic properties.^18^ Normally, when thermal decomposition is carried out in an inert atmosphere, some of the organic matter ends up in the final product as more or less graphitized carbon, which can have either a positive or a negative effect on the catalytic activity of the catalyst. This fact has been much discussed since the surface area of the final product increases with the amount of carbon present, resulting in a better dispersion of the metallic nanoparticles and less sintering. However, access to these catalytically active metallic nanoparticles may be blocked. Indeed, the best thermocatalytic results are obtained with the smaller fumarate counterion, indicating that the risk of complete encapsulation of the metal nanoparticles during thermolysis is more relevant than the benefits that a better dispersion of the nanoparticles could bring.

Once this parameter has been analyzed and based on the herein explained positive result of the fumarate counterbalanced homometallic precursor, three new heterometallic compounds have been synthesized with this counterion: [Cu_6_Co(μ-adeninato)_6_(μ_3_-OH)_6_(μ-H_2_O)_6_](fumarate)·24H_2_O (**CuCo_F**), [Cu_6_Ni(μ-adeninato)_6_(μ_3_-OH)_6_(μ-H_2_O)_6_](fumarate)·19H_2_O (**CuNi_F**), and [Cu_6_Zn(μ-adeninato)_6_(μ_3_-OH)_6_(μ-H_2_O)_6_](fumarate)·24H_2_O (**CuZn_F**). These compounds are aimed at analyzing the possible synergic effects carried out on the CO_2_ thermal reduction by the presence of a second transition metal in the nanoparticles.

All in all, this study has allowed to study the effect of the carbon content in the organic counterion and the presence of a second transition metal on the thermally generated catalysts and their performance on the reverse water gas shift reaction.

## 2 Experimental Section

### 2.1 Synthesis of Compound Cu_N

0.122 g (0.5 mmol) sample of Cu(NO_3_)_2_·3H_2_O dissolved in 10 mL of water was added to 0.081 g (0.6 mmol) of adenine dissolved in 20 mL of an aqueous methanolic 1:1 hot solution (50 °C). The obtained blue solution (pH ∼ 4.0) was basified to pH ∼ 8.8 with NaOH under continuous stirring. Over the obtained purple solution, 0.1405 g (0.65 mmol) of naphthalene-2,6-dicarboxylic acid dissolved in 20 mL of water (30 °C) and basified with NaOH (pH ∼ 9.1) was added. The resulting purple solution was left to evaporate at room temperature (18 °C). Blue crystals were obtained after 4 days (Figure S1). Yield: 85%. Main IR features in Figure S17 (cm^–1^; KBr pellets): 3440vs, 3340w, 3190vs, 1640vs, 1600m, 1540s, 1490m, 1460vs, 1400vs, 1340s, 1270m, 1200s, 1040m, 930w, 790m, 740m, 550m. The synthesis of structure **Cu_B** follows a similar procedure, which has been described in a previous work.^27^

### 2.2 Synthesis of Compounds Cu_F

The procedure described above is employed but using 0.170 g (0.7 mmol) of Cu(NO_3_)_2_·3H_2_O and 0.1405 g (0.65 mmol) of fumaric acid. Blue crystals were obtained after 3 days (Figure S1). Yield: 75%. Main IR features (cm^–1^; KBr pellets): 3430vs, 3210s, 2920w, 1640vs, 1610m, 1550s, 1500w, 1470s, 1400s, 1380m, 1306m, 1195s, 1031m, 970m, 933w, 795m, 740m, 560m.

In the case of compounds **CuCo_F**, **CuNi_F**, and **CuZn_F**, the same overall procedure is followed but using a mixture of 0.7 mmol Cu(NO_3_)_2_·3H_2_O (0.170 g) and 0.6 mmol Co(NO_3_)_2_·6H_2_O (0.175 g) for compound **CuCo_F**, Ni(NO_3_)_2_·6H_2_O (0.174 g) for compound **CuNi_F**, and Zn(NO_3_)_2_·6H_2_O (0.178 g) for compound **CuZn_F**, respectively. Green (**CuCo_F**), light blue (**CuNi_F**), and dark blue (**CuZn_F**) crystals were obtained after 4–7 days (Figure S1). Data for compound **CuCo_F**, yield: 48%. Main IR features (cm^–1^; KBr pellets): 3360vs, 3210vs, 2920w, 1640vs, 1610s, 1550s, 1500w, 1460vs, 1390vs, 1370s, 1300m, 1200vs, 1150s, 1030m, 980m, 930w, 790m, 740m, 560m. Data for compound **CuNi_F**, yield: 45%. Main IR features (cm^–1^; KBr pellets): 3360vs, 3200vs, 2920w, 1640vs, 1600w, 1550vs, 1500w, 1460vs, 1400vs, 1340s, 1310m, 1200s, 1150m, 1030m, 980m, 940w, 800m, 740, 550m. Data for compound **CuZn_F**, yield: 65%. Main IR features (cm^–1^; KBr pellets): 3350vs, 3200vs, 2920w, 1640vs, 1600w, 1550vs, 1500w, 1460vs, 1400vs, 1340s, 1310, 1200vs, 1140s, 1030m, 980m, 940w, 800m, 740m, 550m.

### 2.3 Catalyst Preparation by Pyrolysis

300 mg of the fresh SMOFs (**Cu_F**, **Cu_N**, **Cu_B**, **CuCo_F, CuNi_F**, and **CuZn_F**) was introduced in a tubular furnace (Nabertherm) to perform its thermolysis in a four-stepped procedure. It starts heating the sample from room temperature to 600 °C at 5 °C·min^–1^ in a N_2_ atmosphere. The residue is then kept at 600 °C for 8 h, after which the oven is switched off and left to cool at room temperature to the initial 25 °C while maintaining the N_2_ atmosphere. Finally, the obtained samples (**Cu@F**, **Cu@N**, **Cu@B**, **CuCo@F**, **CuNi@F**, and **CuZn@F**) are kept in an oxygen atmosphere for 1 h.

### 2.4 Characterizations

Elemental analysis was performed by means of ICP-OES (Varian, Inc./Agilent model 7200-ES). For microwave digestion, approximately 10 mg of catalyst was taken in a mixture of 1 mL of HCl, 3 mL of HNO_3_, and 1 mL of HF and subjected to a microwave-assisted heating program with 15 min ramp time and 30 min hold time at 1000 W and 220 °C.

*Thermal analysis* was carried out using a gas controller GC 200 model TGA (Mettler Toledo) device for thermogravimetric measurements. About 10 mg of the as-synthesized sample were placed in an Al_2_O_3_ crucible and heated up from 30 to 850 °C with a heating rate of 10 °C min^–1^ under a 30 mL min^–1^ nitrogen atmosphere. An empty crucible was used as reference material.

*Single*-*crystal X*-*ray diffraction* data for structure determination were collected on Agilent Technologies Supernova diffractometers (λMο Kα = 0.71073 Å for **Cu_N**, **CuCo_F**, **CuNi_F**, and **CuZn_F**; λCu Kα = 1.54184 Å for **Cu_F**). The data reduction was done with the CrysAlisPro program.^28^ Crystal structures were solved by direct methods using the SIR92^29^ and SHELX^30^ programs and refined by full-matrix least-squares on *F*^2^ including all reflections. The high disorder that solvent molecules present precluded their modeling and, as a consequence, the electron density at the voids of the crystal structure was subtracted from the diffraction data by the SQUEEZE method^31^ as implemented in PLATON.^32^ Powder X-ray diffraction (PXRD) measurements were performed in a Bruker D8 ADVANCE diffractometer equipped with a Bragg-Brentano geometry fitted with a copper tube operating at 40 kV and 40 mA. Diffractograms were acquired over a 2θ range of 10–80° with a step size of 0.1° and a scan speed of 0.5 s per step. ICCDs powder diffraction file (PDF-4+, 2019) database was used for phase identification.^33^

*Scanning electron microscopy* (SEM) studies were carried out on a Hitachi TM3000 microscope operated at 5 kV and coupled to an energy X-ray spectrometer. Specimens were mounted on conductive carbon adhesive tabs.

*Transmission electron microscopy* (TEM) studies of fresh and used catalysts were performed on a TECNAI G2 20 TWIN operated at 200 kV and equipped with LaB_6_ filament and an energy-dispersive X-ray (EDX) spectrometer. The samples for the TEM were prepared by dispersing a small amount of the catalytic material onto a TEM copper grid (300 Mesh) covered by a holey carbon film.

*X*-*ray photoelectron spectroscopy* (XPS) measurements were performed in a SPECS system (Berlin, Germany) equipped with a Phoibos 150 1D-DLD analyzer and an Al Kα monochromatic radiation source (1486.7 eV). An initial analysis was carried out to determine the elements present (wide scan: step energy 1 eV, dwell time 0.1 s, pass energy 80 eV) and detailed analyses of the detected elements were performed (detail scan: step energy 0.08 eV, dwell time 0.1 s, pass energy 30 eV) with an electron exit angle of 90°. The spectrometer was previously calibrated with Ag (Ag 3d_5/2_, 368.26 eV). Spectra were fitted using CasaXPS 2.3.16 software, which models Gauss–Lorentzian contributions, after background subtraction (Shirley).^34^ The concentrations were calculated by correcting the values with relative atomic sensitivity factors (Scofield). In order to reproduce the initial activation for the reverse water gas shift experiments, the catalysts were reduced in a dedicated chamber within the equipment using a 300 mL/min continuous flux of H_2_/Ar (20%, 1 bar) with heating up the sample from room temperature to 450 °C in 1 h and keeping it at that temperature for an additional 1 h.

### 2.5 Reverse Water Gas Shift Experiments

Catalytic tests were performed using a Flowrence Avantium parallel reactor consisting of 16 tubular fixed-bed quartz reactors (2 mm ID, length 300 mm). Reactors are placed in a furnace, and the flow is distributed equally over the 16 channels by means of a microfluidic glass distributor. In each reactor, around 0.05 g of sieved catalyst particles (150–300 μm) were loaded onto a 9.5 cm long coarse SiC (particle grit 40) bed that ensures the catalyst bed lies on the isothermal zone of the reactor. One reactor was always used without a catalyst as a blank. Before catalytic run, the catalyst is reduced in situ at 450 °C for 1 h under a H_2_ atmosphere. Reactions were performed with a mixed feed containing 20 vol % of CO_2_ and 80 vol % of H_2_ in the temperature range of 350–550 °C. In addition, 0.5 mL·min^–1^ of He was mixed with the feed and used as an internal standard.

Product analyses were performed in an Agilent 7890B GC equipped with two sample loops with TCD and 2 FIDs detectors. After the loops were flushed for 15 min, the products are injected. One sample loop is directed toward the TCD channel with 2 Haysep precolumns and a MS5A column, where He, H_2_, CH_4_, and CO are separated. Gases that have retention times longer than those of CO_2_ on the Haysep column (column 4 Haysep Q 0.5 m G3591-80,023) are backflushed. Further separation of permanent gases is done on another Haysep column (column 5 Haysep Q 6 Ft G3591-80,013) to separate CO_2_ before going to an MS5A column. The other sample loop is directed toward an Innowax precolumn (5 m, 0.2 mm OD, 0.4 μm film). In the first 0.5 min of the method, the gases coming from the precolumn are sent to the Gaspro column (Gaspro 30 M, 0.32 mm OD). After 0.5 min, the valve is switched and gases are sent to an Innowax column (45 m, 0.2 mm OD, 0.4 μm). Products from both columns are analyzed through a FID. The Gaspro column separates C_1_–C_8_ paraffins and olefins, while the Innowax column separates oxygenates and aromatics.

The conversion (*X*), selectivity (*S*_*i*_), yield (*Y*_*i*_), and space-time yield (STY_*i*_) of an individual or lumped species *i* were, respectively, defined as
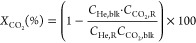
1
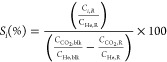
2
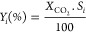
3
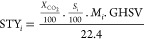
4where *C*_He,blk_, *C*_He,R_, *C*_CO_2_,blk_, and *C*_CO_2_,R_ are the concentrations of He in the blank, He in the reactor effluent, CO_2_ in the blank, and CO_2_ in the reactor effluent, respectively, *C*_*i*,R_ is the concentration of the product, *M*_*i*_ is the molecular weight of product *i*, and GHSV is the CO_2_ gas hourly space velocity in L·g_cat_^–1^·h^–1^. The concentrations of all species were determined by GC analysis.

## 3 Results and Discussion

### 3.1 Structural Characterization of the Precursors

The crystal structure of the supramolecular frameworks (**Cu_F**, **Cu_N**, or **Cu_B**) contains wheel-shaped heptanuclear cationic entities and carboxylate counterions [fumarate (**Cu_F**), naphthalene-2,6-dicarboxylate (**Cu_N**), and two times benzoate (**Cu_B**)]. The connectivity within the heptanuclear entities is essentially identical in such a way that a central [M^II^(OH)_6_]^4–^ core is connected to the six Cu^2+^ metal centers comprising the external ring, Figure 1a. The peripheral copper atoms are further connected through double μ-H_2_O and μ-adeninato-κ*N3*:κ*N9* bridges. The differences arise from the supramolecular assembly of these entities. In all cases, it is sustained by π-stacking interactions among the nucleobases and hydrogen bonds involving the carboxylate group and the coordinated hydroxide and water molecules. In addition, in structures containing dicarboxylate anions (**Cu_F** and **Cu_N**), these are sandwiched between two adeninato ligands from two adjacent heptameric entities. In structure **Cu_B**, where the monocarboxylate benzoate anion is employed, this sandwich-like arrangement is avoided since it is sterically hindered in these structures to establish a π-stacking interaction with two adeninato ligands that should be placed above and below the negatively charged only carboxylate group of benzoate. Therefore, the benzoate anions are in the inner part of the channels, establishing a single π-stacking interaction with just one adeninato ligand (Figure 1c). This last structure has been described in more detail before.^27^ The intermolecular interactions generate a 3D supramolecular porous architecture with the voids occupying a 34 (**Cu_F**), 44 (**Cu_N**), and 29% (**Cu_B**) of the total volume, as calculated by PLATON.^32^

**Figure 2:**
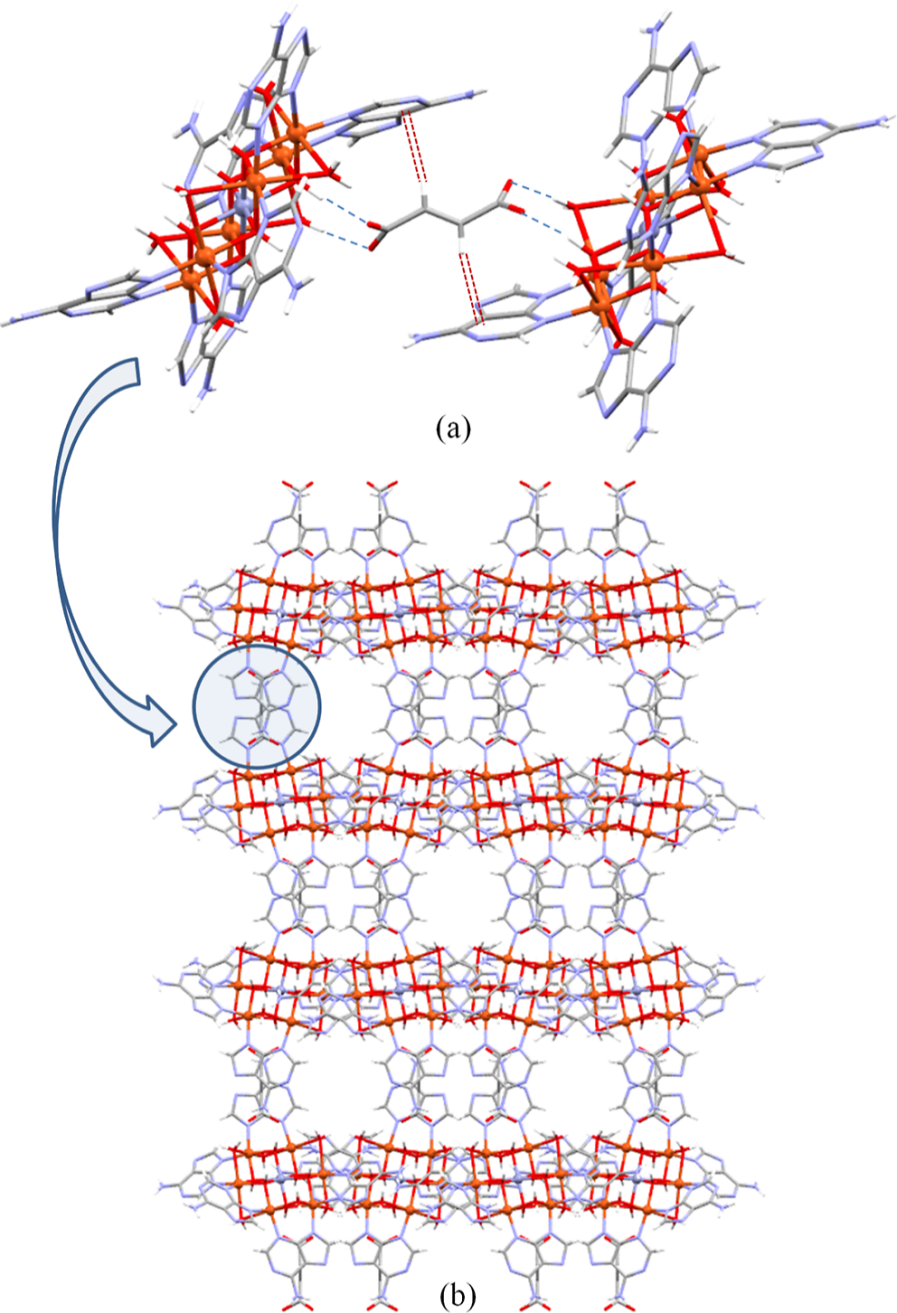
(a) Supramolecular interactions established by the fumarate anion in heterometallic structures. (b) Porous supramolecular architecture. Dashed blue lines: hydrogen bonding interactions; double dashed red lines: T-shaped interaction.

In the heterometallic fumarate anion-based structures (**CuCo_F**, **CuNi_F**, and **CuZn_F**), the supramolecular architecture differs from that of the homometallic Cu counterpart with a slightly different arrangement of the fumarate anions with respect to the adeninato ligands. The fumarate is still sandwiched between them, but its vinylic hydrogen atoms are pointing perpendicularly to the aromatic system of the adeninato ligand in a T-shaped π-interaction (Figure 2).

We attribute this difference to the modification of the M–OH bond distance in the core of the heptameric entity because of the different coordination environment taking place for the Jahn–Teller distortion of Cu^2+^ and the more regular Co^2+^, Ni^2+^, and Zn^2+^ metal centers. Although this modification changes the resulting supramolecular interactions as previously described, it does not change the porous nature of the heterometallic structures with 36, 33, and 36% of voids in its crystal structure, respectively.

### 3.2 Homometallic Cu Catalysts

**Figure 3:**
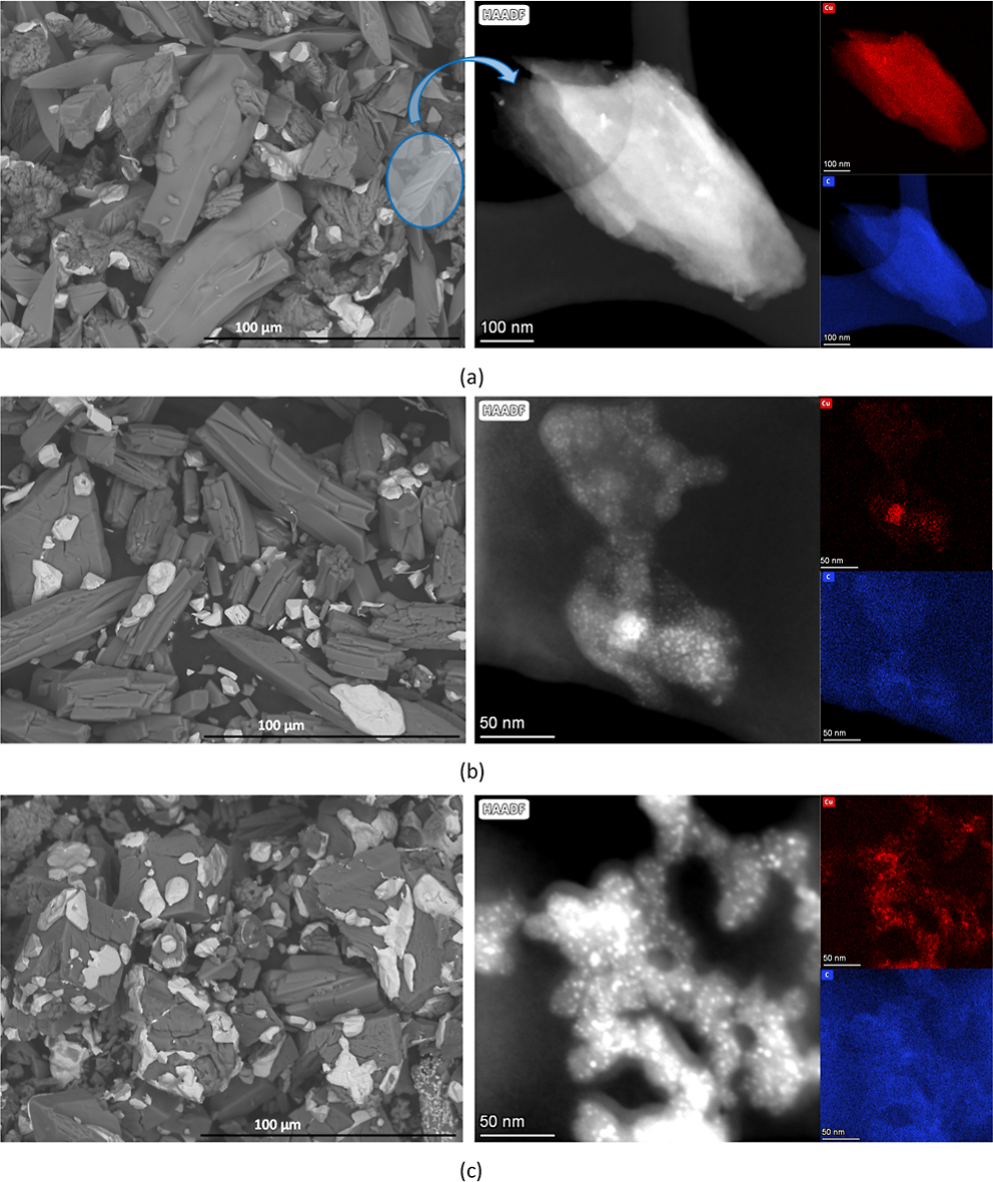
SEM (left) and high-angle annular dark field TEM (right) images and the corresponding EDX elemental maps for the catalyst derived from the homometallic compounds: (a) **Cu@F**, (b) **Cu@N**, and (c) **Cu@B**. TEM images provide closer insights into the carbonaceous matrix and the nanoparticles embedded within it.

SMOFs **Cu_F**, **Cu_N**, and **Cu_B** were subjected to the thermolysis procedure described in the Experimental section. X-ray diffraction data of the resulting bulk products (**Cu@F**, **Cu@N**, and **Cu@B**) shows a nonplanar background from which three peaks emerge at 2θ values of 43.3, 50.4, and 74.1° (the first one being the most intense) that agree with those expected for the metallic Cu^0^**fcc** phase (Figure S26). The metal content of all the three thermolysis products, determined by ICP-OES analysis, shows a progressive decrease, in the following order: **Cu@F** (fumarate counterion; Cu content = 45.3%) > **Cu@N** (naphthalene-2,6-dicarboxylate counterion; 39.2%) > **Cu@B** (two times benzoate counterion; 32.7%). Apparently, these results allow to conclude the greater the mass of the organic counterion in the precursor the smaller the metal content and bigger the contribution of the carbonaceous matrix in the resulting catalyst. Combining SEM and TEM images taken on the samples after the inert atmosphere thermolysis procedure, an overall picture of the resulting products is obtained. They consist of big metal particles (10–20 μm) placed on the surface of even greater carbonaceous particles (20–100 μm) and very tiny metal aggregates (<10 nm) within the carbonaceous matrix that will be probably the most active catalytic sites (Figure 3). TEM images focused on the carbonaceous matrix show a fine and homogeneous distribution of these very small aggregates of copper (below 10 nm for **Cu@N** and **Cu@B**; below 5 nm for **Cu@F**).

**Figure 4:**
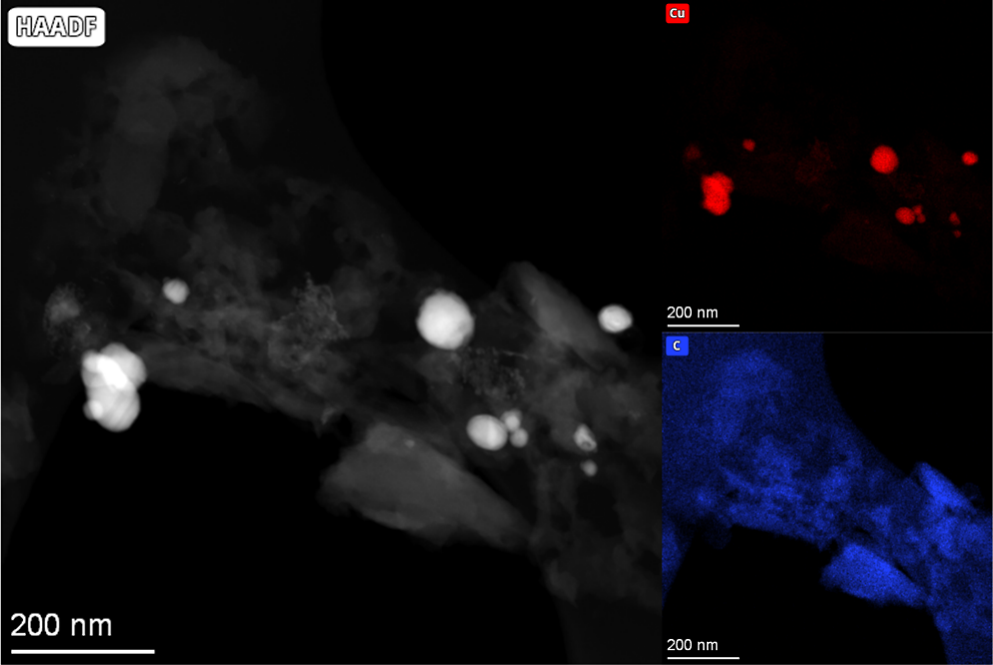
High-angle annular dark field TEM images (left) and the corresponding EDX elemental maps (right) for **Cu@F** after the thermocatalytic experiment (namely **Cu@F**_**p**_).

**Cu@F**, **Cu@N**, and **Cu@B** catalysts produced by the thermolysis of the precursor SMOFs (**Cu_F**, **Cu_N**, and **Cu_B**) were employed for the catalytic reverse water gas shift reaction of CO_2_. The CO_2_ conversion and CO selectivity over a temperature range of 350–550 °C have been reported in Figure S32 for the three catalysts. The conversion increases with the temperature, obtaining negligible values at 350 °C, below 5% at 450 °C, but significantly high conversion at 550 °C for the fumarate-based catalyst (**Cu@F**, 44%). These results also indicate that as the carbonaceous matrix increases, the CO_2_ conversion lowers, probably because when a great amount of the latter is present the copper nanoparticles are isolated from interacting with the CO_2_ molecules. The CO selectivity of the best-performing homometallic Cu catalyst (at 550 °C) is still at 85% (with a significant formation of CH_4_) which is below the selectivity provided by the typical Cu-based catalyst reported in the literature (>99%).^35^ The TEM analysis of the best performing homometallic Cu catalyst after the catalytic reaction (**Cu@F**_**p**_) shows that although the copper nanoparticles inside the carbonaceous matrix partially evolved into bigger particles, they are still well placed within the nanometric regime (10–50 nm; Figure 4). During the thermocatalytic experiment, the oxygen content present in the catalysts also disappears from both the carbonaceous matrix and the surface of the copper particles.

### 3.3 Heterometallic Cu–M (M: Co, Ni, and Zn) Catalysts

Based on the above-described results, the next step consists of improving the catalyst’s performance by introducing another metal such as Co, Ni, and Zn, which will be focused on SMOFs based on the fumarate anion to avoid the encapsulation effect. The syntheses were accordingly modified, as detailed in the synthesis section. Single crystal X-ray diffraction confirmed as previously predicted that this new metal center only accommodated at the central position of the cluster fixing a maximum for the copper replacement that can be achieved in these heterometallic precursors (14.3% atomic). The heterometallic precursors were subjected to the same thermolysis process as previously described. The metal content of the resulting new heterometallic catalyst (**CuCo@F**, **CuNi@F**, and **CuZn@F**) was determined by ICP–OES analysis (Cu: 38.2–39.0%; Co: 6.1%, Ni: 5.6%, and Zn: 5.3%, respectively). The total metal content agrees fairly well with that found for the homometallic analogue (**Cu@F**: 45%). Furthermore, the metal loadings of the benchmark Cu–Zn–Al catalyst,^36,37^ that will be employed in the CO_2_ thermocatalytic reduction experiment for comparative purposes was also measured (Cu: 43.7%; Zn: 15.6%; and Al: 3.6%).

**Figure 5:**
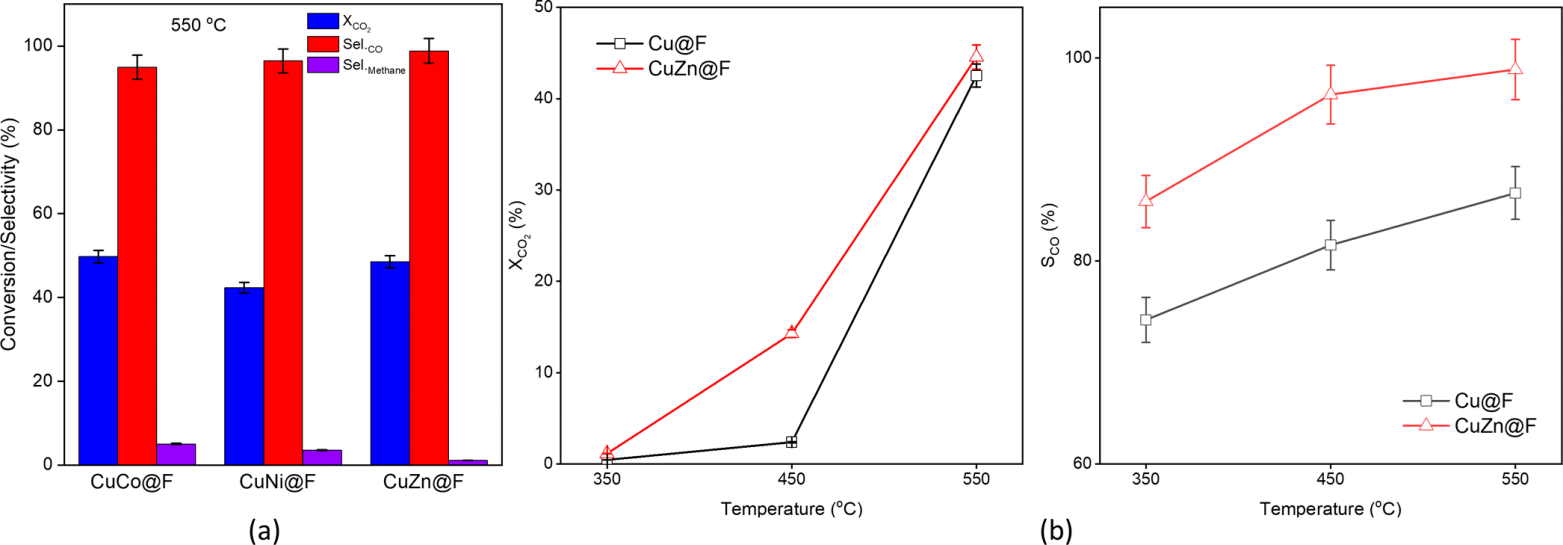
(a) Results of the thermocatalytic reduction of CO_2_ at 550 °C for the heterometallic catalysts: conversion (blue) and selectivity toward CO (red) and CH_4_ (purple) and (b) CO_2_ conversion and (c) selectivity of CO over the catalysts in the temperature range of 350–550 °C. Reaction conditions: 1 bar, 350–550 °C, GHSV = 19,000 mL·g_cat_^–1^·h^–1^.

The heterometallic catalysts were also tested in the Avantium reactor (Figures 5, S32). The CO_2_ conversion presents a significant improvement at 550 °C for all the heterometallic catalysts with values approaching those of the benchmark Cu–Zn–Al catalyst (conversion/selectivity: 46.6/95.5 **CuCo@F**, 43.1/96.6 **CuNi@F** and 44.9/98.6 **CuZn@F** vs 63.69/99.8 Cu–Zn–Al). As the **CuZn@F** catalyst provides the better selectivity toward CO while retaining a good conversion within the herein developed heterometallic catalysts, it was subjected to a deeper characterization.

The PXRD pattern shows the same features observed for the homometallic catalysts, with the presence of the same three peaks described for the previous ones that belong to the cubic phase of copper, Figure S26. The PXRD pattern of the catalyst remains unchanged after 50 h of the reverse water gas shift experiment, Figure S33. No additional peaks are observed, which probably means that the zinc atoms are incorporated into the Cu^0^ phase as a solid solution. This result agrees with the solubility limit reported for the metallic copper–zinc system.^38^

**Figure 6:**
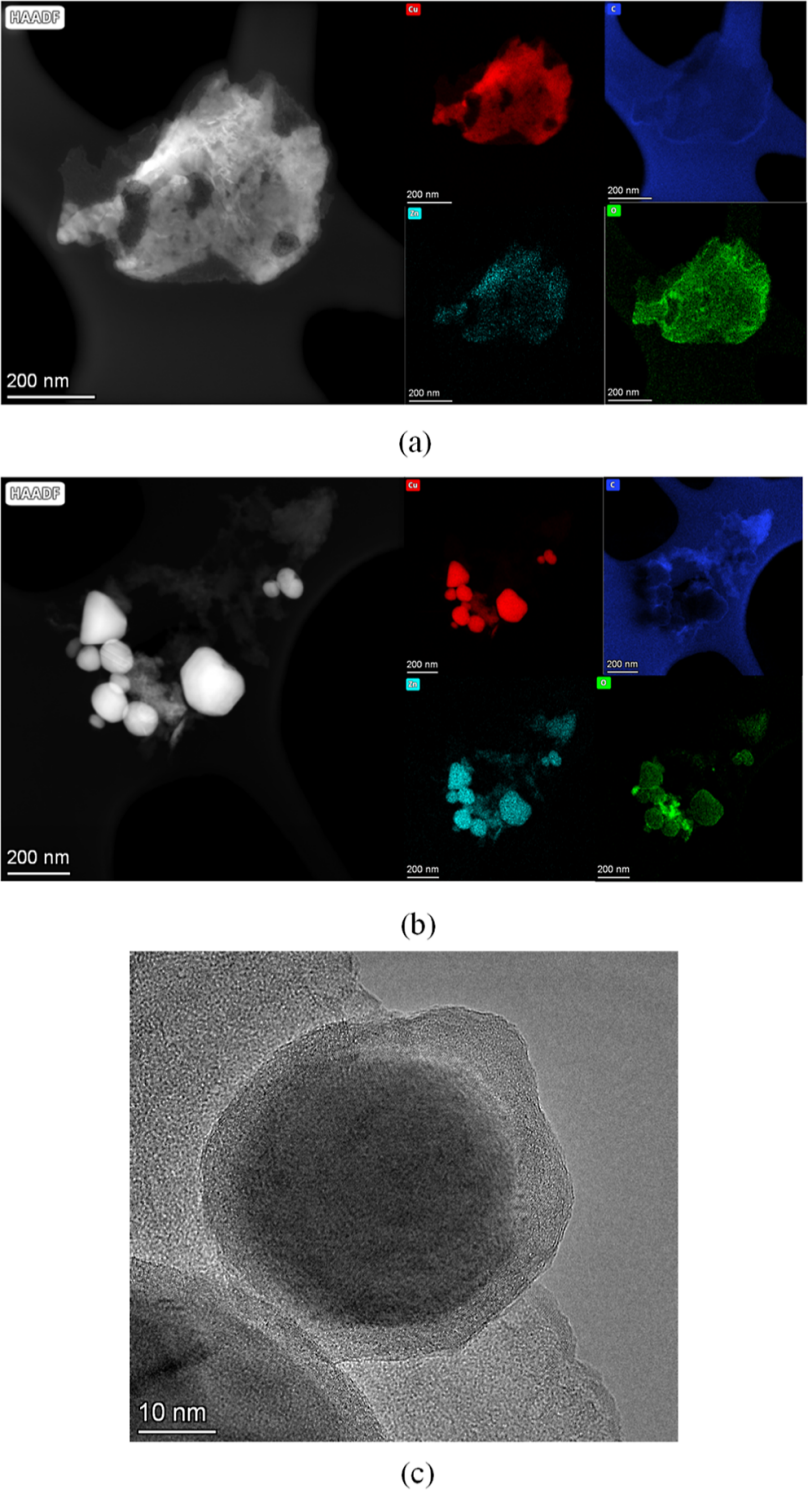
High-angle annular dark-field TEM images and the corresponding EDX elemental maps for the heterometallic Cu–Zn catalyst prior to (**CuZn@F**, a) and after the reverse water gas shift experiment (**CuZn@F**_**p**_, b). High magnification image of a nanoparticle embedded in the carbonaceous matrix of **CuZn@F**_**p**_ (c).

TEM images show an evolution from the very small nanoparticles (2–5 nm) inside the carbonaceous matrix that are obtained from the thermolysis process. The latter evolve during the reverse water gas shift reaction into 10–50 nm nanoparticles embedded in a carbonaceous matrix, as was also observed for the homometallic Cu counterparts (Figures 6, S27–S30). The elemental mapping indicates an even distribution of copper and zinc within these particles. Another interesting feature is the almost complete disappearance of oxygen from the carbonaceous matrix, as in the analogous homometallic catalyst, but it does not completely disappear from the metal particle surface.

**Figure 7:**
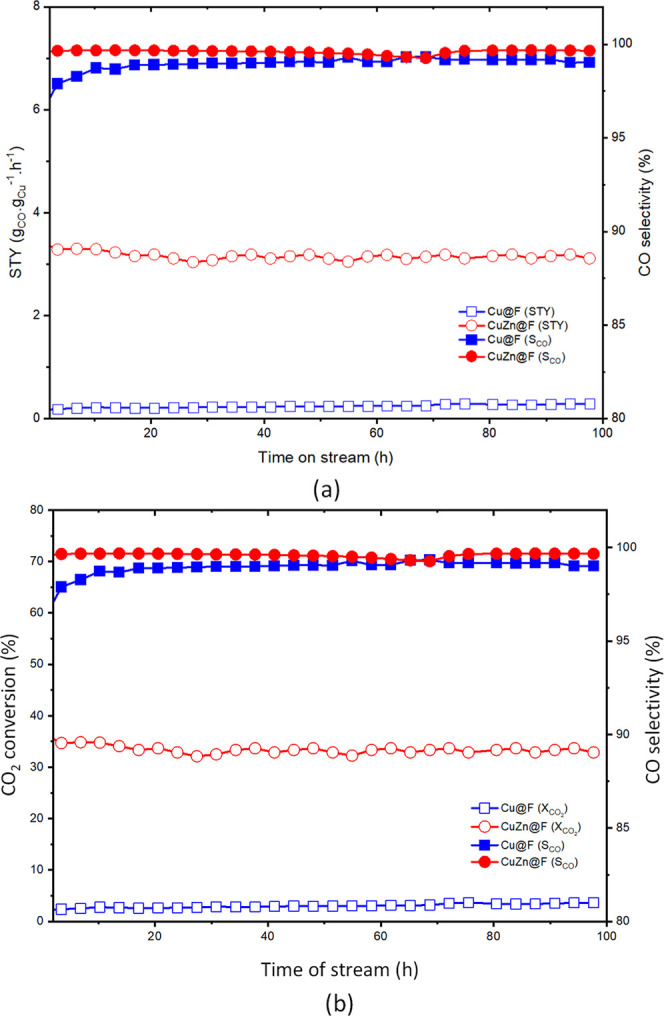
(a) Time on stream catalytic performance of **Cu@F** and **CuZn@F**. Reaction conditions: 1 bar, 550 °C, GHSV = 13,000 mL·g_cat_^–1^·h^–1^ and (b) CO_2_ conversion and CO selectivity over a 100 h run.

The time on stream (TOS) analysis for the studied catalysts, carried out at 550 °C for 100 h, indicates a time improving space time yield (STY) for **CuZn@F** (3.2 g_CO_·g^–1^·h^–1^/114 mmol_CO_·g^–1^·h^–1^; Figure 7). They provide a sharp contrast with those of the homometallic Cu counterpart (**Cu@F**). On the other hand, the very high selectivity toward CO remains stable during these long runs. It is worth mentioning that selectivity on CO_2_ reduction products is key for their incorporation into industrial processes.

To get deeper insights into the mechanism of CO_2_ conversion, it is necessary to employ a surface-specific technique such as XPS as bulk techniques such as the previously mentioned PXRD do only provide information on the bulk. In this sense, the XPS spectra of **Cu@F**_**p**_ were measured before and after its activation under an atmosphere of 5% H_2_ in helium with the same heating ramp (25–450 °C in 1 h) as performed during the catalytic experiments. This activation was performed on a dedicated chamber within the XPS equipment that allows direct transfer of the sample to the measurement chamber without exposure to the room atmosphere.

**Figure 8:**
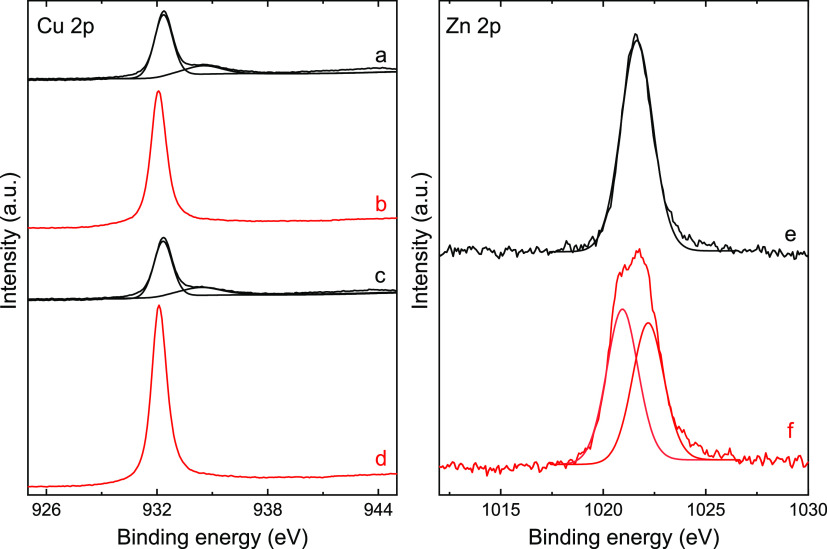
XPS spectra of Cu(2p_3/2_) (left) for **CuZn@F** before (a) and after (b) the activation procedure and for **Cu@F**_**p**_ before (c) and after (d) the activation procedure. XPS spectra of Zn(2p_3/2_) (right) for **CuZn@F** before (e) and after (f) the activation procedure.

The results shown in Figure 8 indicate that there are clear changes in the spectra prior to and after the activation procedure. In the homometallic Cu^II^ catalyst, the initial mixture at the surface level of Cu^2+^ (probably CuO; 2p_3/2_; 934.5 eV) and Cu^+^ (probably Cu_2_O; 2p_3/2_; 932.3 eV) converge in a single signal after the activation procedure assigned to Cu^0^ (2p_3/2_; 932.1 eV), Table S15. There is also an almost complete disappearance of oxygen signals that go from a 14% atomic ratio at the surface level to almost indistinguishable from the background (1.8%), in agreement with the aforementioned TEM–EDX analysis. In the case of the heterometallic Cu–Zn catalyst (**CuZn@F**), the same changes are observed for copper. The zinc signal prior to the activation (2p_3/2_; 1021.6 eV) is difficult to assign either to Zn^0^ or Zn^2+^, but chemically it seems logical to assign to Zn^2+^ as we have previously confirmed the appearance of Cu^2+^ which is more easily reduced than zinc. It would be present as a substitutional solid solution in the form of Cu_1–*x*_Zn_*x*_O. After the activation, this signal splits into two new ones, the first one with lower binding energy (2p_3/2_; 1020.9 eV) and another with higher binding energy (2p_3/2_; 1022.2 eV) that we have associated with the formation of Zn^0^ and ZnO, respectively. All of the above can be summarized in the following reactions
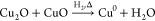
5
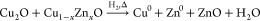
6

This proposal is also supported by the fact that the oxygen calculated at the surface is reduced during the activation but still falls far from disappearing (18.0 vs 7.2%). From these experiments, we can also conclude that there is a clear enrichment of zinc at the surface level in comparison with the amount found in the bulk material by the ICP Zn/Cu ratio: 0.126 vs 0.307–0.258).

Translating these results into a plausible hypothesis for the reaction mechanism requires an explanation for both the significant increase of the conversion rate of CO_2_ and selectivity toward CO when zinc is incorporated. In this sense, the presence of ZnO clusters at the surface of the metallic nanoparticles helps the adsorption of CO_2_ increasing the conversion. On the other hand, the increase of selectivity toward CO requires another explanation. Accordingly, the formation of CO must be understood as one of the first species in the reduction of CO_2_ and any increase of the selectivity toward this species seems to be due to factors that facilitate its desorption prior further reduction takes place. A relatively pristine Cu^0^ surface, as that of **Cu@F** after activation, seems favorable to retain the CO molecules through chemisorption once they are formed. However, at the surface of **CuZn@F** there are Cu^0^ (properly Cu_1–*x*_Zn_*x*_) and ZnO regions. As the temperature is raised, the mobility of the chemisorbed CO molecules increases, as well as their capacity to be desorbed, leading to the slight increase of conversion observed for **Cu@F**. However, at the surface of **CuZn@F** things are different. The Cu^0^ regions act as the reduction site but at high temperature when the chemisorbed CO molecules can move over the surface they are prone to find ZnO regions (or, perhaps zinc atoms generate a solid solution with copper) where the interaction with the CO molecules is going to be diminished facilitating their desorption and precluding its conversion to further reduced species, which turns into an increase of selectivity.^39^

## 4 Conclusions

The dependence found in the homometallic catalyst with respect to the carbonaceous content seems to indicate that the most active catalytic sites are those corresponding to the tiny nanoparticles inside the carbonaceous matrix. Note that for the big metallic particles (10–20 μm) placed outside the carbonaceous matrix such dependence would not be expected, and such good performance is rarely observed with such gross particle sizes. This fact is met except when the carbonaceous matrix is too abundant and starts encapsulating/isolating the nanometric particles inside it from the incoming CO_2_ and lowering the overall performance as observed for the series of the prepared homometallic copper catalysts. In addition, the carbonaceous matrix content that appears on the catalyst after the thermolysis can be directly correlated with the organic content of the metal–organic precursor, which provides a straightforward route for its control. Indeed, for the homometallic Cu catalysts, the best performance is for that containing the smallest counterion, i.e., fumarate.

These precursors also provide a new and facile route to work with nanoparticles based on metallic solid solutions. In fact, the central position of these SMOFs can be replaced with different metals (Co, Ni, and Zn). This approach allows obtaining Cu_1–*x*_M_*x*_ nanoparticles within the carbonaceous matrix that in the case of the catalyst, combining copper and zinc (**CuZn@F**) provides a very significant increase of the CO_2_ thermal reduction. This improvement in the selectivity toward CO has been attributed to the presence of ZnO aggregates within the surface of the Cu_1–*x*_Zn_*x*_ nanoparticles that provides stronger adsorption sites for CO_2_ and easier release sites for CO.

## Supporting Information Available

Brief description and images of the crystal structure of the five new compounds; analysis of porosity; Fourier transform infrared spectra of each one; thermogravimetric analysis; temperature variable PXRD experiments; X-ray photoelectron spectroscopy of pre and after reaction samples; X-ray diffraction patterns; and TEM and catalysis results (PDF)

## Abbreviations

SMOF: supramolecular metal–organic framework
MOF: metal–organic framework
STEM-EDX: scanning transmission electron microscopy-energy-dispersive X-ray
TEM-EDX: transmission electron microscopy-energy-dispersive X-ray
XPS: X-ray photoelectron spectroscopy
PXRD: powder X-ray diffraction
