# Supplementary material for: Thermochemical CO2 Reduction Catalyzed by Homometallic and Heterometallic Nanoparticles Generated from the Thermolysis of Supramolecularly Assembled Porous Metal-Adenine Precursors
Source: Inorg Chem. 2023 Oct 9;62(42):17444–53. doi: 10.1021/acs.inorgchem.3c02830 (PMC10598881; doi:10.1021/acs.inorgchem.3c02830)
Supplement: Supplementary file 1 — ic3c02830_si_001.pdf [file ic3c02830_si_001.pdf]

Supporting Information for

Thermochemical CO_2 reduction Catalyzed by Homometallic and Heterometallic Nanoparticles Generated from the Thermolysis of Supramolecularly Assembled Porous Metal-Adenine Precursors

Jon Pascual–Colino,[†,§] Quaid Johar Samun Virpurwala,[‡] Sandra Mena-Gutiérrez,[†] Sonia Pérez–Yáñez,[†,§] Antonio Luque,[†,§] Garikoitz Beobide,[†,§] Vijay K. Velisoju,[‡] Pedro Castaño,[,‡] Oscar Castillo,[*,†,§]*

pedro.castano@kaust.edu.sa; oscar.castillo@ehu.eus

[†]Department of Organic and Inorganic Chemistry, University of the Basque Country, UPV/EHU, P.O. 644, E–48080 Bilbao, Spain.

[‡]Multiscale Reaction Engineering, KAUST Catalysis Center (KCC), King Abdullah University of Science and Technology (KAUST), Thuwal, 23955–6900, Saudi Arabia.

[§]BCMaterials, Basque Center for Materials, Applications and Nanostructures, UPV/EHU Science Park, E–48940 Leioa, Spain.

KEYWORDS CO_2 utilization, reverse water gas shift reaction, supramolecularly assembled porous metal–organic frameworks (SMOF), nanoparticle–supported catalyst

S1. Crystal structure of compounds 3
S2. Analysis of porosity 24
S3. Fourier Transform Infrared Spectra (FTIR) 28
S4. Thermogravimetric analysis (TG) 32
S5. Temperature variable PXRD experiments (TDX). 36
S6. X-Ray Photoelectron Spectroscopy (XPS) 41
S7. Powder X-ray Diffraction (PXRD) 46
S8. Transmission electron microscopy (TEM) 48
S9. Catalysis results 53
S10. References 57

S1. Crystal structure of compounds

Crystallographic analysis

Single crystals of the four precursors were obtained (Figure S1) which allowed their structural characterization by means of single-crystal X-ray diffraction. The crystallographic data, the refinement conditions, and parameters of the resolution of compounds have been gathered in Table S1. All non-hydrogen atoms were refined anisotropically, except those corresponding to disordered atoms. The hydrogen atoms belonging to organic entities have been geometrically fixed and refined according to a riding model with an isotropic thermal parameter linked to the atom to which they are attached (120 %). In most of the cases, the hydrogen atoms of the ligands and anions have been located in the difference Fourier map, while in the coordination water molecules cases the routine CALC-OH[1] implemented in WINGX[2] interface has been employed. The refinement of water hydrogen atoms has been performed using an isotropic thermal parameter of 150% regarding their parent atom. It has not been possible to position geometrically the hydrogen atoms of all crystallization water molecules due to the disorder that some of the present.

During the structural resolution of the compounds, it was common to observe the presence of a static disorder in the adenine molecules with an unusually high thermal motion of some atoms and nearby peaks in the Fourier map differences with high electron density was also observed. This disorder corresponds to the existence of two coplanar positions of the nucleobase with inverted orientation with respect to the coordination mode (μ-κ*N*3:κ*N*9/μ-κ*N*9:κ*N*3). The disorder was modelled including the peaks observed as atoms split in two positions (A and B), to which common free occupancy factors were assigned to each of the subgroups with the condition that the two occupancy factors add up to 100%, Figure S2.

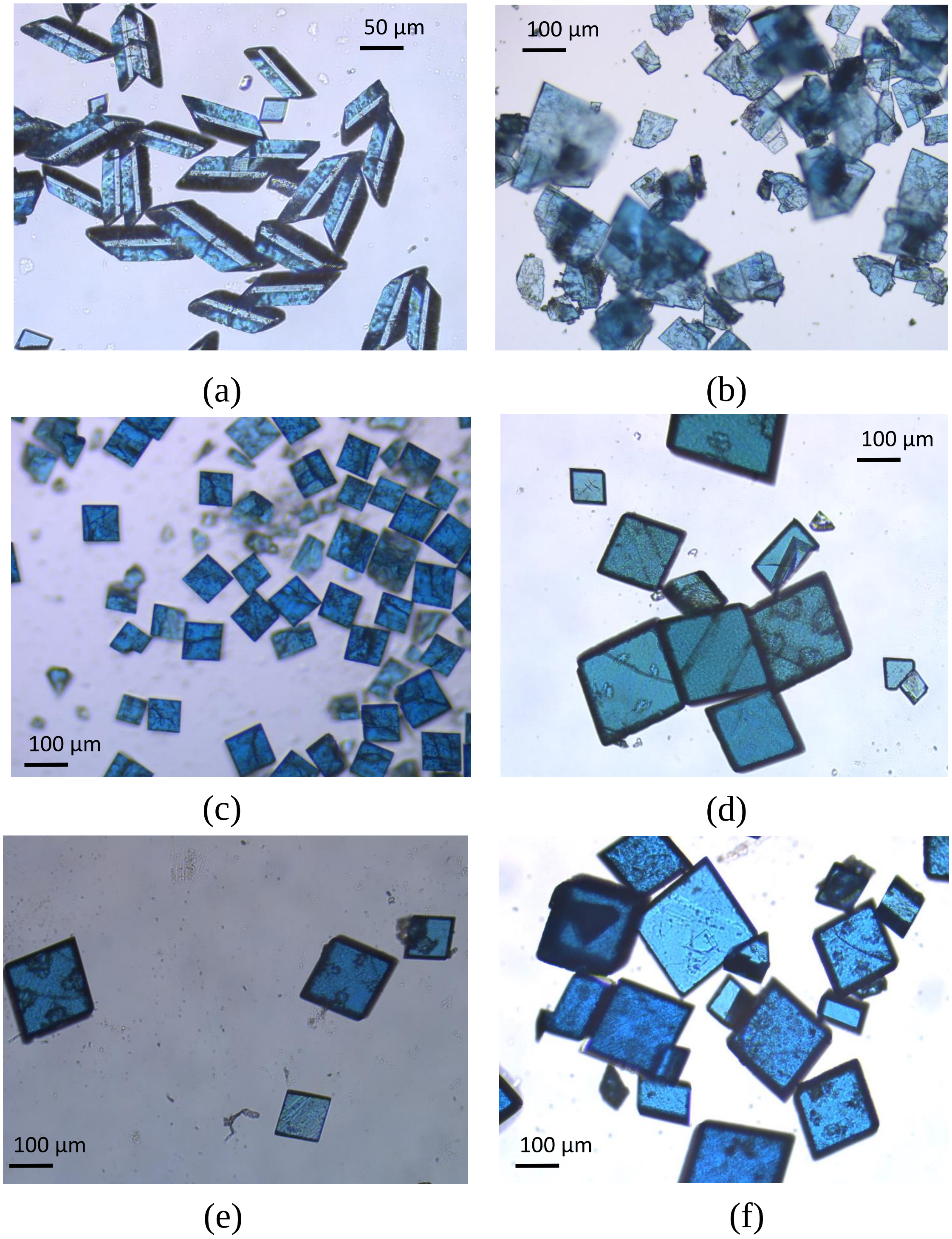

Figure S1. Optical images of the precursor samples: (a) $[Cu_7(\mu\text{-adeninato})_6(\mu_3\text{-OH})_6(\mu\text{-}H_2O)_6]$fumarate (**Cu_F**), (b) $[Cu_7(\mu\text{-adeninato})_6(\mu_3\text{-OH})_6(\mu\text{-}H_2O)_6]$naphthalene-2,6-dicarboxylate (**Cu_N**), (c) $[Cu_7(\mu\text{-adeninato})_6(\mu_3\text{-OH})_6(\mu\text{-}H_2O)_6](\text{benzoate})_2$ (**Cu_B**), (d) $[Cu_6Co(\mu\text{-adeninato})_6(\mu_3\text{-OH})_6(\mu\text{-}H_2O)_6]$fumarate (**CuCo_F**) (e) $[Cu_6Ni(\mu\text{-adeninato})_6(\mu_3\text{-OH})_6(\mu\text{-}H_2O)_6]$fumarate (**CuNi_F**) and (f) $[Cu_6Zn(\mu\text{-adeninato})_6(\mu_3\text{-OH})_6(\mu\text{-}H_2O)_6]$fumarate (**CuZn_F**).

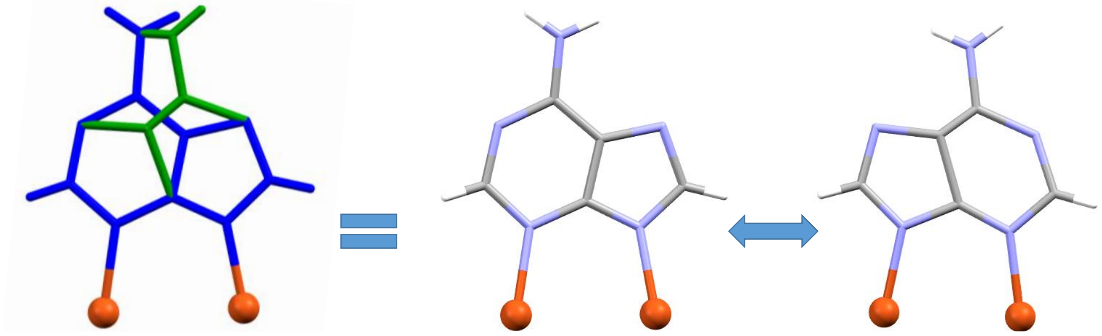

Figure S2. Disorder of the adeninato ligand with two coplanar orientations colored in blue and green.

Single-crystal X-ray Diffraction Data were collected on Agilent Technologies Supernova diffractometers. The data reduction was done with the CrysAlisPro program.[3] Crystal structures were solved by direct methods using the SIR92[4] for **Cu_F**, **Cu_N**, and **Cu_B** from SHELXS[5] programs and refined by full-matrix least-squares on F^2 including all reflections (WINGX). In many of these structures, some of the adeninato ligands appear disordered as well as some of the organic counterions which in some cases required the use of several restraints (DFIX, SADI and FLAT) during the crystal structure refinement.

The crystal structure of all these compounds show the presence of great channels in which the solvent molecules ions are placed highly disordered. It precluded their modeling and, therefore, the electron density at the voids of the crystal structure was subtracted from the reflection data by the SQUEEZE method[6] as implemented in PLATON.[7] Once the process has been carried out, it is verified that the holes generated by the program are susceptible to the presence of a water molecule, taking as an example the value of ten electrons for each molecule. The representation of the crystal structures has been made by MERCURY.[8]

Table S1. Crystallographic data and structure refinement details of compounds.[a,b]

	**Cu_F**	**Cu_N**	**CuCo_F**	**CuNi_F**	**CuZn_F**
empirical formula	$C_{34}H_{80}N_{30}O_{34}Cu_7$	$C_{42}H_{112}N_{30}O_{48}Cu_7$	$C_{28}H_{42}CoCu_6N_{24}O_{10}$	$C_{34}H_{82}Cu_6NiN_{30}O_{35}$	$C_{34}H_{92}N_{30}O_{40}Cu_6Zn$
formula weight	1898.06	2250.39	2001.54	1911.24	2007.98
crystal system	Monoclinic	Orthorhombic	Monoclinic	Monoclinic	Monoclinic
space group	*C*2/*c* (15)	*Pbcn* (60)	*C*2/*c* (15)	*C*2/*c* (15)	*C*2/*c* (15)
a	52.134(3)	16.0999(4)	36.179(3)	36.245(4)	36.207(4)
b	16.7578(6)	17.8124(7)	16.4648(4)	16.4286(5)	16.4605(4)
c	19.9028(10)	32.9353(9)	18.0085(15)	17.8649(19)	17.9932(19)
α	90	90	90	90	90
β	122.401(7)	90	133.431(15)	135.19(2)	133.753(18)
γ	90	90	90	90	90
V ($Å^3$)	14681.1(15)	9445.1(5)	7790(2)	7498(2)	7746(2)
Z	8	4	4	4	4
T (K)	170.01(10)	150.00(10)	170.0(1)	170.0(1)	170.01(10)
λ (Å)	0.71073	1.54184	0.71073	0.71073	0.71073
Sizes (mm)	0.06, 0.02, 0.01	0.08, 0.07, 0.03	0.08, 0.08, 0.02	0.07, 0.07, 0.04	0.06, 0.03, 0.03
Shape	Plate	Square	Cubic	Cubic	Square
Color	Blue	Blue	Green	Blue	Blue
θ interval	1.845 – 24.200	3.701 – 62.996°	2.094 – 29.961	2.094 – 24.998	2.093 – 24.998
hkl interval	–60≤h≤60;<br>–19≤k≤18;<br>–22≤l≤22	–18≤h≤14;<br>–20≤k≤20;<br>–38≤l≤38	–42≤h≤42;<br>–19≤k≤19<br>–21≤l≤21	–42≤h≤43;<br>–19≤k≤19;<br>–21≤l≤21	–42≤h≤42;<br>–19≤k≤17;<br>–21≤l≤21;
ρ_c ($g \cdot cm^{-3}$)	1.717	1.583	1.707	1.707	1.722
μ (cm^{-1})	2.091	2.622	1.922	1.922	2.028
F(000)	7752	4644	4108	4108	4120
S^a	1.212	1.093	1.076	1.700	1.108
R_{int}	0.0691	0.0821	0.0354	0.0311	0.0371
Parameters	734	456	412	377	452
Weight scheme[c]	Shelx	Shelx	Shelx	Shelx	Shelx
final R indices					
[I > 2σ(I)] $R_1{}^b/wR_2{}^c$	0.1061/0.3167	0.1334/0.3931	0.0943/0.2834	0.1385/0.4153	0.0918/0.2733
all data $R_1{}^b/wR_2{}^c$	0.1562/0.3521	0.1621/0.4204	0.1123/0.3022	0.1696/0.4435	0.1153/0.2944

[a]$S=[\sum w(F_0^2-F_c^2)^2/(N_{obs}-N_{param})]^{1/2}$. [b]$R_1=\sum||F_0|-|F_c||/\sum|F_0|$. [c]$wR_2 = [\sum w(F_0^2-F_c^2)^2/\sum wF_0^2]^{1/2}$; $w = 1/[\sigma^2(F_0^2) + (aP)^2 + b]$ where $P = (\max(F_0^2,0) + 2F_c^2)/3$; **Cu_F** (a = 0.2000, b = 0); **Cu_N** (a = 0.1997, b = 40.7961), **CuCo_F** (a = 0.1806, b = 48.9128), **CuNi_F** (a = 0.2000, b = 0) and **CuZn_F** (a = 0.1636, b = 35.9140).

Note: Structural and chemical characterization of compound **Cu_B** has been previously reported and can be found in reference 27 of the manuscript.

The crystal structure of all the precursors consist of cationic wheel-shaped [$Cu_6M(\mu$-adeninato)$_6(\mu_3$-OH)$_6(\mu$-$H_2O)_6]^{2+}$ (M^{II}: Cu, Co, Ni and Zn) entities, in which the $[M(OH)_6]^{4-}$ core is connected to the six copper(II) metal centers comprising the external ring, Figure S3. All the copper(II) metal centers present an octahedral geometry with the usual Jahn-Teller tetragonal elongation which is more pronounced for the external copper(II) atoms than for the inner one because of the rigidity of the heptanuclear entity (Table S2). The peripheral copper(II) atoms are further connected among them through the double μ-H_2O and μ-ade-κ*N3*:κ*N9* bridge. The 2+ charge of these heptameric entities is counterbalanced by fumarate anions, Figure S4.

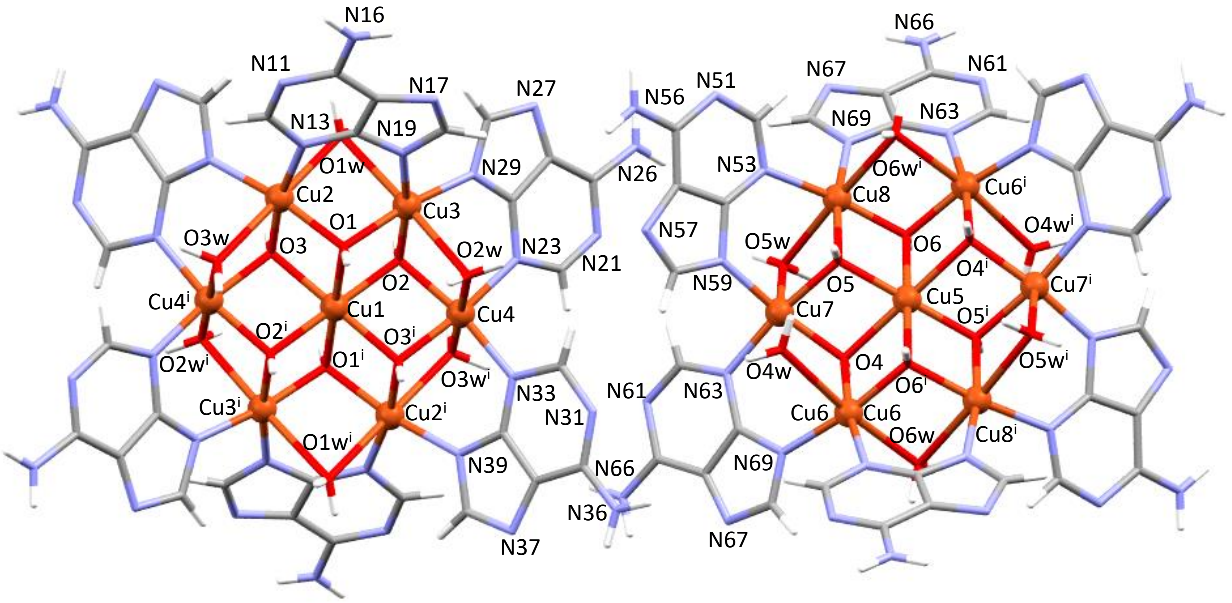

Figure S3. The two crystallographically independent heptameric entities found in compound **Cu_F** with the corresponding atom labelling scheme.

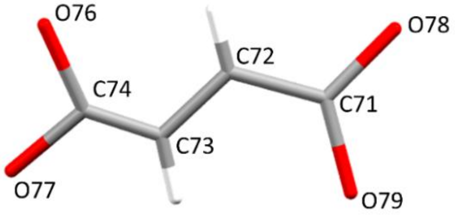

Figure S4. Atom labelling scheme for fumarate anion in compound **Cu_F**.

The heptameric entities approach each other in such a way that they are able to establish π–π interactions through offset face-to-face π–π stacks between adeninato ligands (Figure S5). Each unit is connected to the adjacent ones by a double π–π stacking interaction between adeninato ligands. This stacking interactions are reinforced by a hydrogen-bond ring formed by a

double N–H···N interaction between the pyrimidinic exocyclic N66 nitrogen atoms of the previously no interacting adeninato ligand and the N17 of an adjacent adeninato as acceptor.

The fumarate dicarboxylate anions are inserted in between adeninato ligands through a A···A···F···A···A π–stacking interaction along the crystallographic c-axis. The previous supramolecular interaction is reinforced through hydrogen-bonding interactions. One carboxylate group of the organic anion is attached to the nearest cationic layer by two O_{anion}···H–O_{cation} hydrogen bonds, one with a hydroxide group and the other one with a water molecule coordinated to the same copper atom, to form a supramolecular $R_2^2(8)$ synthon, but the other one is attached to another cluster by a coordination water molecule and to a crystallization one located in the void (Figure S6, Table S3).

Table S2. Distances and angles (Å, deg) for compound **Cu_F.**[*a*]

Cu1–O1	2.251(9)	Cu1–O2	1.978(9)	Cu1–O3	2.010(9)
Cu2–O1	1.976(9)	Cu2–O3	2.028(9)	Cu2–13	2.018(13)
Cu2–N39^{i}	1.984(15)	Cu2–O1w	2.549(10)	Cu2–O3w	2.470(10)
Cu3–O1	1.966(10)	Cu3–O2	1.999(9)	Cu3–N19	1.958(13)
Cu3–N29	1.952(13)	Cu3–O1w	2.568(10)	Cu3–O2w	2.450(10)
Cu4–O2	1.966(9)	Cu4–O3^{i}	1.944(10)	Cu4–N23	1.961(13)
Cu4–N33	2.007(16)	Cu4–O2w	2.454(10)	Cu4–O3w$^{\mathbf{i}}$	2.483(10)
Cu5–O4	2.171(10)	Cu5–O5	1.929(8)	Cu5–O6	2.116(8)
Cu6–O4	1.972(9)	Cu6–O4w	2.750(11)	Cu6–O6w	2.427(11)
Cu6–N49	1.961(12)	Cu6–O6^{ii}	1.953(10)	Cu6–N63^{ii}	1.997(10)
Cu7–O4	1.941(9)	Cu7–O5	1.987(9)	Cu7–N43	1.999(12)
Cu7–N59	1.935(12)	Cu7–O4w	2.862(12)	Cu7–O5w	2.359(9)
Cu8–O5	1.995(7)	Cu8–O6	1.949(9)	Cu8–N53	2.028(12)
Cu8–N69	1.957(12)	Cu8–O5w	2.483(10)	Cu8–O6w^{ii}	2.474(12)
Cu1···Cu2	3.182(2)	Cu1···Cu3	3.135(2)	Cu1···Cu4	3.023(2)
Cu2···Cu3	3.071(3)	Cu2···Cu4^{i}	3.134(3)	Cu3···Cu4	3.136(3)
Cu5···Cu6	3.161(2)	Cu5···Cu7	3.077(2)	Cu5···Cu8	3.073(2)
Cu6···Cu7	3.109(2)	Cu6···Cu8^{ii}	3.086(3)	Cu7···Cu8	3.120(2)
Cu1–O1–Cu2	97.5(4)	Cu1–O1–Cu3	95.8(4)	Cu2–O1–Cu3	102.4(4)
Cu1–O2–Cu3	104.1(4)	Cu1–O2–Cu4	100.1(3)	Cu3–O2–Cu4	104.6(4)
Cu1–O3–Cu2	104.0(4)	Cu1 –O3–Cu4^{i}	99.7(4)	Cu2–O3–Cu4^{i}	104.2(4)
Cu2–O1w–Cu3	73.8(2)	Cu3 –O2w–Cu4	79.5(3)	Cu2–O3w–Cu4^{i}	78.5(3)
Cu5–O4–Cu6	99.4(4)	Cu5–O4–Cu7	96.7(4)	Cu5–O5–Cu7	103.6(3)
Cu5–O5–Cu8	103.1(4)	Cu5–O6–Cu6^{ii}	101.9(4)	Cu6–O4–Cu7	105.3(4)
Cu7–O5–Cu8	103.2(4)	Cu5–O6–Cu8	98.2(4)	Cu8 –O6–Cu6^{ii}	104.5(3)
Cu6–O4w–Cu7	67.3(3)	Cu7–O5w–Cu8	80.2(3)	Cu6–O6w–Cu8^{ii}	78.1(3)

[a] Symmetry: (i) – x, – y, 1 – z, (ii) 1/2 – x, 1/2 – y, 1 – z.

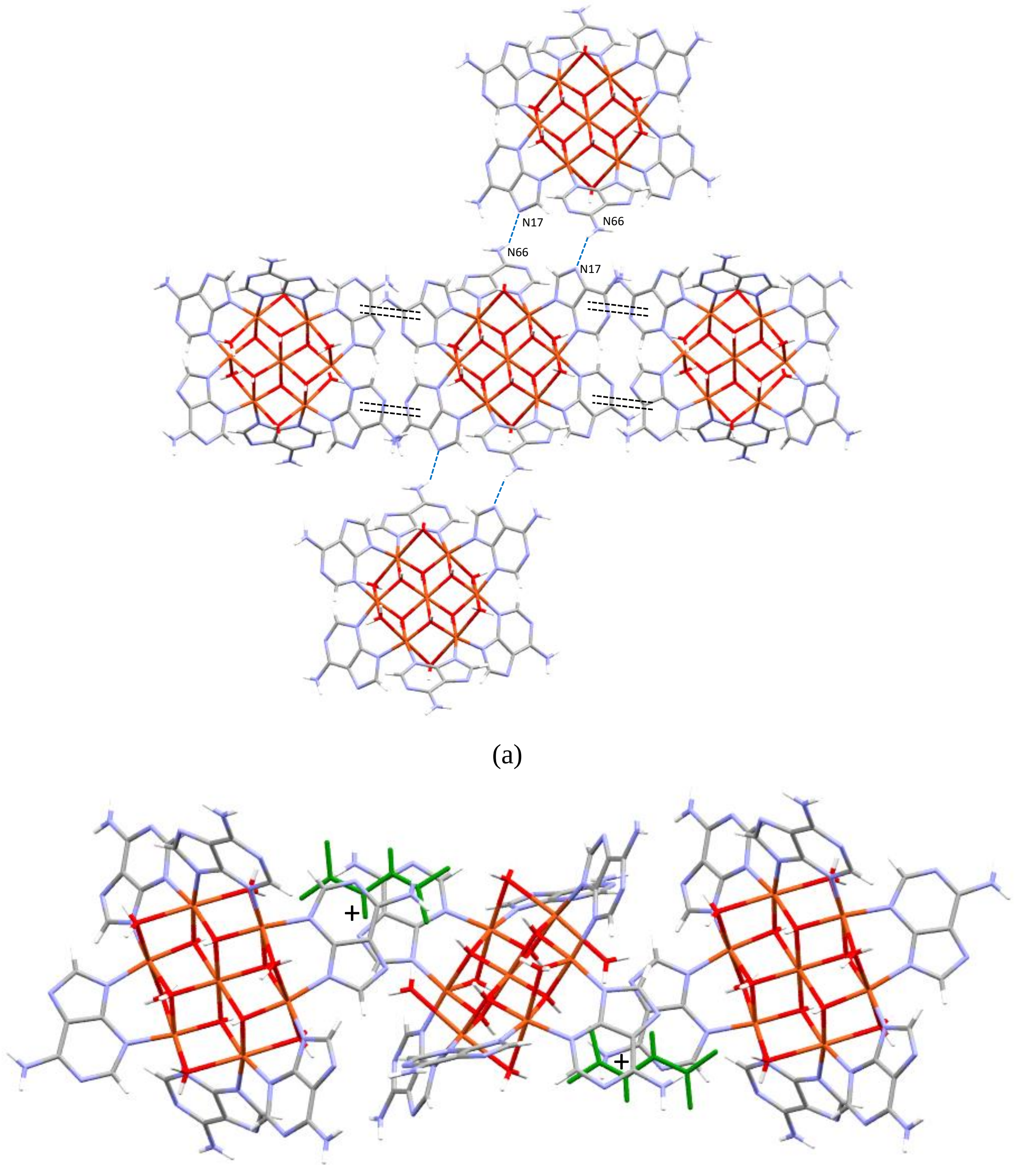

(a)

(b)

Figure S5. Crystal packing fragments of compound **Cu_F** (double dashed lines/+: π−π stacking, and blue dashed line: hydrogen-bonding interactions)

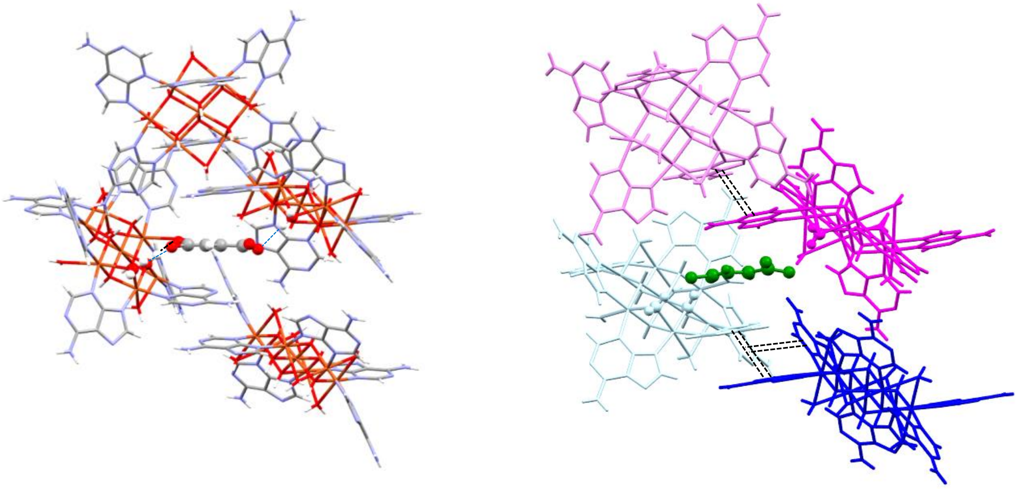

(a) (b)

Figure S6. Supramolecular interactions of the fumarate anion in compound **Cu_F** highlighting the hydrogen bonding interactions (a), and the π-stacking interactions (b).

Table S3. Structural parameters (Å, deg) of supramolecular interactions in **Cu_F**.[a]

Hydrogen-bonding interactions

D−H···A[b]	H···A	D···A	D−H···A
O5−H5···O78	1.69	2.67(2)	164
O3w−H31w···O76^{i}	2.20	3.02(2)	171
O4w−H31w···O78	1.96	2.77(2)	163
O6w −H62w···O79^{ii}	2.05	2.87(2)	165
N16−H16B···N37^{iii}	2.22	3.01(2)	149
N66−H66A···N17^{iv}	2.34	3.17(2)	156

***π–π* interactions** [c]

ring–ring	packing	angle	DC	DZ	DXY
2pa····5pa^{v}	A····A	3.0	4.76	3.28	3.39
3pa····4pa^{v}	A····A	7.0	5.99	3.23	4.68

[a]Symmetry codes: (i) − x, y, 1/2 − z; (ii) 1/2 − x, 1/2 – y, 1 – z; (iii) x, −1 + y, z; (iv) 1/2 – x, 1/2 + y, 3/2 – z; (v) x, y, z;. [b]D: donor; A: acceptor. [c]Angle: dihedral angle between the planes (deg), DC: distance between the centroids of the rings (Å), DZ: interplanar distance (Å), DXY: lateral displacement (Å), pa: adenine pentagonal ring, ha: adenine hexagonal ring.

Similarly to **Cu_F** compound, **Cu_N** is also based on heptameric entities (Table S4) counterbalanced, in this case, by naphthalene-2,6-dicarboxylate anions. Cationic layers sustained by π–π adeninato⋯adeninato stacking of four adjacent adeninato entities are also present in compound **Cu_N**, but their pendant adeninato ligands interact with each other through a double N–H⋯N hydrogen bond involving the Hoogsteen face of the nucleobase, Figure S7, Table S5. Dicarboxylate anion is parallel stacked to both ligands and the hydrogen bonding rings $R_2^2(8)$ between its carboxylate groups and the O–H sites of the heptameric entity are still present. These supramolecular interactions also generate supramolecular boxes, with an inner cavity filled by solvation water molecules to give a **pcu** topology.

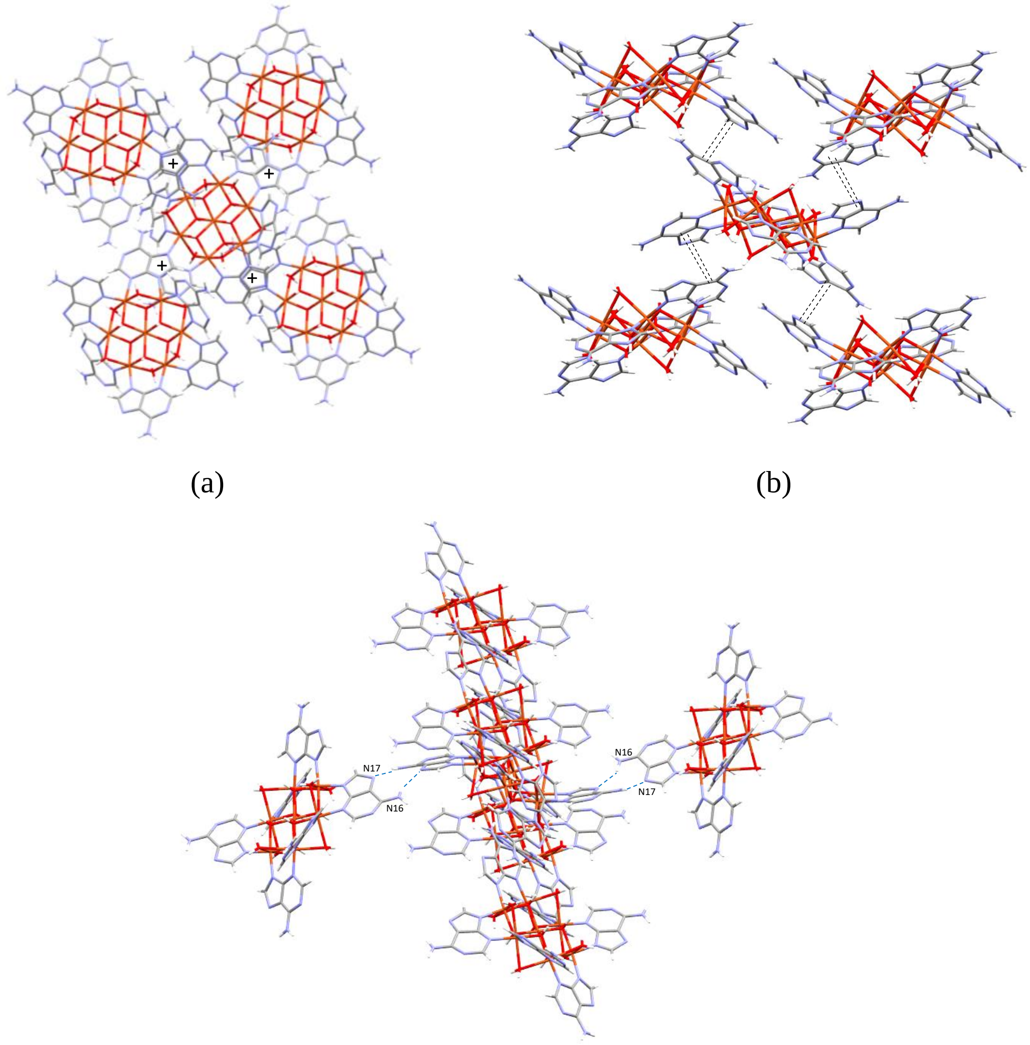

(a) (b)

(c)

Figure S7. (a, b) Supramolecular interaction sustaining the porous architecture and (c) adeninato⋯adeninato π–stacking interactions in compound **Cu_N**.

The carboxylate groups of the naphthalene-2,6-dicarboxylate anion are attached to the nearest cationic layer by two $O_{anion}\cdots H–O_{cation}$ hydrogen bonds, one with a hydroxide group and the other one with a water molecule coordinated to the same copper atom, to form a supramolecular $R_2^2(8)$ synthon. Additionally, the aromatic rings establish offset face-to-face $\pi–\pi$ interactions with both adeninato moieties; Figure S8.

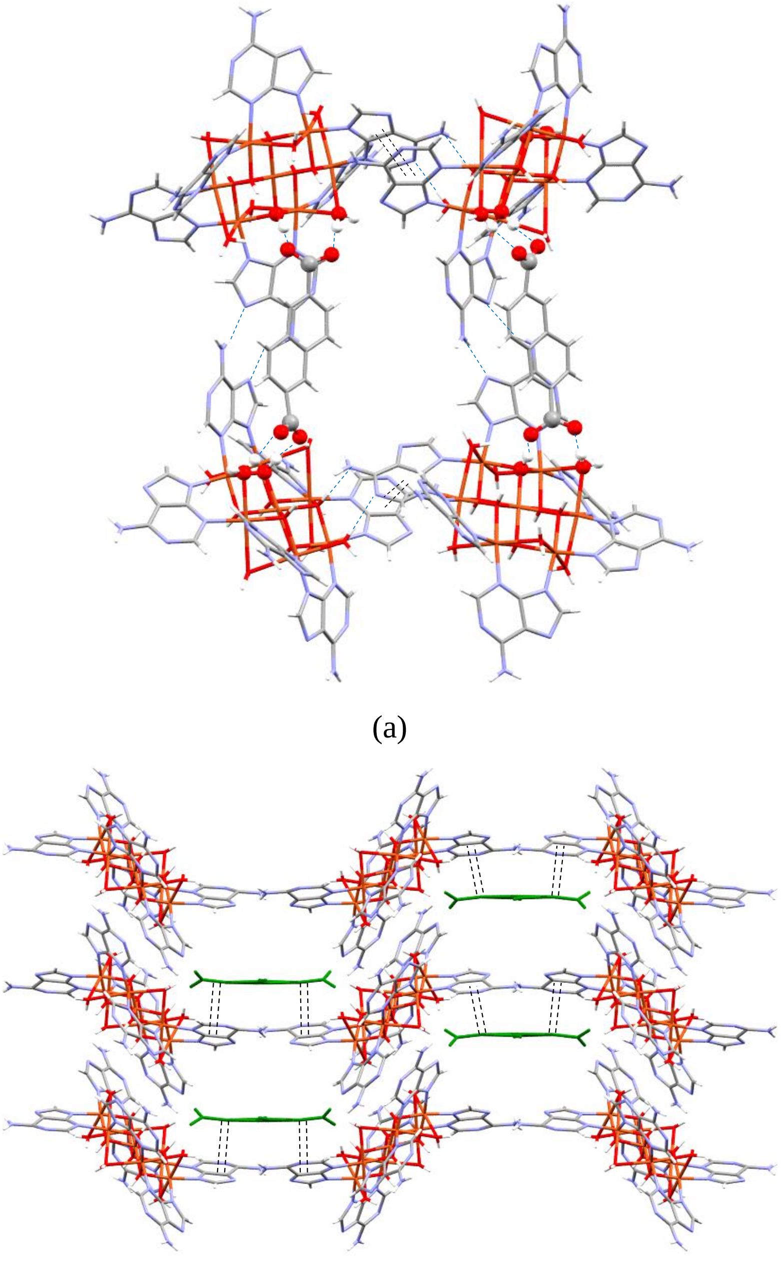

(a)

(b)

Figure S8. (a and b) Details of the supramolecular interactions between the organic anion (green colour) and the cationic complexes in compound **Cu_N**.

Table S4. Distances and angles (Å, deg) for compound **Cu_N**.[a]

Cu1–O1	2.099(8)	Cu1–O2	2.126(7)	Cu1–O3	1.972(6)
Cu2–O1	1.966(7)	Cu2–O3^{i}	1.965(7)	Cu2–N13	2.004(1)
Cu2–N29	2.015(1)	Cu2–O1w	2.423(9)	Cu2–O3w^{i}	2.494(9)
Cu3–O1	1.957(9)	Cu3–O2	1.978(7)	Cu3–N23	2.042(1)
Cu3–N33	1.949(1)	Cu3–O1w	2.377(7)	Cu3–O2w	2.677(1)
Cu4–O2	1.969(6)	Cu4–O3	2.022(7)	Cu4–N19^{i}	1.979(1)
Cu4–N39	2.014(12)	Cu4–O2w	2.556(1)	Cu4–O3w	2.442(8)
Cu1···Cu2	3.101(2)	Cu1···Cu3	3.109(2)	Cu2···Cu4	3.096(2)
Cu2···Cu3	3.056(2)	Cu2···Cu4^{i}	3.117(2)	Cu3···Cu4	3.133(2)
Cu1–O1–Cu2	99.4(3)	Cu1–O1–Cu3	100.0(4)	Cu2–O1–Cu3	102.3(4)
Cu1–O2–Cu3	98.5(3)	Cu1–O2–Cu4	98.2(3)	Cu3–O2–Cu4	105.1(3)
Cu1–O3–Cu2^{i}	104.0(3)	Cu1–O3–Cu4	101.6(3)	Cu4–O3–Cu2^{i}	102.8(3)
Cu2–O1w–Cu4	79.1(2)	Cu3–O2w–Cu4	73.5(3)	Cu4–O3w–Cu2^{i}	78.3(3)

[a] Symmetry: (i) 2 –x, –y, 1 –z.

Each carboxylate group of the organic anion is attached to the nearest cationic layer by two O_{anion}···H–O_{cation} hydrogen bonds, one with a hydroxide group and the other one with a water molecule coordinated to the same copper atom, to form a supramolecular $R_2^2(8)$ synthon. Additionally, the aromatic rings naphthalene–2,6-dicarboxylate anions establish offset face-to-face π–π interactions with both adeninato moieties (Figure S9, Table S5).

The above-described supramolecular interactions generate rectangular structural boxes (Figure S9), where the crystallization water molecules are hosted, resembling the reticular topology of the well-known IRMOF metal-organic framework. Heptameric units are located at the eight vertices of these boxes. The edges of the upper and lower faces of these boxes are defined by the adeninato···adeninato π–stacking interactions with no left empty space, whereas the lateral edges imply the adenine sandwiched organic anions. The distance between the heptameric clusters in the filled faces is *ca.* 12.1 Å, with a longitudinal distance of 14.9 Å.

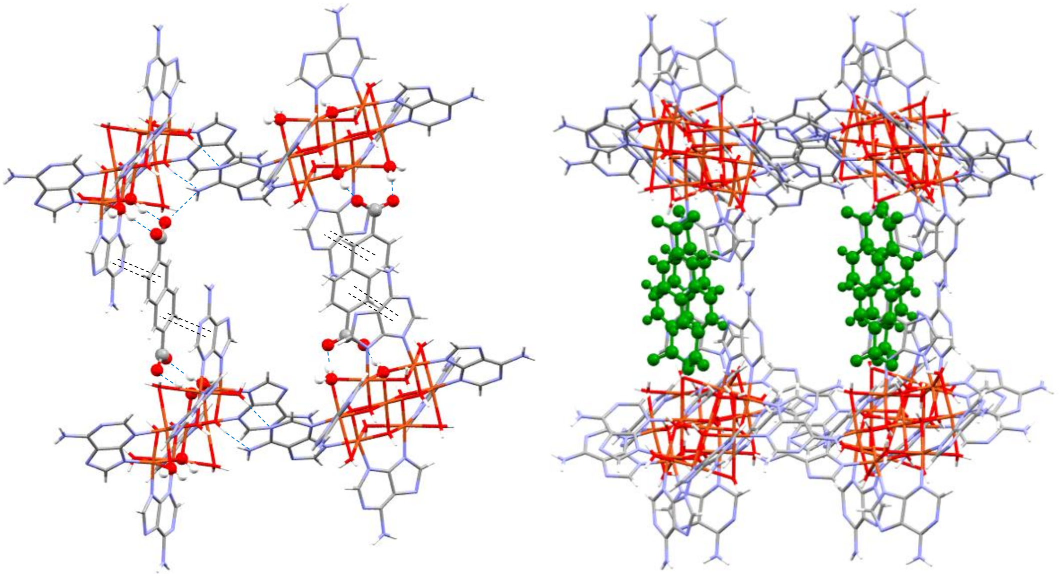

Figure S9. Supramolecular box in the crystal structure of compound **Cu_N**.

Table S5. Structural parameters (Å, deg) of hydrogen bonding interactions in compound **Cu_N**.[a]

**Hydrogen-bonding interactions**			
D–H···A[b]	H···A	D···A	D–H···A
O1–H1···N36^{i}	2.00	2.85(2)	173
O2–H2···O5w^{ii}	1.84	2.81(1)	170
O3–H3···O48^{iii}	1.67	2.63(1)	164
O1W–H11W···049	1.88	2.72(1)	175
O1W–H12W···O4w	1.88	2.77(1)	177
O2W–H22W···O48^{iii}	2.17	2.98(2)	171
O3W–H31W···O8w^{iv}	1.99	2.86(1)	172
O3W–H32W···N31^{iv}	2.02	2.86(2)	177
N16A–H16A···N17^{v}	2.30	3.11(2)	156
N26A–H16A···O4w^{vi}	1.98	2.84(2)	171
N36–H36A···O5w^{vi}	2.31	3.03(2)	141

***π–π* interactions** [c]					
ring – ring[a]	packing	angle	DC	DZ	DXY
1pa···3pa^{vii}	A····A	6.4	4.28	3.65	2.34
2pa···1hd^{viii}	A····A	4.0	3.86	3.60	1.65
2pa···2hd^{ix}	A····A	4.0	3.86	5.49	1.42

[a]Symmetry codes: (i) 3/2 – x, – 1/2 + y, z; (ii) 3/2 – x, 1/2 + y, z; (iii) 2 – x, – y, 1 – z; (iv) 1/2 + x, 1/2 – y, 1 – z, (v) 2 – x, y, 1/2 – z, (vi) 1 – x, – y, 1 – z, (vii) 2 – x, 1/2 + y, 5/2 –z, (viii) x, y, z, (ix) 3 – x, – y, 2 – z. [b]D: donor; A: acceptor. [c]Angle: dihedral angle between the planes (deg), DC: distance between the centroids of the rings (Å), DZ: interplanar distance (Å), DXY: lateral displacement (Å), pa: adenine pentagonal ring, ha: adenine hexagonal ring,.hd: anion hexagonal ring.

In the heterometallic compounds **CuCo_F**, **CuNi_F** and **CuZn_F** the heptameric entities establish again adeninato···adeninato π–π stacking interactions involving four of the adeninato ligands in such a way that each heptamer is connected to four adjacent ones creating supramolecular sheets, Figure S10, Table S6–S8. These connections between heptameric units are reinforced by the presence of an additional double hydrogen bond involving the hexagonal ring (NX1) and the amino group (NX6-H) of the adeninato ligands from adjacent heptameric entities and also the coordinated hydroxides and water molecules.

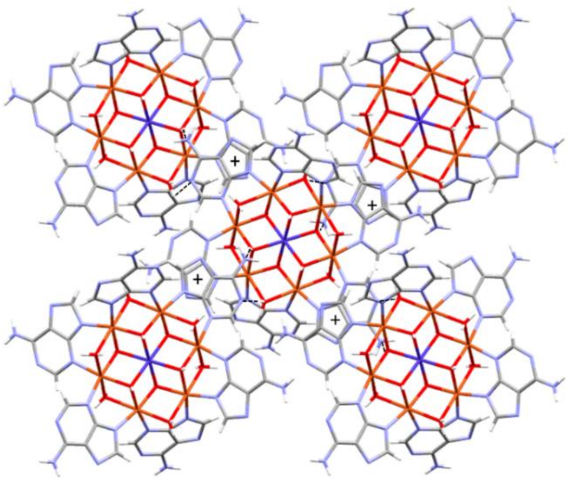

(a)

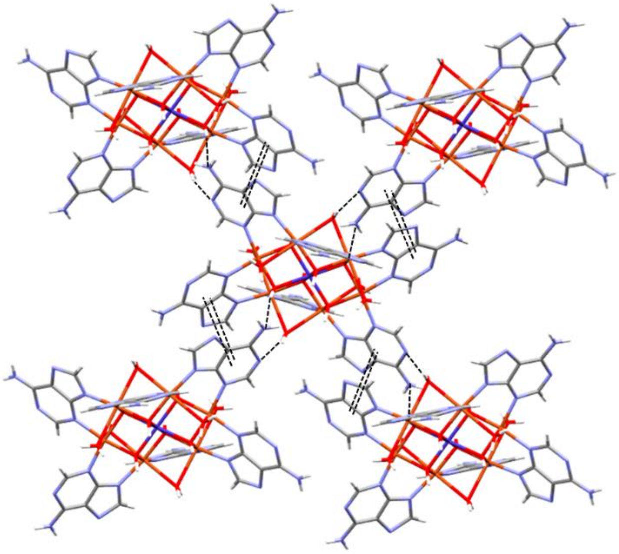

(b)

Figure S10. Crystal packing of heterometallic compound **CuZn_F** sustained by supramolecular interactions connecting the structural entities through a) π–π interactions: double dashed lines or + symbol and b) hydrogen bonds: dotted lines.

The supramolecular architecture is completed by direct hydrogen bonding interactions (Table S9–S11) between the carboxylate groups of the fumarate anion located on the two adeninato ligands that do not take part in the above interactions and a hydroxide and a coordination water molecule cluster of the original and another heptamer, Figure S11.

However, the fumarate anion established supramolecular interactions in heterometallic compounds differ from those established in the homometallic compounds **Cu_F** although in both cases it is able to connect the previously described supramolecular sheets. The fumarate anion is again arranged between two adeninato ligands from adjacent heptameric entities, but now, it is arranged perpendicularly instead of parallel with respect to planar adeninato ligands, Figure S11. In addition to that, the carboxylate groups of the fumarate anion also establish hydrogen bonding interactions with the hydroxide and coordinated water molecules of the heptameric entities, Figure S12.

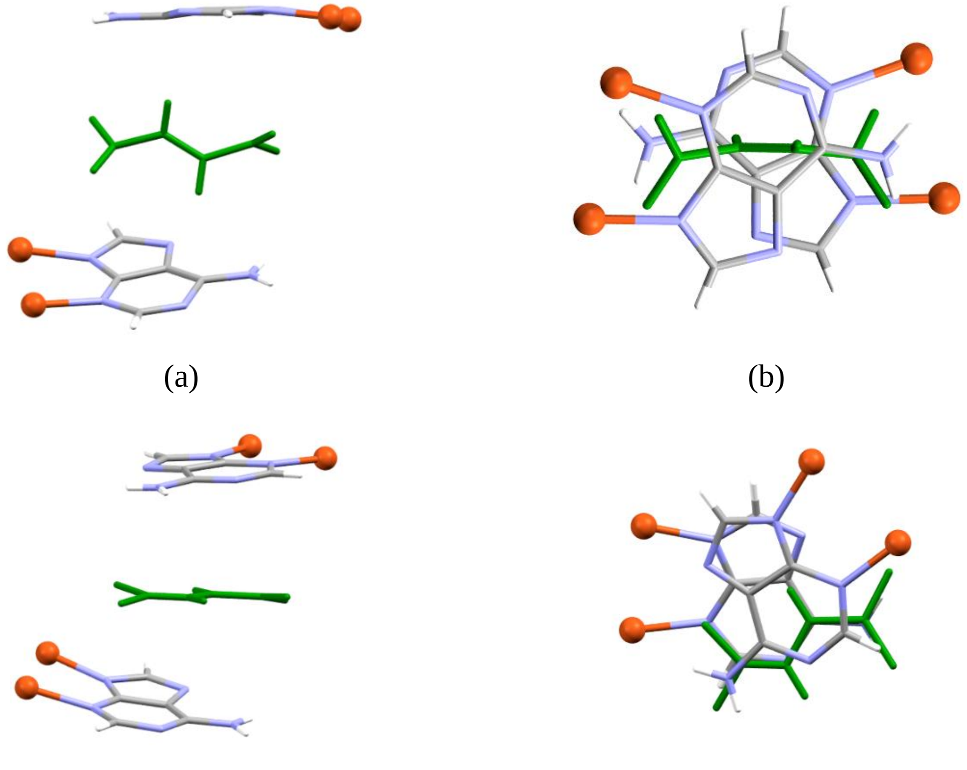

(a) (b)

(c) (d)

Figure S11. Representation of the fumarate anion insertion between two adjacent adeninato ligands in **CuZn_F** (a-b), and in **Cu_F** (c-d).

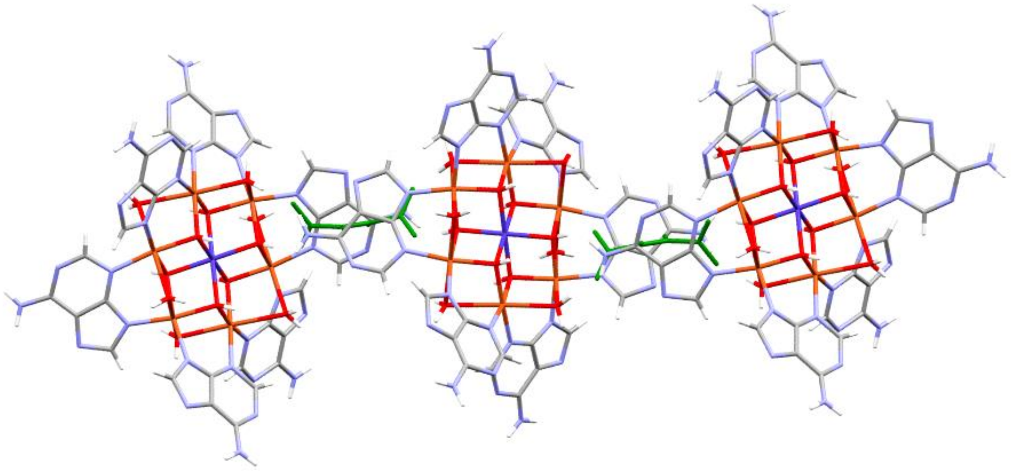

(a)

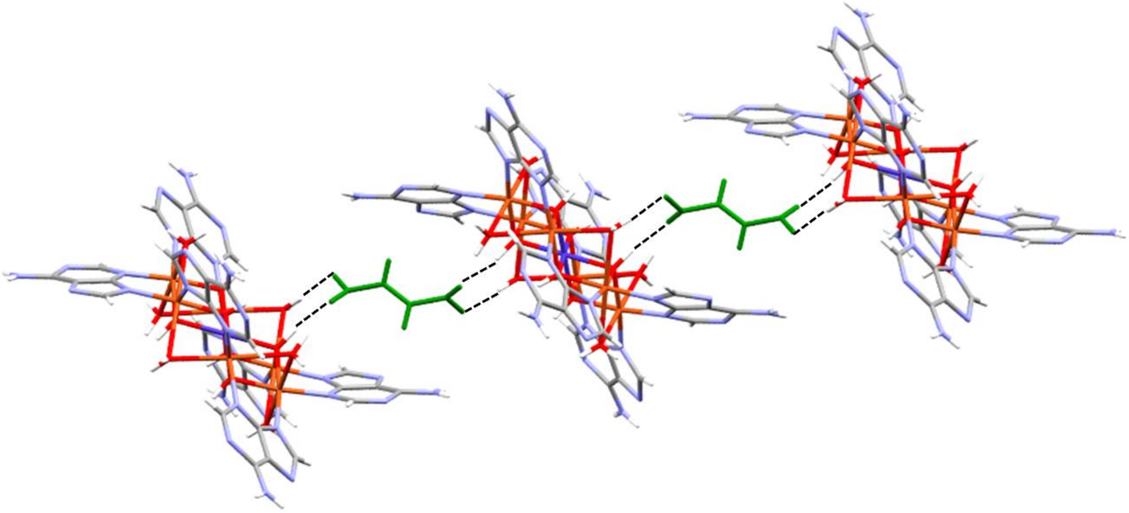

(b)

Figure S12. (a) Insertion of the fumarate anion between the heptameric entities and (b) the hydrogen bonding interactions established by it.

Table S6. Distances and angles (Å, deg) for compound **CuCo_F**.[a]

Co1–O1	2.008(4)	Co1–O2	2.074(5)	Co1–O3	2.075(6)
Cu2–O1	1.982(5)	Cu2–O3	1.991(4)	Cu2–N13	2.032(8)
Cu2–N33[i]	2.030(1)	Cu2–O1w	2.369(9)	Cu2–O3w[i]	2.495(1)
Cu3–O1	1.967(5)	Cu3–O2	1.976(5)	Cu3–N19	1.986(8)
Cu3–N29	1.957(1)	Cu3–O1w	2.485(9)	Cu3–O2w	2.474(7)
Cu4–O2	1.984(5)	Cu4–O3[i]	1.964(6)	Cu4–N23	1.995(1)
Cu4–N39	2.023(1)	Cu4–O2w	2.464(7)	Cu4–O3w	2.519(9)
Co1···Cu2	3.101(1)	Cu1···Cu3	3.130(1)	Cu1···Cu4	3.109(1)
Cu2···Cu3	3.101(2)	Cu2···Cu4[i]	3.126(2)	Cu3···Cu4	3.114(1)
Co1–O1–Cu2	99.2(2)	Co1–O1–Cu3	101.1(2)	Cu2–O1–Cu3	103.5(3)
Co1–O2–Cu3	101.2(3)	Co1–O2–Cu4	100.0(3)	Cu3–O2–Cu4	103.7(3)
Co1–O3–Cu2	99.4(3)	Co1–O3–Cu4[i]	100.6(3)	Cu2–O3–Cu4[i]	104.4(3)
Cu2–O1w–Cu3	79.4(3)	Cu3–O2w–Cu4	78.2(2)	Cu2i–O3w–Cu4	77.1(3)

[a] Symmetry: (i) 3/2 – x, 1/2 – y, 1 – z.

Table S7. Distances and angles (Å, deg) for compound **CuNi_F**.[a]

Ni1–O1	2.063(9)	Ni1–O2	1.994(10)	Ni1–O3	2.072(7)
Cu2–O1	1.974(8)	Cu2–O3[i]	1.957(9)	Cu2–N13	1.980(2)
Cu2–N33[i]	2.022(1)	Cu2–O1w	2.600(2)	Cu2–O3w[i]	2.405(2)
Cu3–O1	1.953(1)	Cu3–O2	2.008(1)	Cu3–N19	2.044(2)
Cu3–N23	2.031(2)	Cu3–O1w	2.490(2)	Cu3–O2w	2.446(1)
Cu4– O2	1.978(9)	Cu4–O3	1.942(1)	Cu4–N29	1.890(3)
Cu4–N39	2.042(2)	Cu4–O2w	2.476(1)	Cu4–O3w	2.485(2)
Ni1···Cu2	3.087(1)	Ni1···Cu3	3.090(2)	Ni1···Cu4	3.080(2)
Cu2···Cu3	3.093(2)	Ni2···Cu4[i]	3.072(3)	Cu3···Cu4	3.093(2)
Ni1–O1–Cu2	99.7(4)	Ni1–O1–Cu3	100.6(5)	Cu2–O1–Cu3	103.9(5)
Ni1–O2–Cu3	101.1(5)	Ni1–O2–Cu4	101.7(5)	Cu3–O2–Cu4	101.8(5)
Ni1–O3–Cu2[i]	100.0(4)	Ni1–O3–Cu4[i]	100.2(4)	Cu2[i] –O3–Cu4	104.0(5)
Cu2–O1w–Cu3	74.8(7)	Cu3–O2w–Cu4	77.9(4)	Cu2[i]–O3w–Cu4	77.8(6)

[a] Symmetry: (i) 3/2 – x, 1/2 – y, 1 – z.

Table S8. Distances and angles (Å, deg) for compound **CuZn_F**.[a]

Zn1–O1	2.038(5)	Zn1–O2	2.062(5)	Zn1–O3	2.048(5)
Cu2–O1	1.998(5)	Cu2–O3^{i}	1.960(6)	Cu2–N13	1.984(10)
Cu2–N39^{i}	2.024(10)	Cu2–O1w	2.493(6)	Cu2–O3w^{i}	2.505(7)
Cu3–O1	1.971(5)	Cu3–O2	1.976(5)	Cu3–N19	1.952(10)
Cu3–N29	1.993(9)	Cu3–O1w	2.459(7)	Cu3–O2w	2.475(7)
Cu4–O2	1.960(5)	Cu4–O3	1.978(5)	Cu4–N23	2.016(9)
Cu4–N33	2.015(10)	Cu4–O2w	2.359(8)	Cu4–O3w	2.493(8)
Zn1···Cu2	3.0853(14)	Zn1···Cu3	3.1055(12)	Zn1···Cu4	3.0835(9)
Cu2···Cu3	3.1013(16)	Cu2···Cu4^{i}	3.0963(18)	Cu3···Cu4	3.078(2)
Zn1–O1–Cu2	99.7(2)	Zn1–O1–Cu3	101.5(2)	Cu2–O1–Cu3	102.8(2)
Zn1–O2–Cu3	100.5(2)	Zn1–O2–Cu4	100.1(2)	Cu3–O2–Cu4	102.9(2)
Zn1–O3–Cu2^{i}	100.6(2)	Zn1–O3–Cu4	100.0(2)	Cu2^{i}–O3–Cu4	103.7(2)
Cu2–O1w–Cu3	77.56(18)	Cu3–O2w–Cu4	79.06(19)	Cu2^{i}–O3w–Cu4	76.5(2)

[a] Symmetry: (i) 3/2 – x, 1/2 – y, 1 – z.

Table S9. Structural parameters (Å, °) of the supramolecular interactions in **CuCo_F**.[a]

**Hydrogen-bonding interactions**			
D–H···A[b]	H···A	D···A	D–H···A
O1–H1···N36^{i}	2.12	2.93(2)	157
O2–H2···O49	1.83	2.81(2)	170
O3–H3···O4w	1.84	2.81(2)	170
O1w–H11w···O48	1.79	2.65(2)	176
O1w–H12w···O6w	1.97	2.82(1)	175
O2w–H21w···N31^{i}	1.78	2.61(3)	164
O2w–H22w···O7w	1.94	2.78(1)	171
O3w–H31w···O11w^{ii}	1.77	2.62(3)	168
O3w–H32w···O8w	1.79	2.66(3)	165
N26A–H26B···O11w	2.07	2.89(2)	174

***π–π* interactions** [c]					
ring – ring	packing	angle	DC	DZ	DXY
1pa···3pa^{i}	A····A	4.0	4.06	3.62	1.91

**X–H···*π* interactions**[d]	packing	H–Perp	H···Cg	X–H···Cg	X–H···ring
C42–H42···2pa^{iii}	F····A	2.64	2.81	143	61

[a]Symmetry codes: (i) 3/2 – x, – 1/2 + y, 3/2 – z; (ii) 1/2 + x, 1/2 + y, 1 + z; (iii) 2 – x, y , 1/2 – z. [b]D: donor; A: acceptor. [c]Angle: dihedral angle between the planes (deg), DC: distance between the centroids of the rings (Å), DZ: interplanar distance (Å), DXY: lateral displacement (Å), pa: adenine pentagonal ring, ha: adenine hexagonal ring. [d]H–Perp: Perpendicular distance of H to ring plane; H···Cg: distance of H to the centroid of the ring; X–H···Cg: X–H···Cg angle; X–H···ring: angle of the X–H bond with the aromatic ring.

Table S10. Structural parameters (Å, º) of the supramolecular interactions in **CuNi_F**.[a]

**Hydrogen-bonding interactions**			
D–H···A[b]	H···A	D···A	D–H···A
O1–H1···O5w	1.81	2.77(2)	169
O2–H2···O48	1.78	2.75(3)	171
O3–H3···N16A^{i}	1.93	2.91(3)	177
O3–H3···N16B^{i}	2.15	3.06(7)	154
O2w–H21w···N11^{ii}	2.05	2.91(2)	172
O2w–H22w···O4w	1.91	2.72(2)	163
O3w–H31w···O8w^{iii}	1.95	2.78(2)	173
O3w–H32w···O49	1.78	2.61(3)	164
N36A-H36B···O8w^{ii}	2.35	3.21	175

***π–π* interactions**[c]					
ring – ring	packing	angle	DC	DZ	DXY
1pa···3pa^{ii}	A····A	6.0	4.10	3.67	1.78

**X–H···*π* interactions**[d]	packing	H–Perp	H···Cg	X–H···Cg	X–H···ring
C42–H42···2pa^{iv}	F····A	2.54	2.76	143	61

[a]Symmetry codes: (i) 3/2 – x, 1/2 + y, 1/2 – z; (ii) 3/2 –x, – 1/2 + y, 1/2 – z; (iii) x, 1 – y, – 1/2 + z; (iv) 2 – x, y , 1/2 – z. [b]D: donor;A: acceptor. [c]Angle: dihedral angle between the planes (deg), DC: distance between the centroids of the rings (Å), DZ: interplanar distance (Å), DXY: lateral displacement (Å), pa: adenine pentagonal ring, ha: adenine hexagonal ring. [d]H–Perp: Perpendicular distance of H to ring plane; H···Cg: distance of H to the centroid of the ring; X–H···Cg: X–H···Cg angle; X–H···ring: angle of the X–H bond with the aromatic ring.

Table S11. Structural parameters (Å, º) of the supramolecular interactions in compound **CuZn_F**.[a]

**Hydrogen-bonding interactions**			
D–H···A[b]	H···A	D···A	D–H···A
O1–H1···O48A	1.87	2.86(1)	172
O1–H1···O48B	1.86	2.84(1)	165
O2–H2···N36A[i]	1.91	2.90(1)	173
O2–H2···N36B[i]	2.02	3.01(3)	170
O1w–H11w···O9w	1.90	2.78(1)	175
O2w–H21w···O49A	1.90	2.72(2)	155
O2w–H21w···O49B	1.75	2.63(1)	173
O2w–H22w···O8w	1.94	2.78(1)	167
O3w–H31w···O10w	2.02	2.87(2)	170
O3w–H32w···O12w[i]	1.87	2.70(2)	162
N16A-H16A···O11w	1.82	2.67(3)	163
N26A-H16B···O7w	2.36	3.23(2)	174
N36A-H36A···O12w	1.98	2.77(3)	149
N36A-H36B···O4w[i]	2.22	2.99(2)	145

***π–π* interactions**[c]				
ring – ring	angle	DC	DZ	DXY
2pa···3pa[ii]	4.0	4.09	3.65	1.91

**X–H···*π* interactions**[d]	H–Perp	H···Cg	X–H···Cg	X–H···ring
C42B–H42B···1a[iii]	2.36	3.05	153	114

[a]Symmetry codes: (i) x, 1 – y, 1/2 + z (ii) –x, – y + 2, –z + 2; (iii) 3/2 – x, 1/2 – y, 1 – z. [b]D: donor;A: acceptor. [c]Angle: dihedral angle between the planes (deg), DC: distance between the centroids of the rings (Å), DZ: interplanar distance (Å), DXY: lateral displacement (Å), pa: adenine pentagonal ring, ha: adenine hexagonal ring, a: adenine. [d]H–Perp: Perpendicular distance of H to ring plane; H···Cg: distance of H to the centroid of the ring; X–H···Cg: X–H···Cg angle; X–H···ring: angle of the X–H bond with the aromatic ring.

S2. Analysis of porosity

All the supramolecular architectures show a bidimensional network of voids with dimensions ranging from 3.6 x 5.3 to 7.1 x 7.9 Å that are surrounded by the N-positions of the adeninato ligands, Table S12. The corridors connecting these voids have an approximate radius of 3.0 to 4.1 Å (Figure S13–S15). The volume of these channels, occupied by the crystallization water molecules, has a range between 2449 and 5313 Å^3 per unit cell (29 to 44% of the total volume as calculated by PLATON). A computational analysis shows a pore volume that ranges from 0.206 to 0.375 mL·g^{-1}.

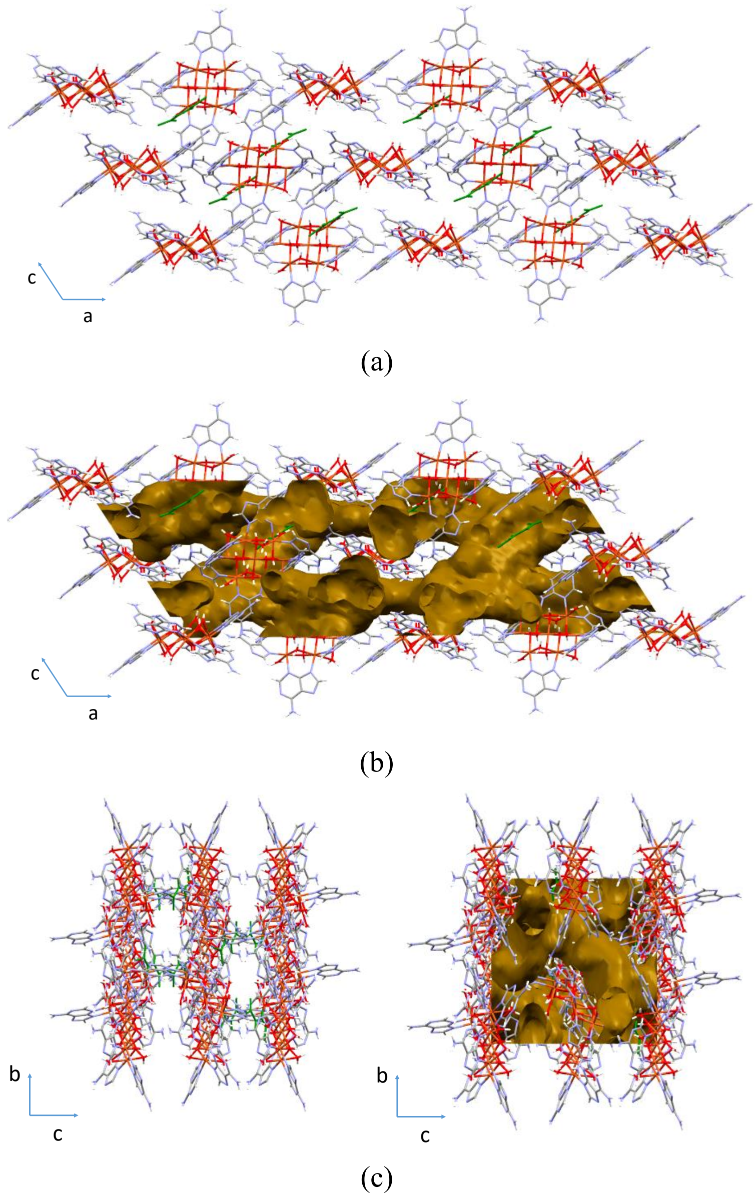

Figure S13. Crystal packing of compound **Cu_F**, highlighting the voids with a mustard color, depicted along different crystallographic directions: a) *b* axis, b) *b* axis highlighting the pores and c) *a* axis.

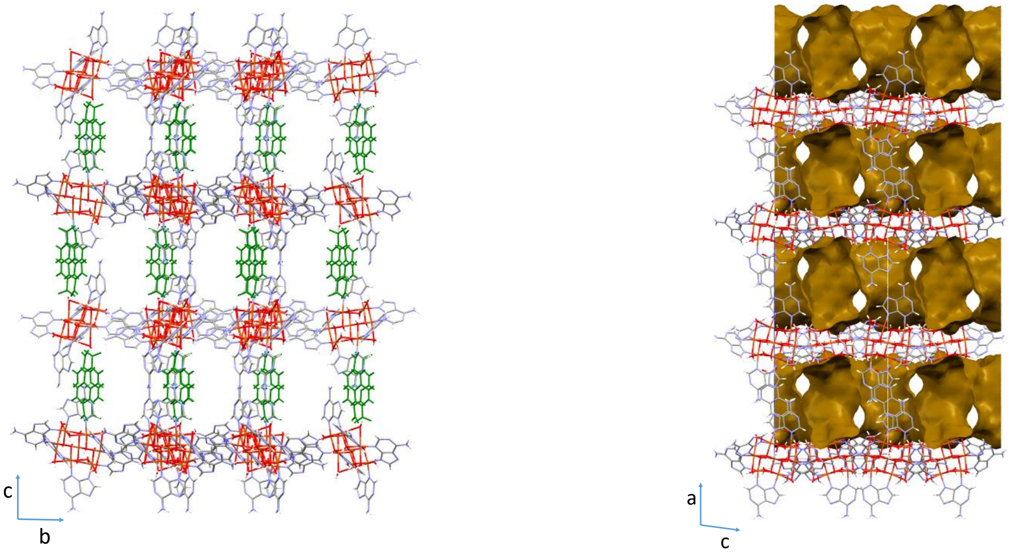

(a) (b)

Figure S14. Crystal packing of compound **Cu_N** (a), highlighting the voids (b) with a mustard color.

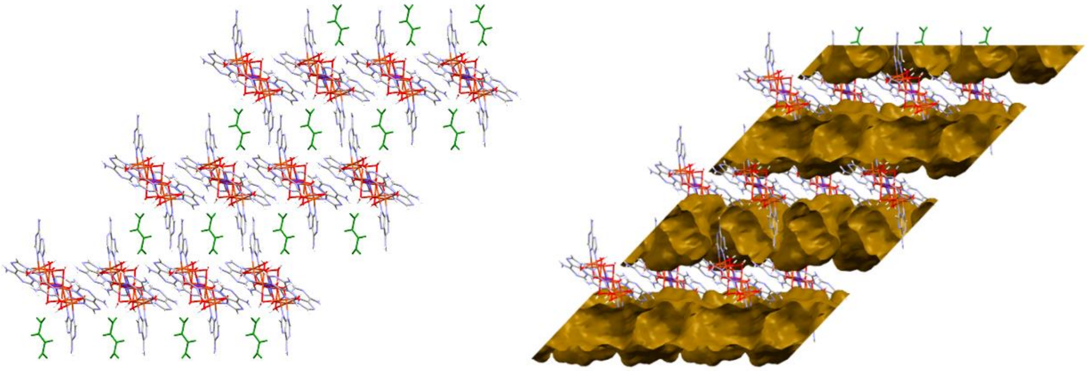

(a)

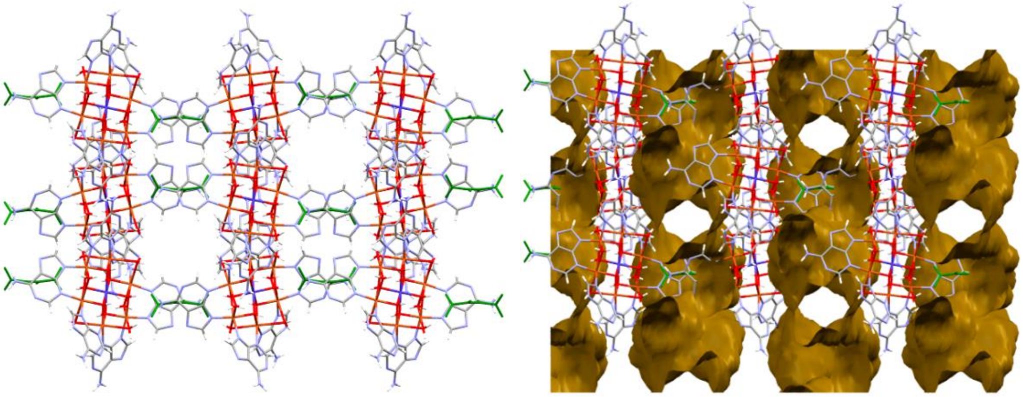

(b)

Figure S15. Crystal packing and voids contact surface of the 2D network of channels (depicted in mustard color) in compound **CuZn_F** along (a) the *b*-axis and (b) the *c*-axis. All the rest heterometallics compounds present the same crystal packing.

Table S12. Experimental data of the computational analysis of the voids of compounds.

Compounds	Pore (Å)	Corridor min/max (Å)	Void volume (Å^3)	Void (%)	Surface area (m^2/g)	Pore volume (mL/g)
**Cu_Fb**	3.6	3.0 x 4.3	4307	29	6.21	0.206
**Cu_N**	7.1 x 7.9	4.1 x 8.1	4171	44	877	0.375
**CuCo_F**	6.1 x 7.1	4.4 x 7.4	2841	36	1365	0.273
**CuNi_F**	4.8 x 5.8	3.4 x 6.3	2449	33	349	0.235
**CuZn_F**	5.1 x 6.4	3.3 x 6.6	2799	36	450	0.267

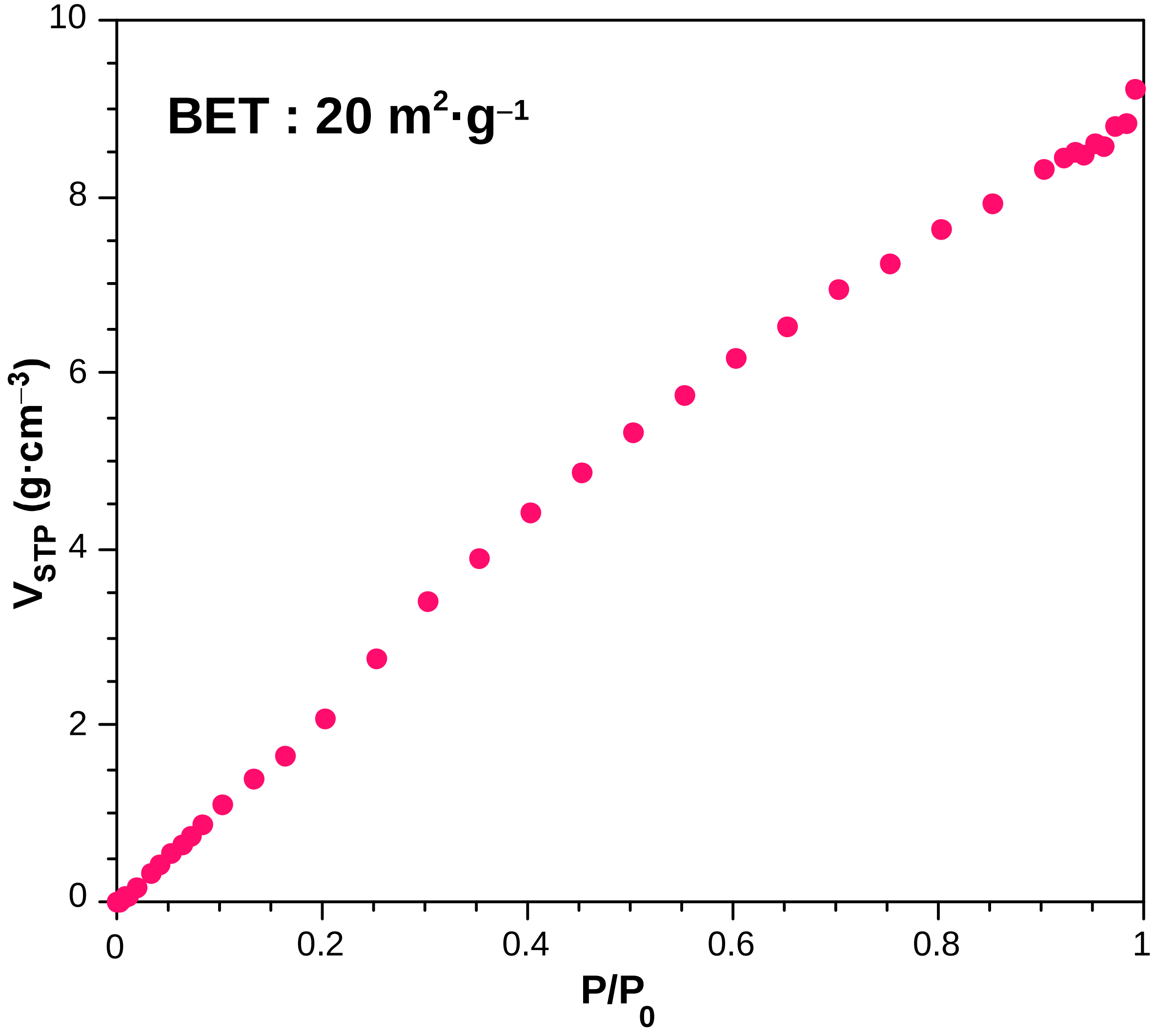

Figure S16. CO_2 adsorption of compound **CuZn_F**.

S3. Fourier Transform Infrared Spectra (FTIR)

Infrared spectroscopy

Spectroscopy has turned out to be a useful technique for the initial characterization of the synthesized compounds, as it allows verifying the existence of the bands corresponding to adenine and the different nucleobases and carboxylate anions. Figure S17 shows the infrared spectra for the compounds presented in this work. Table S13 shows the wavenumbers of the most relevant observed bands[9], their relative intensity and the assignment that has been proposed in each case.

All the compounds exhibit the bands corresponding to the strain vibration of the C–H and N–H bonds of the purine bases in the region of the spectrum between 3400 and 3100 cm^{-1}, in addition to that due to the O–H bonds of the water molecules. Between 1700 cm^{-1} are the bands of the nucleobase ligands for the groups of amino. Around 1640 cm^{-1}, the asymmetric tension of the carboxylate group of the carboxylate anions are observed. The presence of the peak located around 1600 cm^{-1} corresponding to the vibration of the C=C bond and the deformation of the NH_2 group is noteworthy, which allows the identification of adenine in all the compounds.[10]

The vibrational bands of the M–N bonds appear in all compounds below 550 cm^{-1}.

Table S13. FTIR assignation of compounds present in this paper.

Adenine	Fumaric acid	Naphthalene-2,6-dicarboxylic acid	Cu_F	Cu_N	CuCo_F	CuNi_F	CuZn_F	Assignment
			3430vs	3440vs	3360vs	3360vs	3350vs	υ O—H,
3296s	—	—	3210s	3340w	3210vs	3200vs	3200vs	υ N—H
2930w	2925w	3100w	2920w	3190vs	2920w	2920w	2920w	υ_{as} C—H
1670vs	1699vs	—	1640vs	1640vs	1640vs	1640vs	1640vs	υ_{as} C=O + δ NH_2
1600vs	1620s	1694vs	1610m	1600m	1610s	1600w	1600w	υ C=C+ υ C=N
—	1540w	1605m	1550s	1540s	1550s	1550vs	1550vs	υ_s C=O
1504s	—	1570w	1500w	1490m	1500w	1500w	1500w	υ C—NH_2
—	1496vs	—	1470s	1460vs	1460vs	1460vs	1460vs	υ_{as} COO^-
1420vs	1406m	1504s	1400s	1400vs	1390vs	1400vs	1400vs	δ_{ring} + δ C—H
—	1382vs	1420vs	1380m	1340s	1370s	1340s	1340s	υ_s COO^-
—	1296s/1152s	1344vs	1306m/1150m	1270m/1140s	1300m/1150s	1310m/1150m	1310m/1140s	υ_s C—O
1230ss	1202s	1290vs/1140s	1195s	1200s	1200vs	1200s	1200vs	δ_{ip} CCH
1020vs	1050s	1043s	1031m	1040m	1030m	1030m	1030m	γ C—H + γ NH_2 + υ C—C_{arom}
—	990m	990m	970m	980m	980m	980m	980m	υ_s COO^-
930vs	933m	920m	933w	930w	930w	940w	940w	δ C—H, δ C—C
790vs	782m	780m	795m	790m	790m	800m	800m	δ_{ip} C—H, ω NH_2
720s/640vs	740m/660s	750vs/630m	740m/655m	740m/650m	740m/660m	740m/660m	740m/660m	δ_{ip} ring defor., δ_{oop} COO^-
			560m/455m	550m/450m	560m/460m	560m/440m	550m/420m	υ M—N

[a]vs: very strong, s: strong, m: medium, w: weak. [b]s: symmetric, as: antisymmetric, υ: stretching vibration, δ: bending vibration, γ:rocking, ω: wagging, ip = in plane, oop = out of plane.

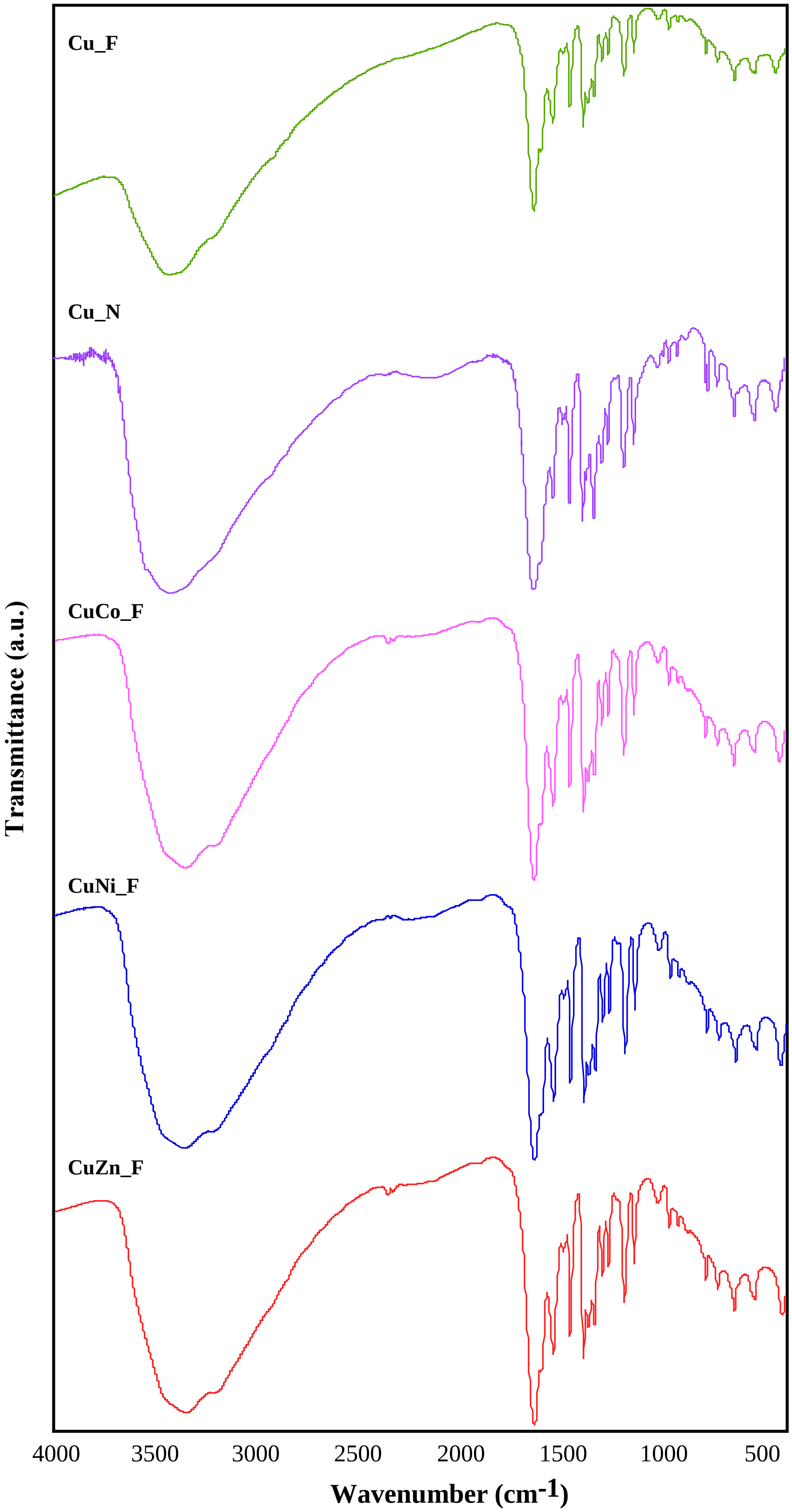

Figure S17. FTIR spectra of compounds presented in this work.

S4. Thermogravimetric analysis (TG)

Thermogravimetric analysis were performed in synthetic air from 25 °C to 600 °C with a temperature increase rate of 5 °C·min^{-1} (Figures S18; Table S14). In all the compounds containing the fumarate anion, the thermograms show four main weight loss stages. First, in two overlapped stages crystallization solvent and coordination water molecules are released at a temperature range of 25–200 °C. Thereafter, in two overlapped stages, the deamination of the adeninato ligands and the loss of three more water molecules due the dihydroxylation of three hydroxide group of the cluster at a range of dehydration occurred at a value range of 180–290 °C and 115–210 ºC, respectively.

Finally, the identification of the final product was performed by PXRD analysis on powder sample of all the above compounds. In all the compounds a very exothermic final decomposition stop takes place to lead to CuO (PDF: 48-1548) as final residue at 450 °C with the correspondent metal oxide oxide (Co_3O_4: PDF 80-1541; NiO: PDF 47-1049; ZnO PDF 36-1451), respectively.

Table S14. Thermoanalytic data for compounds **Cu_F**, **Cu_N** and **CuZn_F**.[a]

**Step**	**T_i**	**T_{peak}**	**T_f**	**ΔH**	**Δm(%)**	**ΣΔm(%)**	**$\Sigma\Delta m(\%)_{theor}$**
**Cu_F**							
**1**	25	70/105	130	Endo	22.10	22.10	22.25 (–25 H_2O_{cryst})
**2**	130	150	200	Exo	5.20	27.30	27.59 (–6 H_2O_{coord})
**3**	200	230	250	Exo	4.91	32.21	32.27 (–6 NH_2)
**4**	250		350		2.87	35.08	35.00 (–3 H_2O_{hydr})
**5**	350	421	600	Exo	37.39	72.47	72.49 (CuO)
**Cu_N**							
**1**	25	75	125	Endo	27.54	27.54	27.52 (–27 H_2O_{cryst} + –6 H_2O_{coord})
**2**	125		180		2.59	30.13	30.02 (–3 H_2O_{hydr})
**3**	180	230	290	Exo	4.17	34.30	34.46 (–6 NH_2)
**4**	300	380	600	Exo	39.92	74.22	74.22 (CuO)
**CuCo_F**							
1	25	100	135	Endo	21.90	21.90	22.09 – (17 H_2O_{cryst} + 6 H_2O_{coord})
2	135	145	190	Endo	3.04	24.94	24.97 – (3 H_2O_{hydr})
3	190	230	250	Exo	5.19	30.13	30.09 – (6 NH_2)
4	335	405	600	Exo	40.26	70.39	70.60 (CuO, CoO)
**CuNi_F**							
1	25	100	160	Endo	23.38	23.38	23.56 – (19 H_2O_{cryst} + 6 H_2O_{coord})
2	160		210		2.60	25.98	26.39 – (3 H_2O_{hydr})
3	210		280		5.22	31.20	31.41 – (6 NH_2)
4	330	420	600	Exo	39.70	70.90	71.12 (CuO, NiO)
**CuZn_F**							
**1**	25	95	115	Endo	20.00	20.00	20.01 (–27 H_2O_{cryst} + –3 H_2O_{coord})
**2**	115	160	200	Endo	5.72	25.72	25.54 (–3 H_2O_{coord} + –3 H_2O_{hydr})
**3**	200	230	260	Exo	4.93	30.65	30.59 (–6 NH_2)
**4**	325	440	600	Exo	40.08	70.73	70.60 (CuO, ZnO)

[a] T_i = initial temperature; T_{peak} = DTA peak temperature; T_f = final temperature; ΔH = type of process according to DTA, Δm(%) = mass loss percentage for each process; ΣΔm(%) = total mass loss percentage; $\Sigma\Delta m(\%)_{theor}$ = theoretical total mass loss percentage.

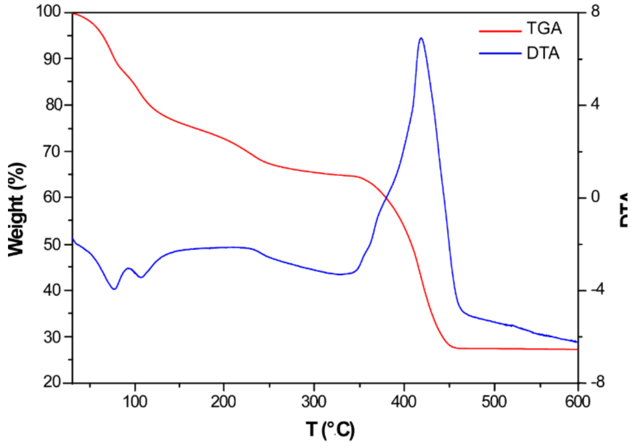

(a)

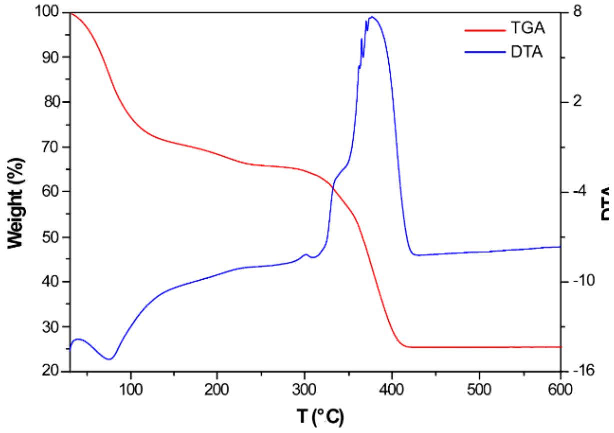

(b)

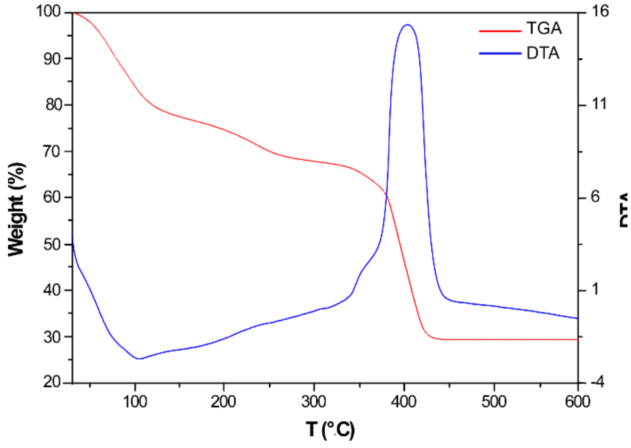

(c)

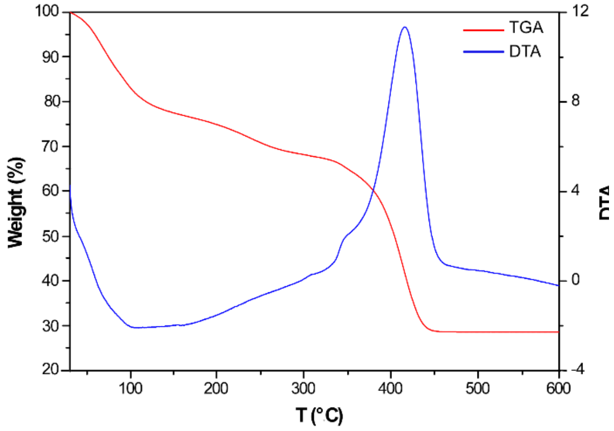

(d)

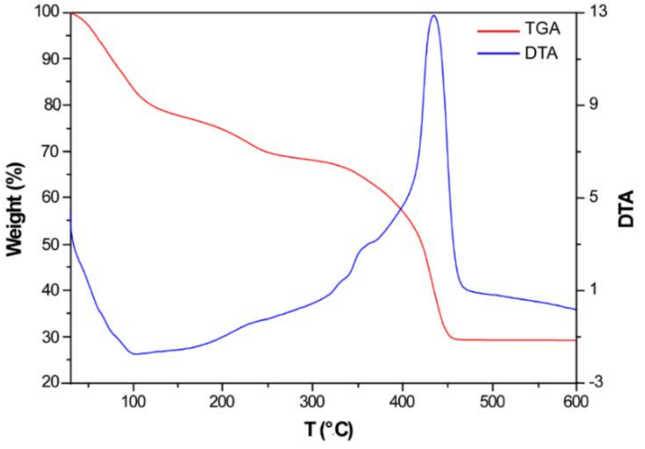

(e)

Figure S18. Thermogravimetric data (TGA-DTA curves) for compounds (a) **Cu_F**, (b) **Cu_N**, (c) **CuCo_F**, (d) **CuNi_F** and (e) **CuZn_F**.

S5. Temperature variable PXRD experiments (TDX).

The variable-temperature X-ray diffraction data of fumarate containing homometallic compound (F) show that the release of the crystallization water molecules not means a substantial decrease of crystallinity, maintaining the crystallinity of the structure even after losing the coordination water molecules around 200 °C. The structure remains until a temperature above 220 °C, Figure S19.

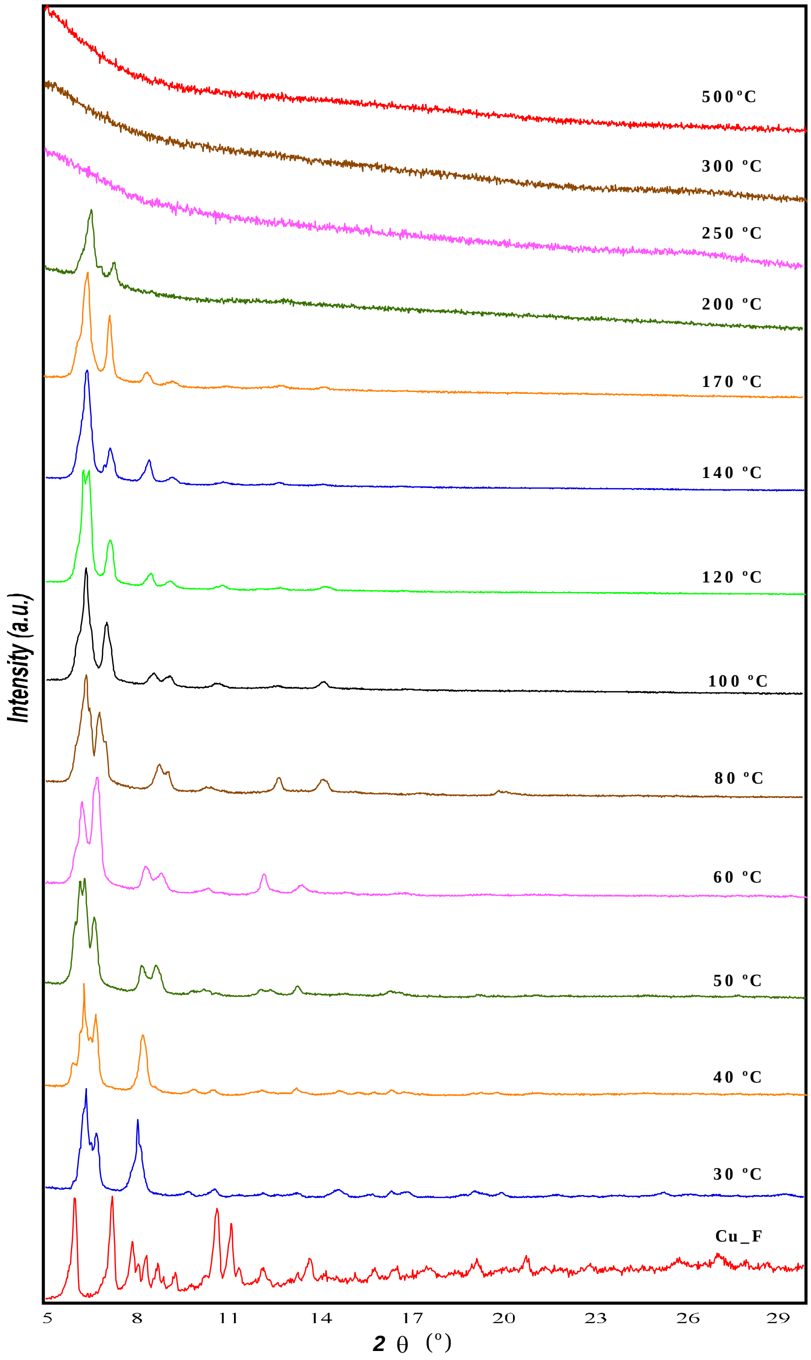

Figure S19. Variable-temperature X-ray diffraction patterns for compound **Cu_F**.

The variable-temperature X-ray diffraction data of naphthalene-2,6-dicarboxylate anion containing compound show that the release of the crystallization water molecules does not mean a substantial decrease of crystallinity. The diffraction peaks remain well defined until the temperature reaches 210 °C, where they start to collapse leading to an amorphous material, (Figure S20).

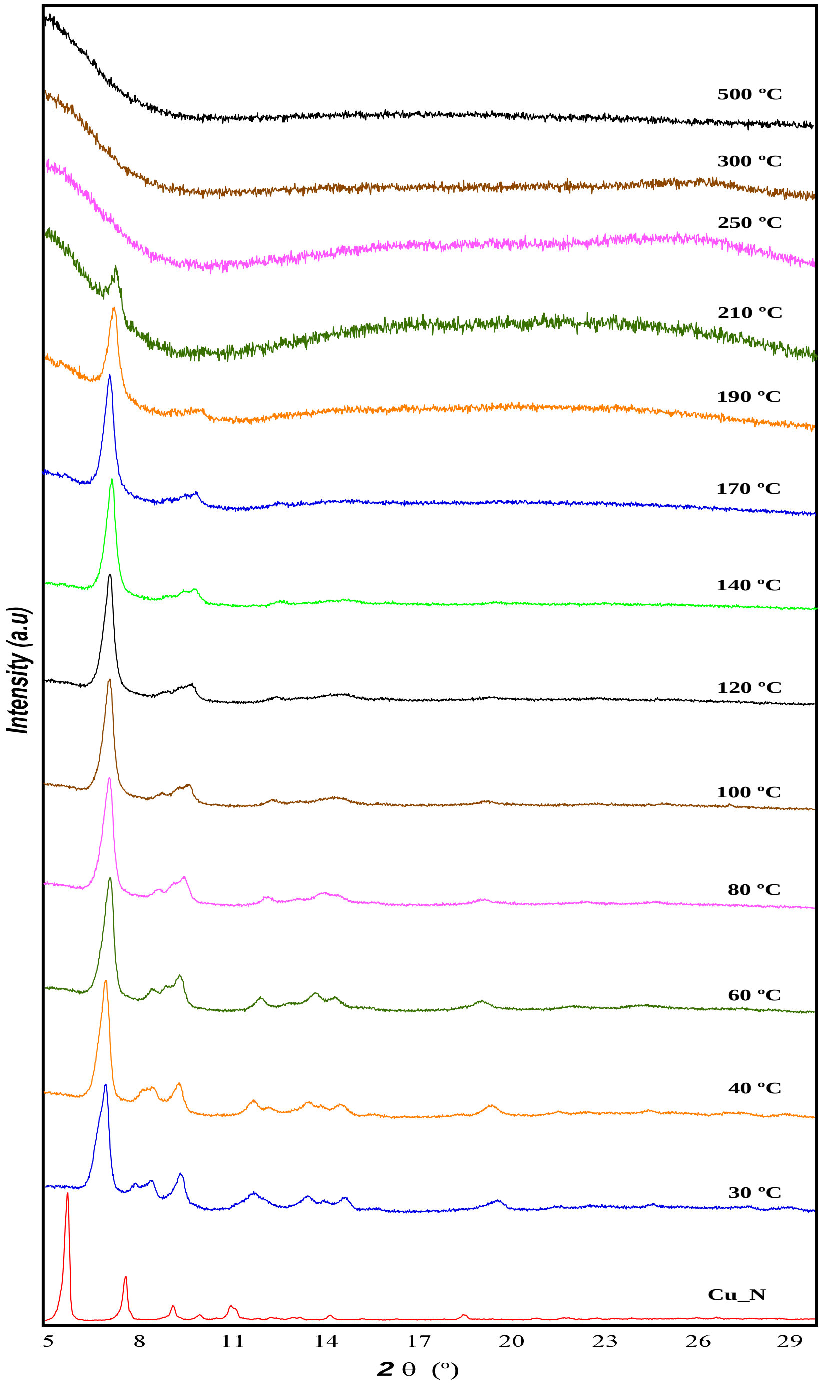

Figure S20. Variable-temperature X-ray diffraction patterns for compound **Cu_N**.

The variable-temperature X-ray diffraction data of heterometallic compounds **CuCo_F**, **CuNi_F** and **CuZn-F** show that the release of the crystallization water molecules, at around 60 °C, involves a substantial structural change with a significantly different diffraction pattern. Later, at 100 ºC with the loss of the coordinated water molecules a new change takes place to provide a new crystalline phase that remains unaltered up to 210 °C, where the diffraction peaks disappear and an amorphous product is obtained (Figure S21 and S22).

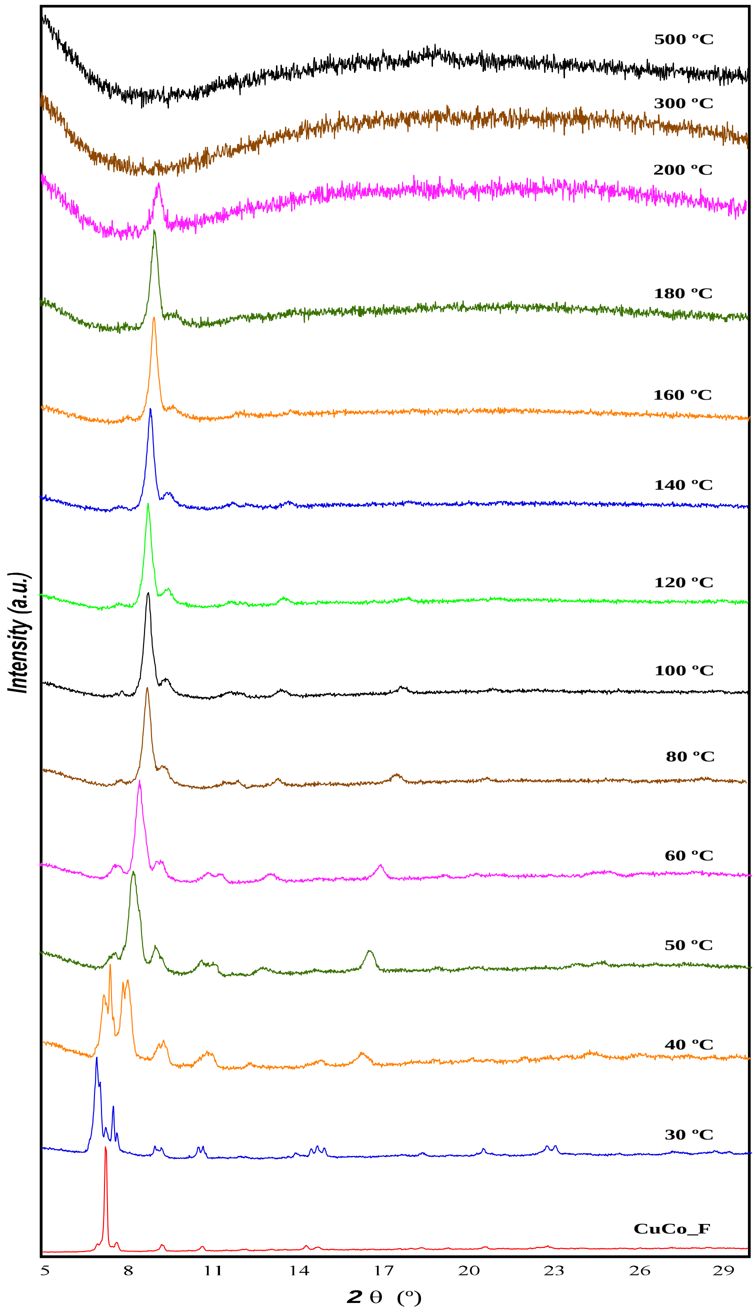

Figure S21. Variable-temperature X-ray diffraction patterns for compound **CuCo_F**.

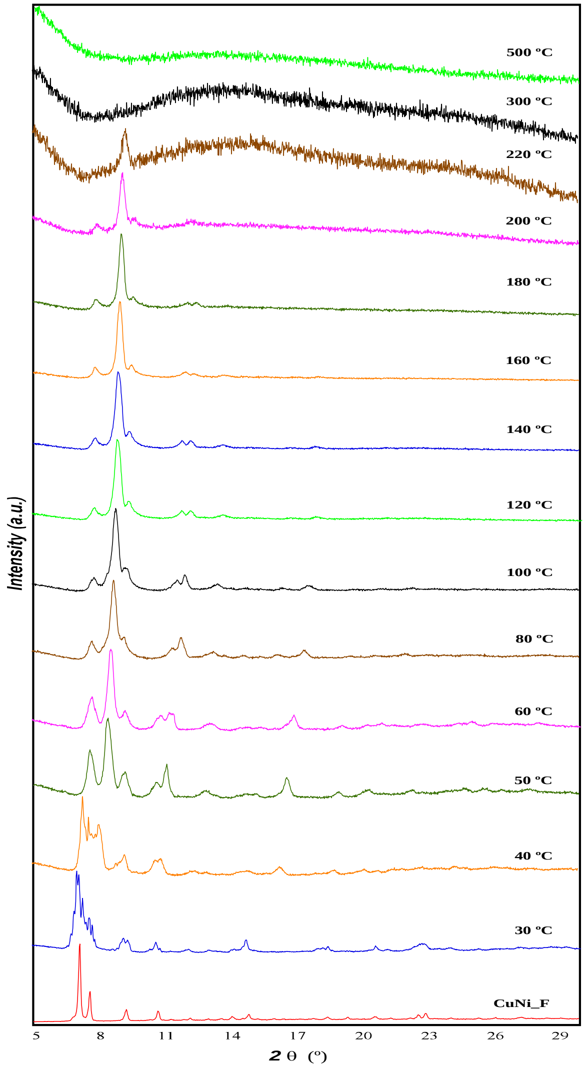

(a)

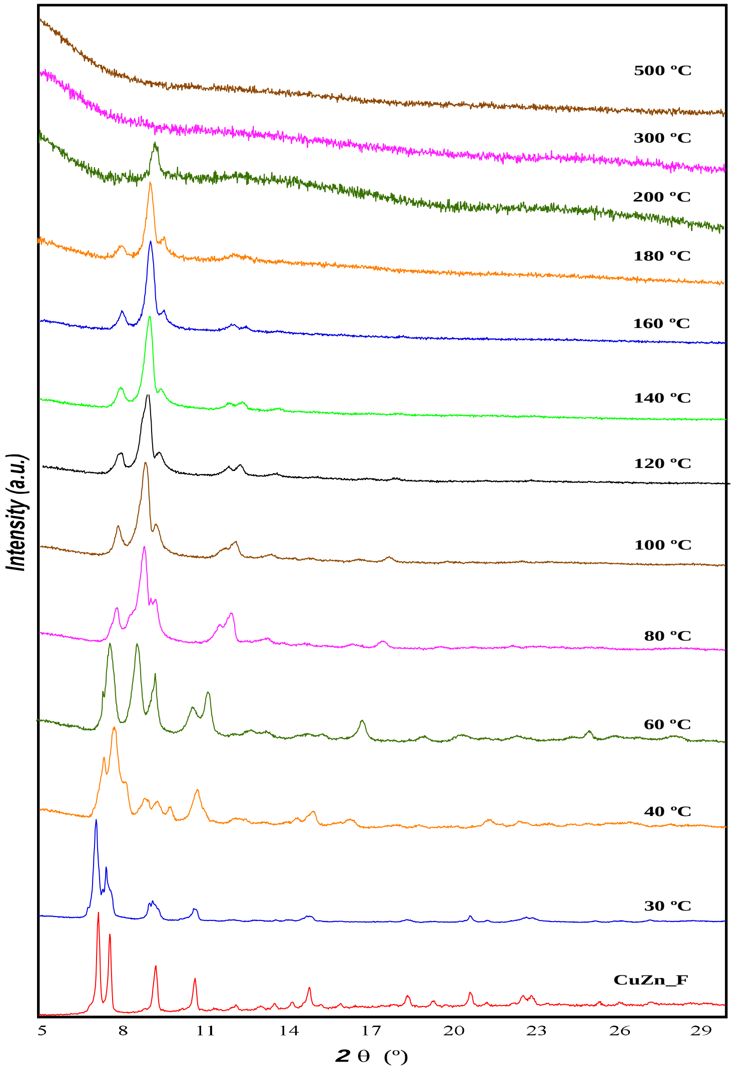

(b)

Figure S22. Variable-temperature X-ray diffraction patterns for compound (a) **CuNi_F** and (b) **CuZn_F**.

S6. X-Ray Photoelectron Spectroscopy (XPS)

X-Ray Photoelectron Spectroscopy (XPS) measurements were performed on a SPECS system (Berlin, Germany) equipped with a Phoibos 150 1D-DLD analyzer and monochromatic radiation source Al Kα (1486.7 eV), Figure S23 - S25, Table S15.

An initial analysis was carried out to determine the elements present (wide scan: step energy 1 eV, dwell time 0.1 s, pass energy 80 eV) and a detailed analysis of the detected elements (detail scan: step energy 0.08 eV, dwell time 0.1 s, pass energy 30 eV) with an electron exit angle of 90º. The spectrometer was previously calibrated with Ag (Ag $3d_{5/2}$, 368.26 eV).

The spectra were fitted using CasaXPS 2.3.16 software, which models the Gauss-Lorentzian contributions, after background subtraction (Shirley background subtraction). The concentrations were calculated were calculated by correcting the values with relative atomic sensitivity factors (Scofield). The reduction conditions of the samples in the cell were:

- Ramp up to 450 ºC, 1 hour, 1 bar, 300 mL·min^{-1} H_2/Ar (20%), continuous.
- Isotherm 450 ºC, 1 hour, 1 bar, 300 mL·min^{-1} H_2/Ar (20%), continuous.

Table S15. Experimental data of compound **Cu@F** and **CuZn@F** before and after the reaction.

**Cu@F**				
	**Before reaction**		**After reaction**	
**Element**	Binding energy (eV)	Atom percentage (%)	Binding energy (eV)	Atom percentage (%)
**C 1s**	291.4	69.2	291.9	70.2
**O 1s**	530.5 / 531.6 / 533.1	14.0	531.8	1.8
**N 1s**	398.4 / 400.4	8.5	398.4 / 400.3	11.3
**Cu^{I} $2p_{3/2}$**<br>**Cu^{II} $2p_{3/2}$**	932.4<br>934.5	8.3	932.1	16.7
**CuZn@F**				
	**Before reaction**		**After reaction**	
**Element**	Binding energy (eV)	Atom percentage (%)	Binding energy (eV)	Atom percentage (%)
**C 1s**	292.3	62.8	292.3	60.4
**O 1s**	530.6 / 531.7 / 533.2	18.0	531.5	7.2
**N 1s**	398.5 / 400.4	9.4	398.5 / 400.5	12.4
**Cu^{I} $2p_{3/2}$**<br>**Cu^{II} $2p_{3/2}$**	932.3<br>934.4	7.5	932.1	15.9
**Zn $2p_{3/2}$**	1021.6	2.3	1020.9 / 1022.2	4.1

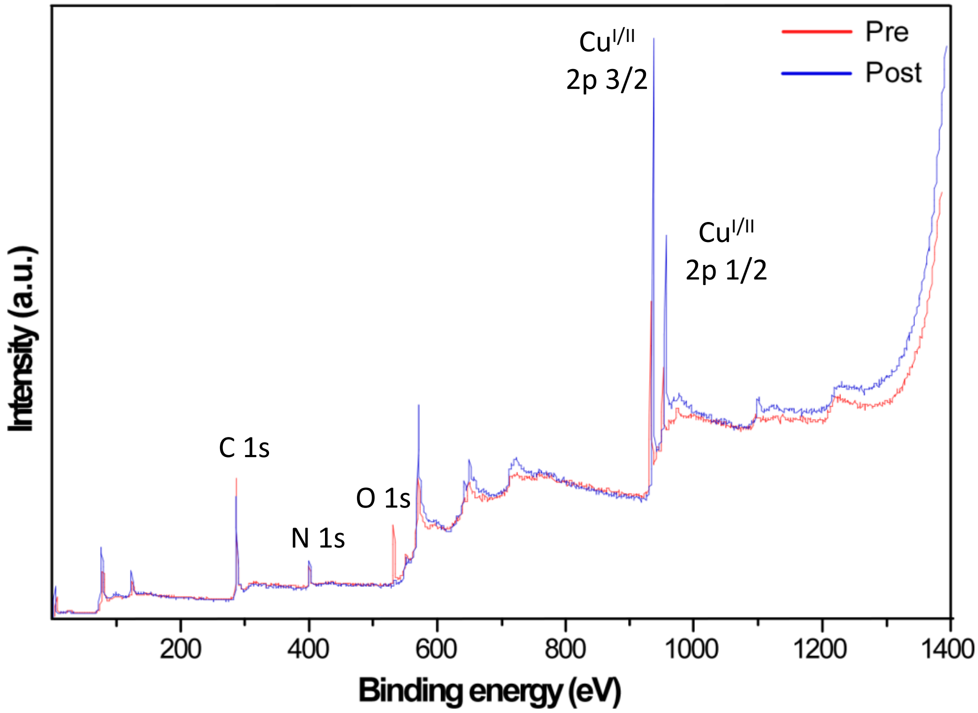

(a)

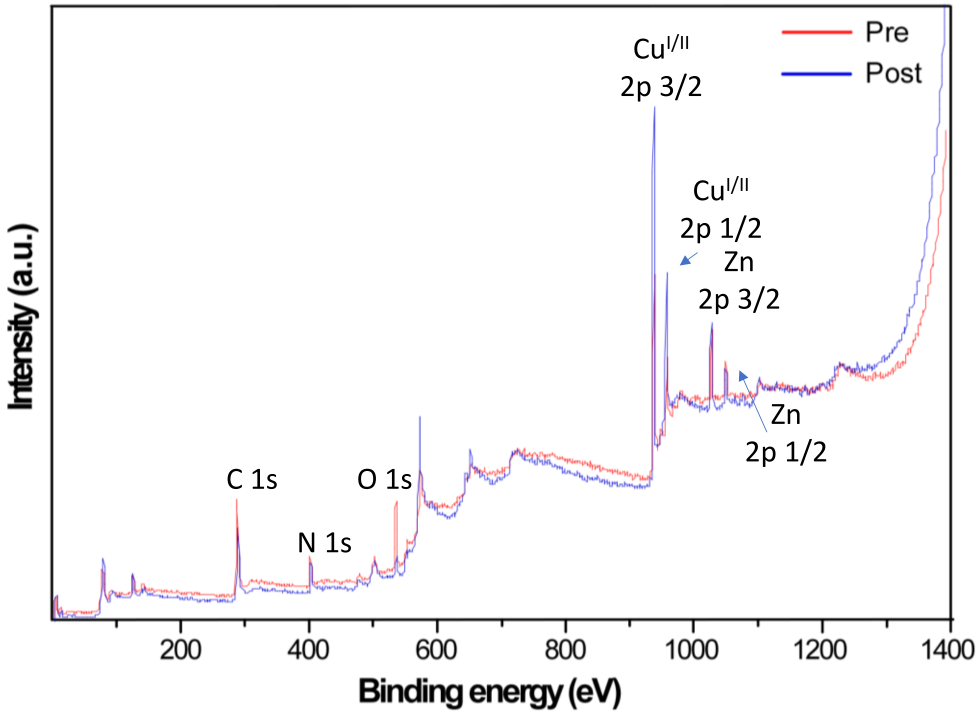

(b)

Figure S23. Representation of the XPS spectra of (a) **Cu@F** and (b) **CuZn@F.**

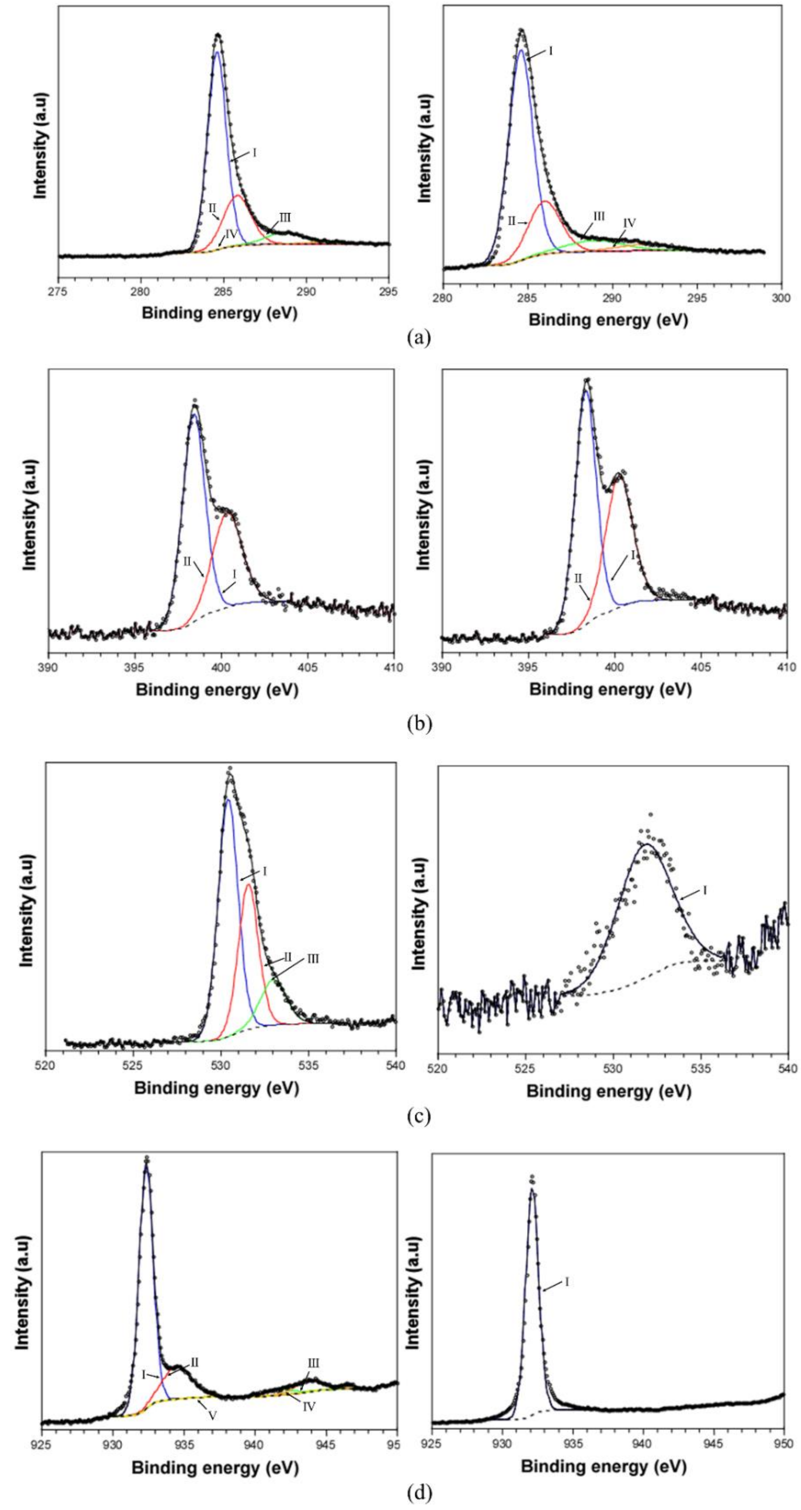

Figure S24. Representation of the XPS spectra for (a) carbon, (b) nitrogen, (c) oxygen and (d) copper of compound **Cu@F** pre- (left) and post- (right) catalysis.

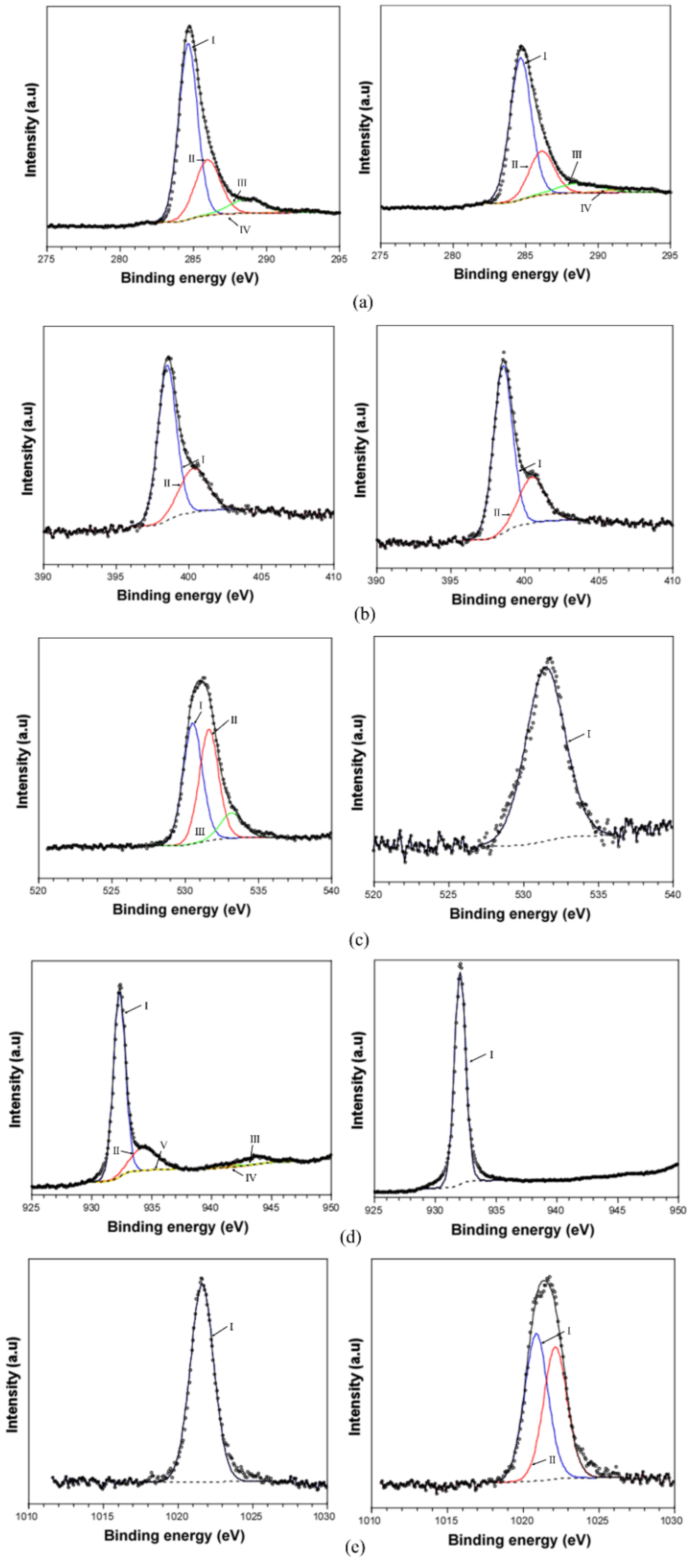

Figure S25. Representation of the XPS spectra for (a) carbon, (b) nitrogen, (c) oxygen, (d) copper and (e) zinc of compound **CuZn@F** pre- (left) and post- (right) catalysis.

S7. Powder X-ray Diffraction (PXRD)

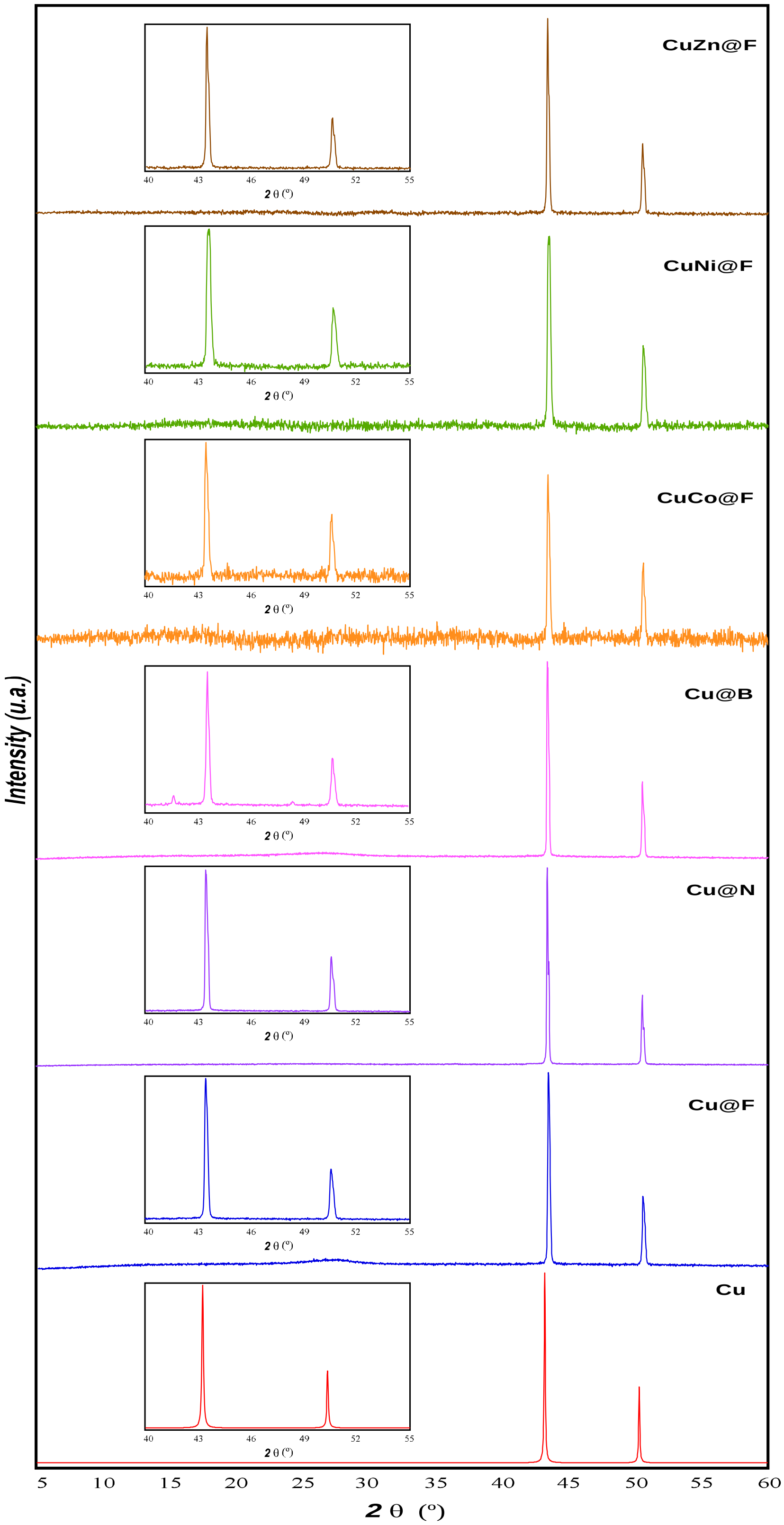

Figure S26. Powder X-ray diffraction patterns: (bottom) simulated pattern from single-crystal of Cu and experimental pattern measured on filtered samples of compounds **Cu@F**, **Cu@N**, **Cu@B**, **CuCo@F**, **CuNi@F**, **CuZn@F** (top). Depicted in the insets an amplification of the 40 – 55° area for a better comparison.

S8. Transmission electron microscopy (TEM)

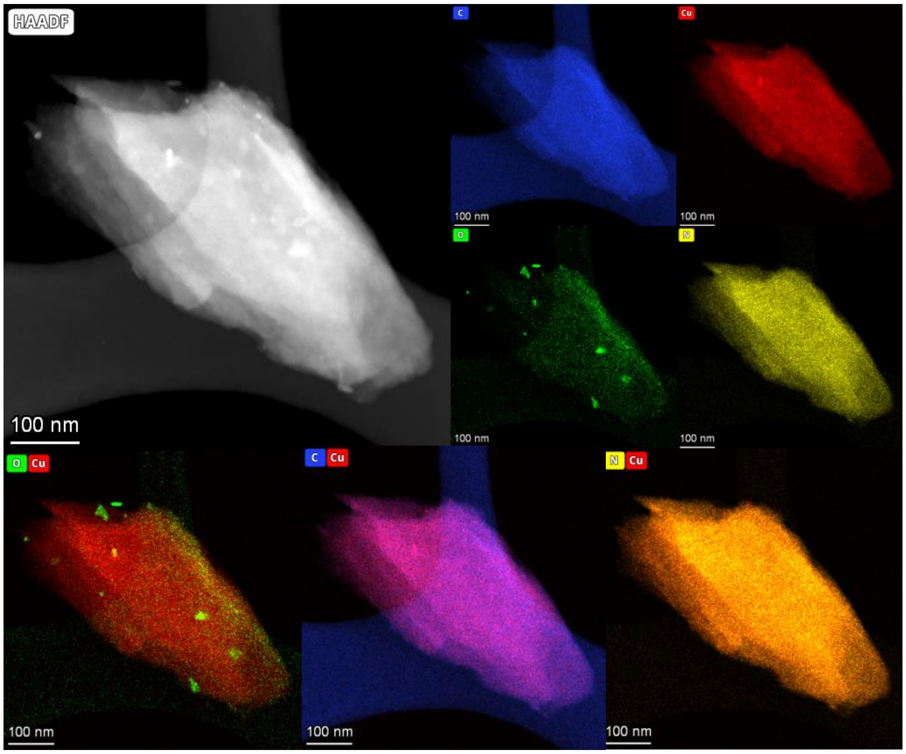

(a)

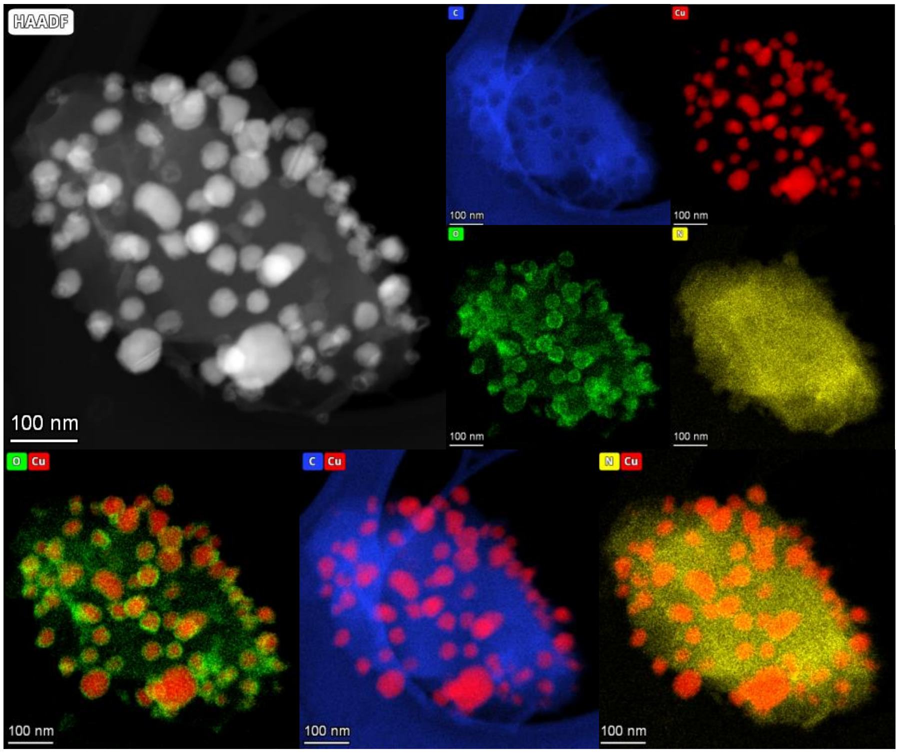

(b)

Figure S27. High-angle annular dark field TEM images of catalyst (a) before (**Cu@F**) and (b) after (**Cu@F$_p$**) the reaction. Note that a thin oxide layer is observed at the surface of the nanoparticles due to the exposure of the catalyst to the open atmosphere after the catalytic reaction.

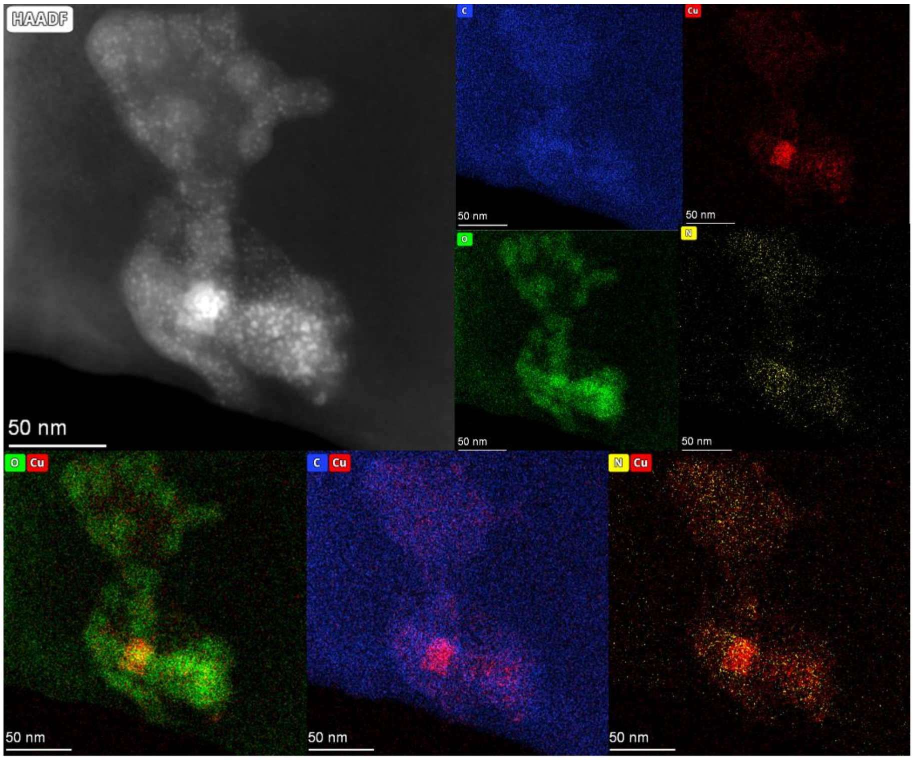

(a)

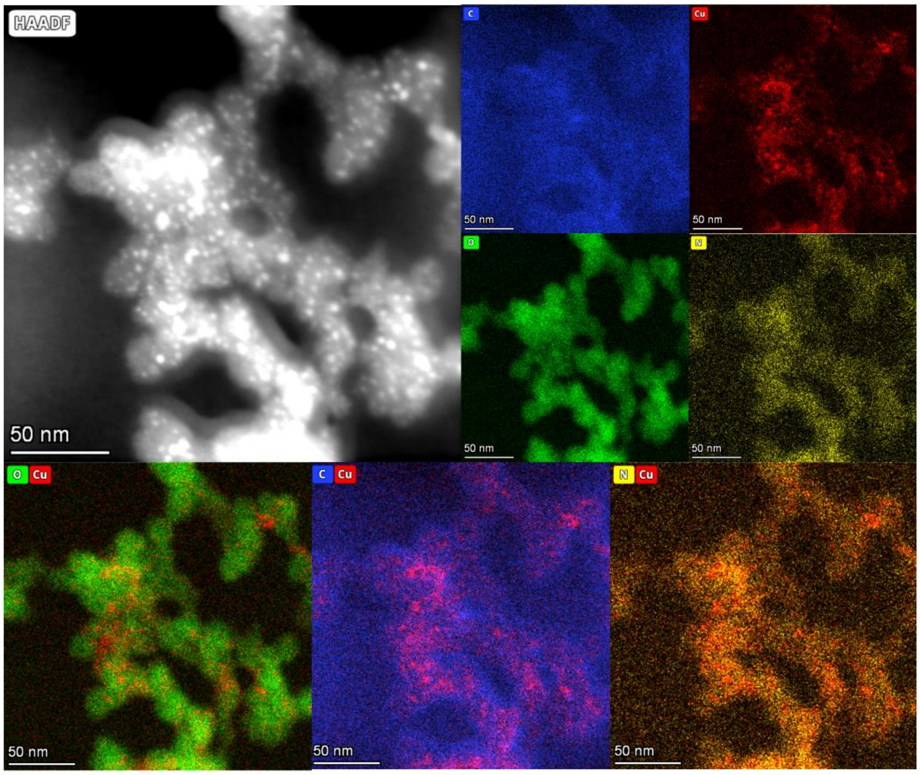

(b)

Figure S28. High-angle annular dark field TEM images of catalysts (a) **Cu@N** and (b) **Cu@B** before the reaction.

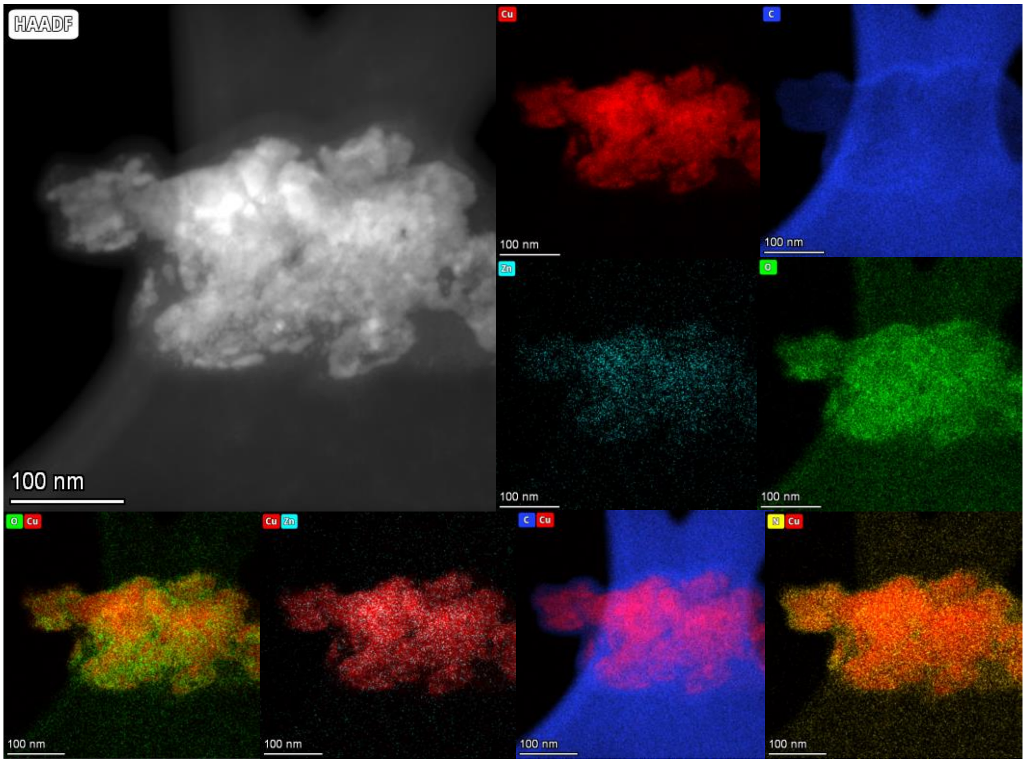

(a)

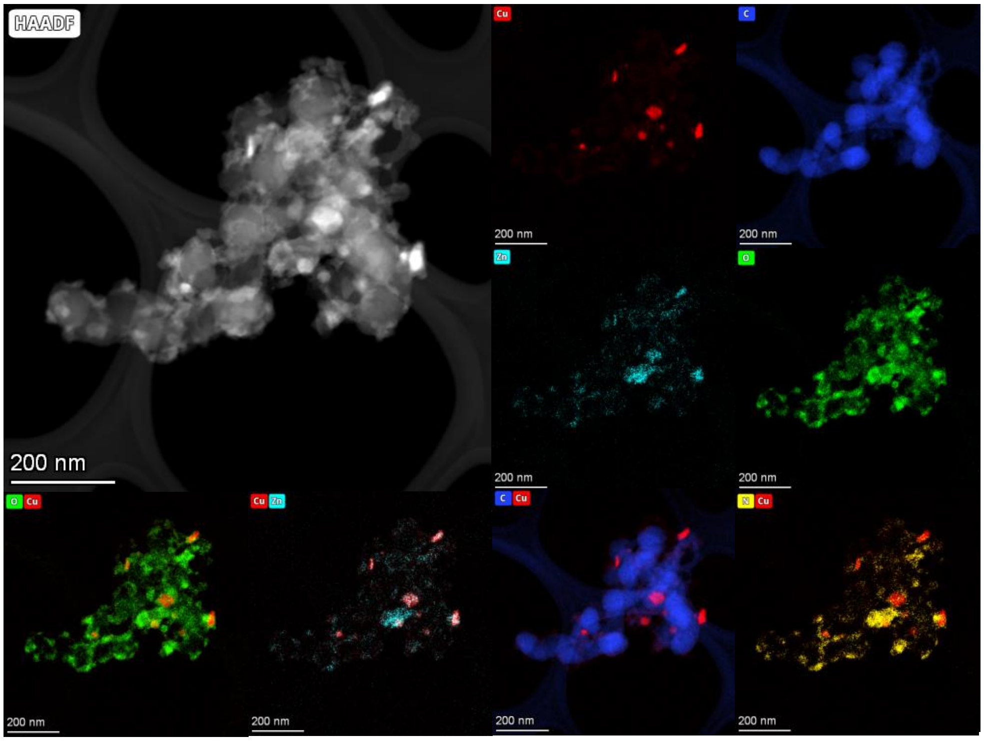

(b)

Figure S29. High-angle annular dark field TEM images of catalyst (a) before (**CuZn@F**) and (b) after (**CuZn@F$_p$**) the reaction.

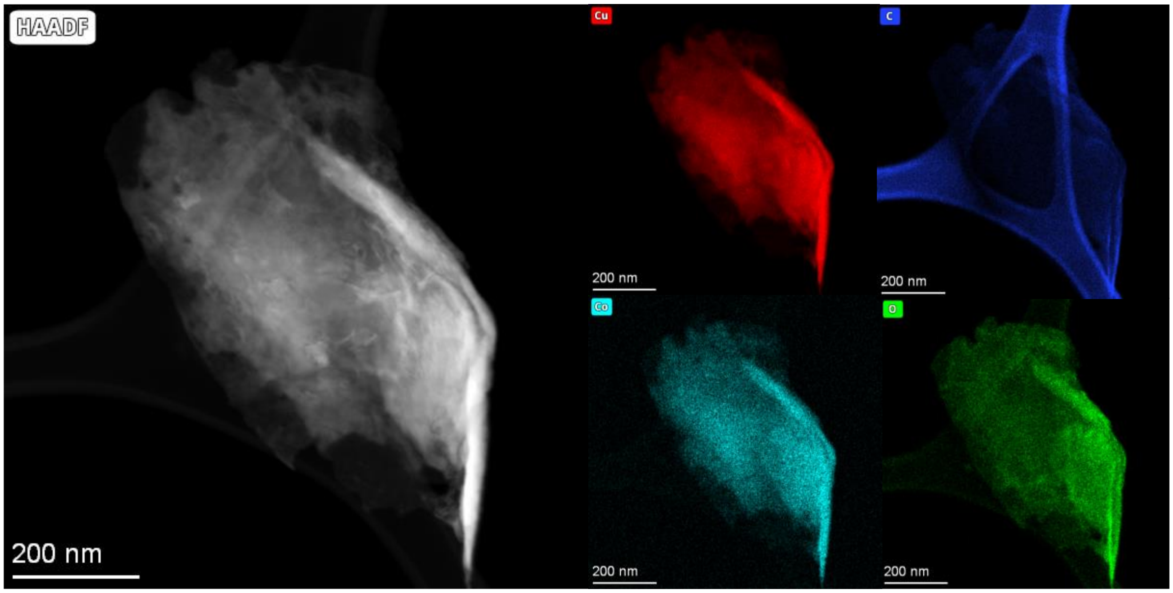

(a)

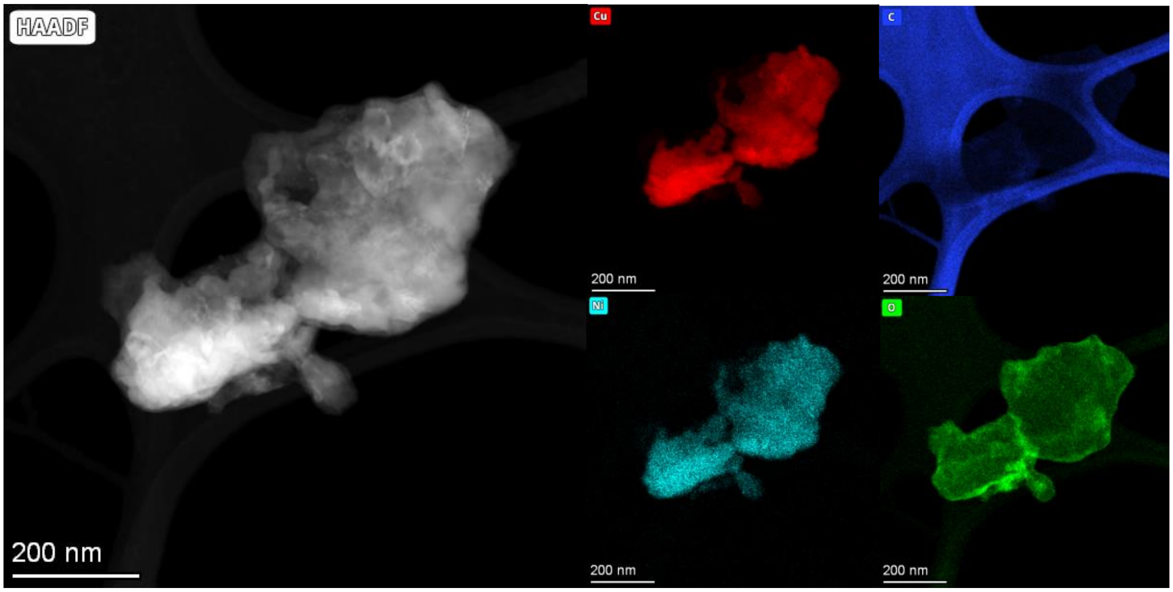

(b)

Figure S30. High-angle annular dark field TEM images (left) and the corresponding EDX elemental maps (right) for the catalyst derived from the heterometallic catalysts: (a) **CuCo@F** and (b) **CuNi@F.**

S9. Catalysis results

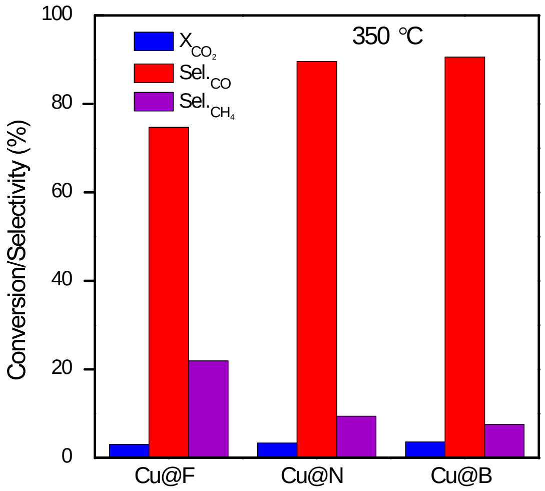

(a)

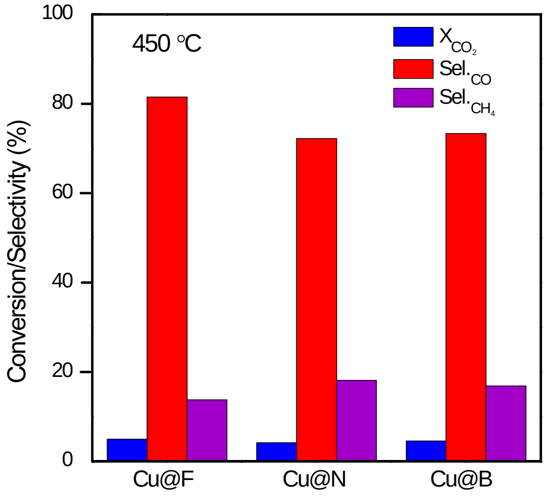

(b)

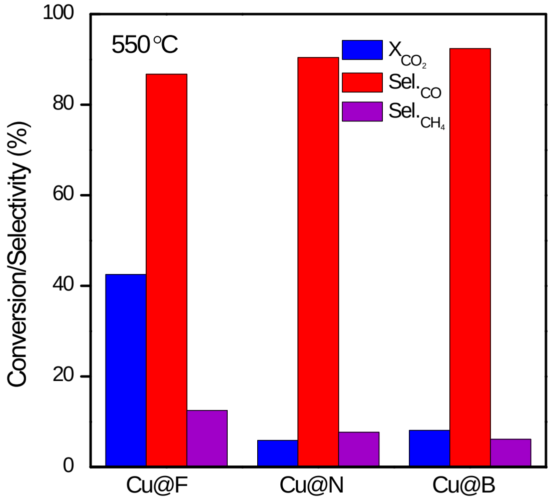

(c)

Figure S31. Results of the reverse water gas shift reaction at 350 °C (a), 450 °C (b) and 550 °C (c) using the homometallic Cu **Cu@F**, **Cu@N** and **Cu@B** catalysts: conversion (blue) and CO (red) and CH_4 (purple) selectivity.

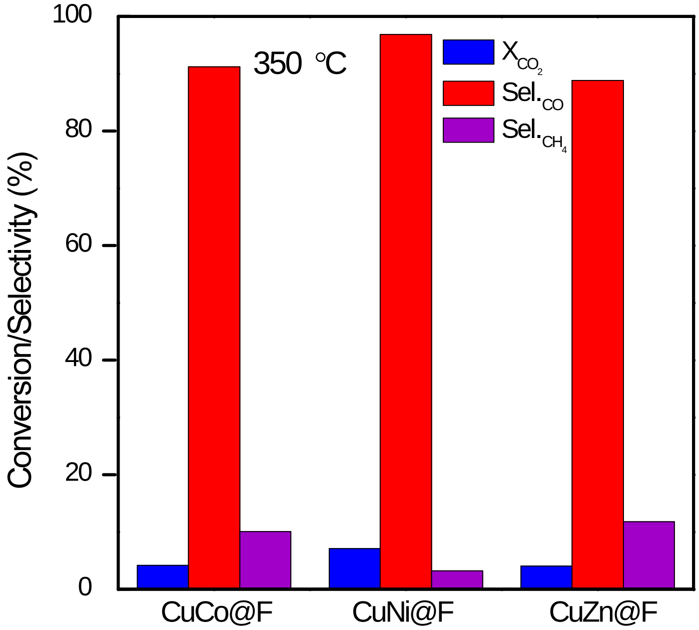

(a)

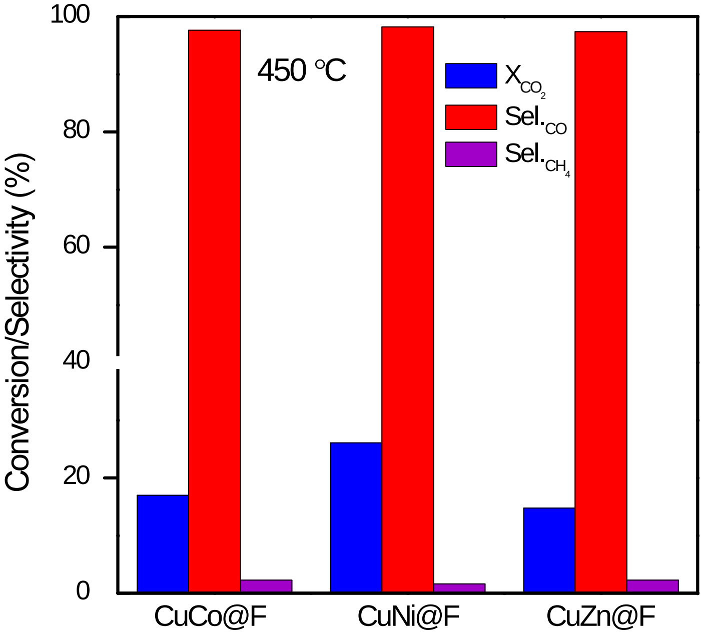

(b)

Figure S32. Results of the reverse water gas shift reaction at 350 °C (a) and 450 °C (b) using the heterometallic **CuCo@F**, **CuNi@F** and **CuZn@F** catalysts: conversion (blue) and CO (red) and CH_4 (purple) selectivity.

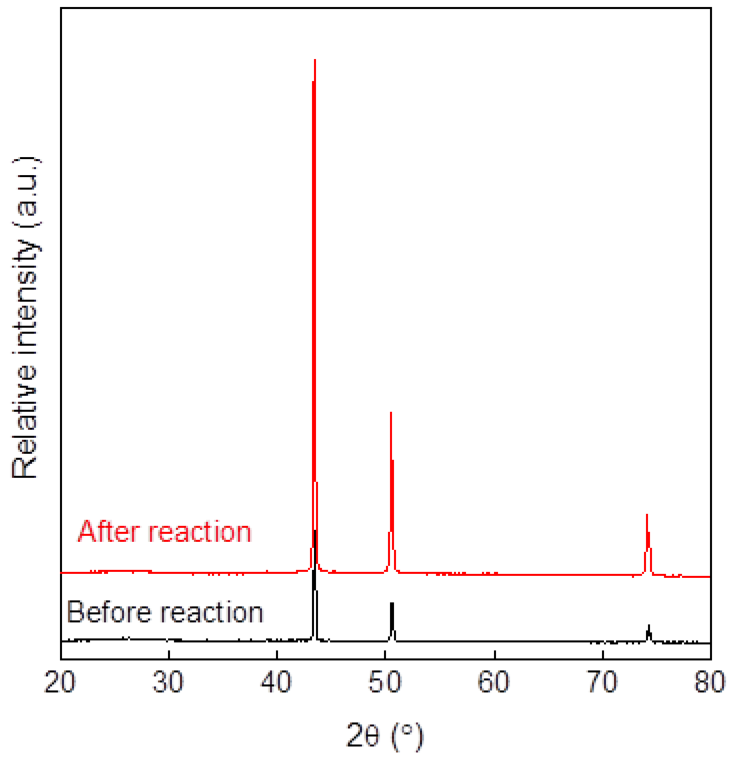

Figure S33. Powder XRD patterns of heterometallic Cu-Zn catalyst (**CuZn@F**) before and after 50 h of reverse water gas shift experiment.

S10. References

(1) Nardelli M. Modeling hydroxyl and water H atoms. *J. Appl. Crystallogr.* **1999**, *32*, 563-571.

(2) Farrugia, L. J. WinGX and ORTEP for Windows: An Update. *J. Appl. Crystallogr.* **2012**, *45*, 849–854.

(3) CrysAlisPRO, Oxford Diffraction /Agilent Technologies UK Ltd, Yarnton, England.

(4) Altomare, A.; Cascarano, G.; Giacovazzo, C.; Guagliardi, A.; Burla, M. C.; Polidori, G.; Camalli, M. SIR92 - a program for automatic solution of crystal structures by direct methods. *J. Appl. Crystallogr.* **1994**, *27*, 435.

(5) Sheldrick, G. M. A short history of SHELX. *Acta Cryst.* **2008**, *A64*, 112–122.

(6) Spek, A. L. PLATON SQUEEZE: a tool for the calculation of the disordered solvent contribution to the calculated structure factors. *Acta Cryst.* **2015**, *C71*, 9–18.

(7) Spek, A. L. Single-crystal structure validation with the program PLATON. *J. Appl. Crystallogr.* **2003**, *36*, 7–13.

(8) (a) Macrae, C. F.; Edgington, P. R.; McCabe, P.; Pidcock, E.; Shields, G. P.; Taylor, R.; Towler, M.; Van De Streek, J. Mercury: visualization and analysis of crystal structures. *J. Appl. Crystallogr.* **2006**, *39*, 453–457. (b) MacRae, C. F.; Sovago, I.; Cottrell, S. J.; Galek, P. T. A.; McCabe, P.; Pidcock, E.; Platings, M.; Shields, G. P.; Stevens, J. S.; Towler, M.; Wood, P. A. Mercury 4.0: from visualization to analysis, design and prediction. *J. Appl. Crystallogr.* **2020**, *53*, 226–235.

(9) (a) Ghose, R. Complex formation of adenine-uracil base pair with some transition metal ions. *Inorg. Chim. Acta* **1989**, *156*, 303–306. (b) Nakamoto, K. Infrared and Raman Spectra of Inorganic and Coordination Compounds: Part A: Theory and Applications in Inorganic Chemistry; John Wiley & Sons, Inc.: New York, United States of America, **2008**.

(10) Mohamed, T. A.; Shabaan, I.A., Zoghaib, W. M., Husband, J., Farag, R. S., Alajhaz, A. E. N. M. A. Tautomerism, normal coordinate analysis, vibrational assignments, calculated IR, Raman and NMR spectra of adenine. *J. Mol. Struct.* **2009**, *938*, 263-276.
